# Using Grid Cells for Navigation

**DOI:** 10.1016/j.neuron.2015.07.006

**Published:** 2015-08-05

**Authors:** Daniel Bush, Caswell Barry, Daniel Manson, Neil Burgess

**Affiliations:** 1 UCL Institute of Cognitive Neuroscience, 17 Queen Square, London, WC1N 3AR, UK; 2 UCL Institute of Neurology, Queen Square, London, WC1N 3BG, UK; 3 UCL Department of Cell and Developmental Biology, Gower Street, London, WC1E 6BT, UK; 4 UCL Centre for Mathematics and Physics in the Life Sciences and Experimental Biology, Gower Street, London, WC1E 6BT, UK

## Abstract

Mammals are able to navigate to hidden goal locations by direct routes that may traverse previously unvisited terrain. Empirical evidence suggests that this “vector navigation” relies on an internal representation of space provided by the hippocampal formation. The periodic spatial firing patterns of grid cells in the hippocampal formation offer a compact combinatorial code for location within large-scale space. Here, we consider the computational problem of how to determine the vector between start and goal locations encoded by the firing of grid cells when this vector may be much longer than the largest grid scale. First, we present an algorithmic solution to the problem, inspired by the Fourier shift theorem. Second, we describe several potential neural network implementations of this solution that combine efficiency of search and biological plausibility. Finally, we discuss the empirical predictions of these implementations and their relationship to the anatomy and electrophysiology of the hippocampal formation.

## Highlights

• Grid cells (GCs) are believed to provide a path integration input to place cells
• However, GCs also provide a powerful context-independent metric for large-scale space
• Hence, we show how GCs can be used for vector navigation between arbitrary locations
• We simulate various neural implementations and make testable experimental predictions

## Teaser

Grid cells are thought to support path integration, but also provide a context-independent metric for large-scale space. Here, Bush et al. show how grid cells could be used for vector navigation and explore the predictions of several potential neural implementations.

## Introduction

It is believed that mammals can use an internal representation of space to navigate directly to goal locations (O’Keefe and Nadel, 1978; Gallistel, 1990) without following explicit sensory cues (Morris et al., 1982) or a well-learned sequence of actions (Packard and McGaugh, 1996). This “vector navigation” problem can be posed in terms of how the representation of a goal location can be combined with that of the current location to infer the vector between the two. Importantly, the resulting trajectory may be novel, having never before been taken by the animal, and could pass through regions of the environment that have not previously been visited (Tolman, 1948). Moreover, this ability does not require learning from reinforcement over multiple trials (e.g., Sutton and Barto, 1998) as it can occur within a single trial (Steele and Morris, 1999), benefit from “latent” learning in the absence of reinforcement (Tolman, 1948; Bendig, 1952; Keith and McVety, 1988), and need not show blocking or overshadowing between multiple cues (Hayward et al., 2003; Doeller and Burgess, 2008).

The ability to perform vector navigation is impaired by bilateral damage to the hippocampal formation (Morris et al., 1982; Parron and Save, 2004; Steffenach et al., 2005; Van Cauter et al., 2013). Similarly, metabolic activity in the human hippocampus correlates with navigational performance (Maguire et al., 1998; Hartley et al., 2003; Iaria et al., 2003), and damage to the hippocampus is associated with impaired spatial navigation (Kolb and Whishaw, 1996; Abrahams et al., 1997; Burgess et al., 2002) in addition to more general mnemonic deficits (Scoville and Milner, 1957; Squire and Zola-Morgan, 1991; Cohen and Eichenbaum, 1993). At the neural level, the mammalian hippocampal formation contains several different representations of self-location and orientation including place cells in the hippocampus proper (O’Keefe and Dostrovsky, 1971; Muller and Kubie, 1987); head direction cells in the subicular complex and deeper layers of mEC (J.B. Ranck, 1984, Soc. Neurosci., abstract; Taube et al., 1990; Sargolini et al., 2006); and grid cells in the superficial layers of mEC, pre- and para-subiculum (Hafting et al., 2005; Sargolini et al., 2006; Boccara et al., 2010). Earlier models of vector navigation generally focused on the well-characterized spatial activity of place cells (e.g., Dayan, 1991; Burgess et al., 1994; Sharp et al., 1996; Touretzky and Redish, 1996; Conklin and Eliasmith, 2005). In smaller environments, place cells typically exhibit a single spatial receptive field, firing whenever the animal enters a specific portion of the environment. As such, a simple way to navigate using place cells is to compare a representation of the goal location with that of the current location and move so as to increase the similarity between the two (Burgess and O’Keefe, 1996).

However, despite providing a potentially useful one-to-one relationship with the locations of specific sensory and affective environmental features, place cell firing patterns do not explicitly represent the structure of space (O’Keefe and Nadel, 1978). There appears to be no consistent relationship between the locations of a place cell’s firing fields in different environments (O’Keefe and Conway, 1978; Thompson and Best, 1989) and no pattern relating the multiple firing fields that a place cell may have in larger environments (Fenton et al., 2008). These properties imply that any mapping between place cell representations and translation vectors used for navigation would have to be re-learned in each new environment. Moreover, navigation using place cell representations is limited in range to the diameter of the largest place fields, unless combined with experience-dependent learning over multiple trials (e.g., Dayan 1991; Blum and Abbott, 1996; Brown and Sharp, 1995; Foster et al., 2000), which will tend to bias behavior toward previously learned routes. Beyond this range, the similarity of the current and goal place cell representations will be zero, providing no gradient in similarity leading to the goal location. Although large place fields have been recorded (∼10 m; Kjelstrup et al., 2008), these properties clearly limit the utility of place cell representations for large-scale vector navigation.

**Figure 1:**
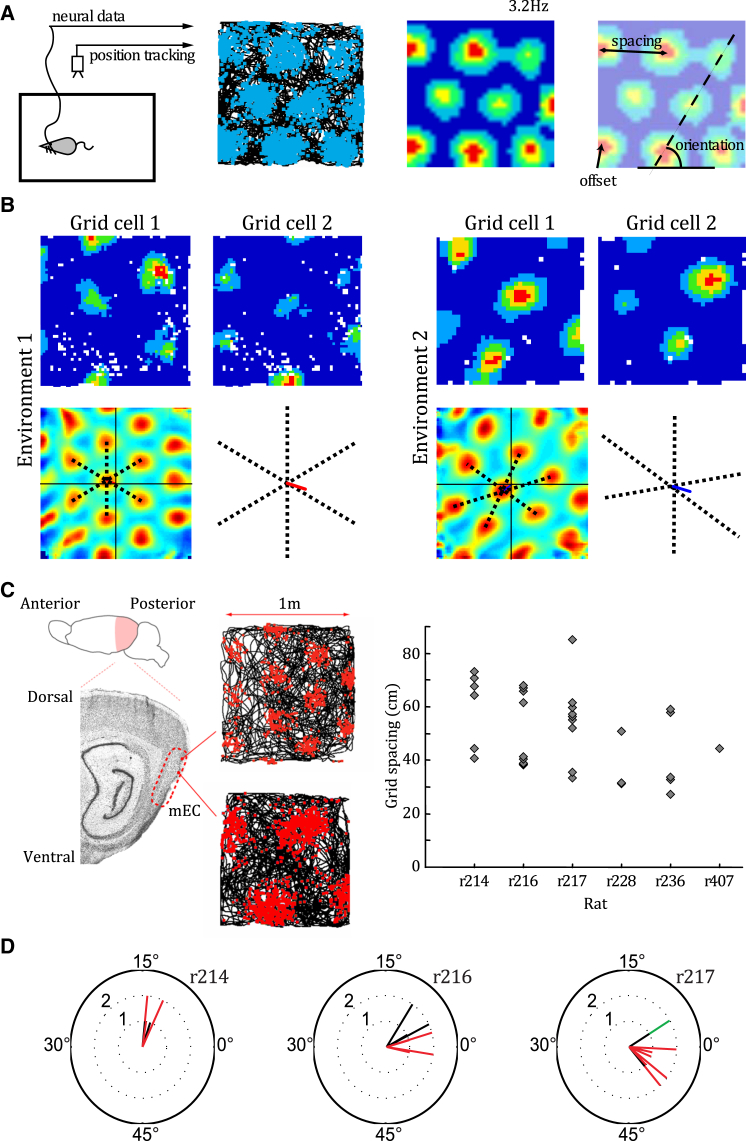
Properties of the Grid Cell System (A) Left: schematic of single unit recording. Middle left: raw data from a sample mEC grid cell. The animal’s path is indicated by the black line, and the positions at which action potentials were fired are superimposed in blue. Middle right: firing rate map for the same mEC grid cell, with high firing rates indicated by “hot” colors. Right: the regular grid-like firing pattern can be characterized by its orientation, scale, and offset or spatial “phase.” (B) Two mEC grid cells co-recorded on a single tetrode in different environments exhibit the same grid scale and orientation but differ in their offset or relative spatial phase. Top row: firing rate maps for a pair of grid cells recorded in a familiar (left) and novel (right) environment. Bottom row: spatial cross-correlation of the grid cell firing rate maps in each environment. Black dashed lines indicate the central six peaks of the cross-correlation; colored line shows the distance and direction from the central peak to the origin of the spatial cross-correlation, after correcting for changes in grid scale and ellipticity. This illustrates that the offset between the firing fields of those two grid cells is preserved between environments, even when the grid pattern has expanded and deformed (adapted from Barry et al., 2012). (C) Grid cells appear to be organized into discrete functional modules whose scale increases in discrete steps along the dorso-ventral axis of mEC (adapted from Barry et al., 2007). (D) Grid field orientation of grid cells recorded in three different rats. The orientations of grid firing patterns are significantly clustered within and between modules. Grid cells with spatial scales that differ by less than 20% are assumed to belong to a single module and grouped by color (adapted from Barry et al., 2007).

In contrast to place cells, grid cells exhibit several properties that afford large-scale vector navigation. Grid cells also show stable spatial firing correlates but with multiple firing fields distributed in a regular hexagonal array that covers all environments visited by the animal (Hafting et al., 2005; Sargolini et al., 2006; Figure 1A). Grid cells are organized into functional modules within medial entorhinal cortex (mEC): cells that are proximate in the brain tend to have firing patterns that share the same scale and orientation but a fixed spatial offset relative to one another (i.e., exhibit a different spatial “phase”; Hafting et al., 2005; Barry et al., 2007; Stensola et al., 2012). Importantly, the relative spatial phase of any two simultaneously recorded grid cells from the same module appears to be conserved across all environments visited by the animal, and a small number of grid firing patterns can completely cover the environment (Hafting et al., 2005; Sargolini et al., 2006; Figure 1B). Grid scale increases between modules in discontinuous steps along the dorso-ventral axis of mEC, with the smallest being around 25 cm and the largest so far recorded exceeding 300 cm and probably representing the fourth or fifth of up to ten discrete scales (Barry et al., 2007; Stensola et al., 2012; Figure 1C). The orientations of grid firing patterns in different modules are also clustered (Barry et al., 2007; Stensola et al., 2012; Figure 1D). It is not yet clear whether grid cells in the pre- and para-subiculum have the same topography (Boccara et al., 2010).

The regular periodic firing patterns of grid cells potentially provide a compact code for location that resembles a residue number system, encoding positions over a very large range that approaches the lowest common multiple of the spatial scales of all grid modules (Gorchetchnikov and Grossberg, 2007; Fiete et al., 2008; Sreenivasan and Fiete, 2011; Mathis et al., 2012). Interestingly, grid cells are widely believed to provide a path integration input to place cells, updating the representation of self-location by a vector describing the animal’s recent motion (Hafting et al., 2005; O’Keefe and Burgess, 2005; McNaughton et al., 2006; Rolls et al., 2006; Solstad et al., 2006). However, by providing a context-independent spatial metric, grid cells also have the potential to solve the inverse problem of vector navigation—to compute a translation vector between current and previously known locations, as opposed to combining a previously known location with the subsequent movement vector to compute the current location. More generally, the periodic firing patterns of grid cells appear to provide a framework with which to infer the vector between two locations, even when those locations are much farther apart than the largest grid scale (Gorchetchnikov and Grossberg, 2007; Fiete et al., 2008; Huhn et al., 2009; Masson and Girard, 2011; Erdem and Hasselmo, 2012; Kubie and Fenton, 2012).

Here, we consider the problem of large-scale vector navigation with grid cells at Marr’s three levels of analysis (Marr and Poggio, 1977). First, we outline the computational problem to be solved: how to compute a translation vector between co-ordinates encoded in an idealized grid cell system, and describe how this relates to the capacity of that system to encode unique locations (see also Gorchetchnikov and Grossberg, 2007; Fiete et al., 2008; Sreenivasan and Fiete, 2011). Second, we describe an algorithmic solution to this problem, based on the grid cell network and inspired by the Fourier shift theorem (see also Orchard et al., 2013). This solution is focused on resolving ambiguity between the multiple, periodic locations represented by activity within each grid module, rather than optimizing the efficiency and accuracy of the grid cell code for location within the scale of the largest grid (for this latter topic, see Mathis et al., 2012, 2013; Wei et al., 2013). Finally, we describe several plausible neural network implementations that use grid cells to calculate the translation vector between start and goal locations in 2D space over distances that can exceed the largest grid scale (see also Fiete et al., 2008; Huhn et al., 2009; Erdem and Hasselmo, 2012; Kubie and Fenton, 2012; Erdem and Hasselmo, 2014). We focus on proposed mechanisms that can perform vector navigation relatively rapidly (i.e., without an exhaustive search of the numerous possible solutions) and that provide experimentally testable predictions.

## Results

### The Computational Problem

#### The Grid Cell Representation of Space

**Figure 2:**
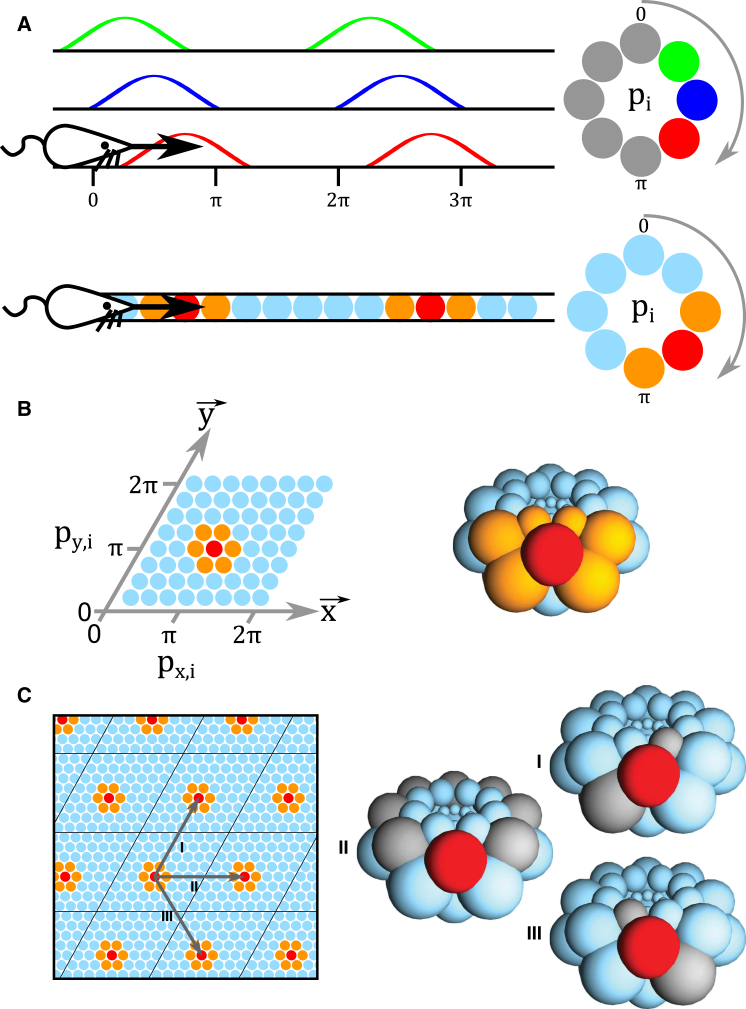
The Grid Cell Representation of Space (A) Top: in 1D, a single module of grid cells encode location with spatially offset, periodic firing fields corresponding to different phases of activity *p*_*i*_ in a ring of cells. Bottom: the position of an animal can therefore be described by the periodic spatial phase *p*_*i*_ that corresponds to a single activity bump (hot colors) moving around the ring of cells according to the animal’s self-motion. (B) In 2D, a single module *i* of grid cells encodes the location of an animal as a pair of spatial phases ${\vec{p}}_{i}=\left(p_{x,i},\;p_{y,i}\right)$ along the grid axes $\vec{x}$ and $\;\vec{y}\;$ (left). These axes define a single, rhombic grid cell tile that can be joined along all edges to create a twisted torus topology (right). (C) Movement along each of the principal axes of the grid field in space (left) corresponds to movement around each of the principal axes of the twisted torus (right) in the grid cell module.

We parameterize the grid cell spatial representation as follows: there are *M* grid cell “modules” with spatial scale *s*_*i*_ (*s*_1_ being the largest and *s*_*M*_ the smallest) that each consist of a topographically ordered population of *m*_*i*_ cells. In 1D space, we can visualize each module of grid cells as a ring that supports a population activity bump centered at phase *p*_*i*_ where 0 ≤ *p*_*i*_ < 2*π* (Figure 2A). In 2D space, we can visualize each module of grid cells as a twisted torus supporting a single activity bump centered at phases $\vec{p_{i}}=\left(p_{x,i},\;p_{y,i}\right)$ along the principal axes of a unitary “tile” of the grid pattern (i.e., unit vectors $\vec{x}$ and $\vec{y}$; see Figures 2B and 2C; Guanella et al., 2007). Note that one can choose any two non-collinear axes to define the grid phase and corresponding unit tile (Kubie and Fenton, 2012) but, for simplicity, we consider two of the axes of symmetry of the grid pattern so that grid scale is equal on each. Moreover, twisted torus connectivity is only necessary when considering grid cell activity as a single bump—other network topologies can account for the grid cell firing pattern when multiple activity bumps are present (e.g., Fuhs and Touretzky, 2006; Burak and Fiete, 2009). Finally, increasing the number of grid cells within a module improves precision, but not the amount of information encoded beyond the two degrees of freedom needed to define the animal’s location within the corresponding tile.

#### The Vector Navigation Problem

**Figure 3:**
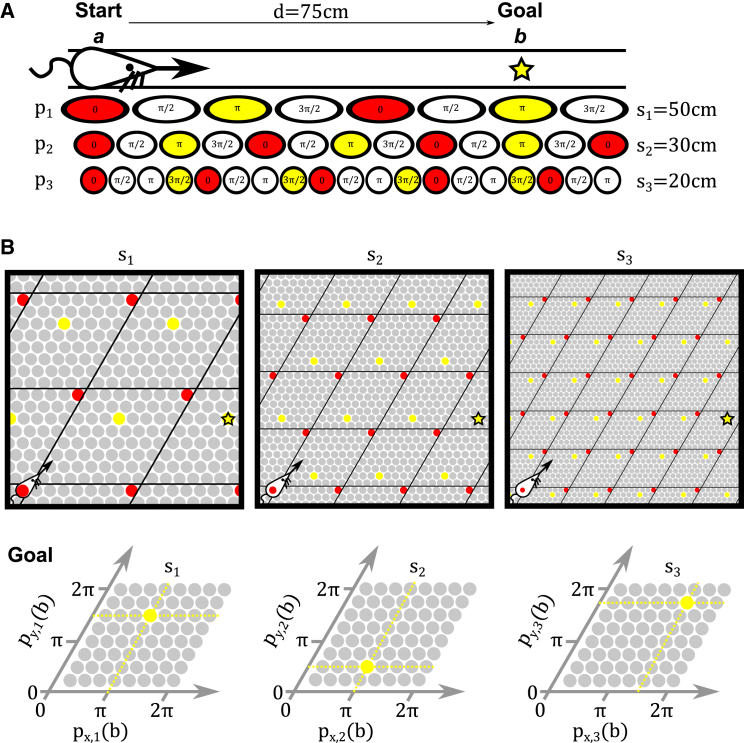
The Problem of Vector Navigation with Grid Cells (A) In 1D, how do we find the displacement *d* between the starting location *a* (red) and goal location *b* (yellow) given the grid cell representations of those locations (i.e., sets of spatial phases across grid modules {*p*_*i*_(*a*)} and {*p*_*i*_(*b*)})? (B) In 2D, how do we find the distance and direction between start and goal locations given the grid cell representations of those locations (i.e., sets of spatial phases across grid modules and principal axes: $\left\{p_{x}\left(\vec{a}\right),\;p_{y}\left(\vec{a}\right)\right\}$, $\left\{p_{x}\left(\vec{b}\right),\;p_{y}\left(\vec{b}\right)\right\}$)?

The 1D vector navigation problem can be stated thus: given the grid cell representations of two locations *a* and *b*, calculate the displacement between those locations *d* = *b* − *a* (Figure 3A). More specifically, the grid cell representations of locations *a* and *b* correspond to the spatial phases of activity bumps in each grid module {*p*_*i*_(*a*) | *i* = 1 *to M*}={*p*_1_(*a*),*p*_2_(*a*),…,*p*_*M*_(*a*)} and {*p*_*i*_(*b*) | *i* = 1  *to M*}={*p*_1_(*b*),*p*_2_(*b*),…,*p*_*M*_(*b*)}. As an example, consider three grid cell modules with scales *s*_1_ = 50 *cm*, *s*_2_ = 30 *cm* and *s*_3_ = 20 *cm*. If the distance between the current location *a* and goal location *b* is *d* = 75 *cm*, and (for the sake of simplicity, but without loss of generalization) the phase of each module is 0 at the current location *a*—i.e., *p*_1_(*a*) = 0, *p*_2_(*a*) = 0 and *p*_3_(*a*) = 0—then at *b* the phase of each module will be proportional to the distance *d* modulo grid scale *s*_*i*_:

$$p_{1}\left(b\right)=\frac{75\;mod\;50}{50}\times 2\pi =\pi$$

$$p_{2}\left(b\right)=\frac{75\;mod\;30}{30}\times 2\pi =\pi$$

$$\;p_{3}\left(b\right)=\frac{75\;mod\;20}{20}\times 2\pi =\frac{3\pi }{2}.$$

The 1D vector navigation problem is to recover the displacement *d* from these values of {*p*_*i*_(*b*)} (Figure 3A).

Similarly, the 2D vector navigation problem can be stated thus: given the grid cell representations of two locations $\vec{a}$ and $\vec{b}$, calculate the displacement vector between those locations $\vec{d\;}=\vec{b}-\vec{a}$. More specifically, the grid cell representations of locations *a* and *b* correspond to the sets of spatial phases $\left\{p_{x,i}\left(\vec{a}\right)\right\}=\left\{p_{x,1}\left(\vec{a}\right),\;p_{x,2}\left(\vec{a}\right),\ldots ,p_{x,M}\left(\vec{a}\right)\;\right\}\;$ and $\left\{p_{y,i}\left(\vec{a}\right)\right\}=\left\{p_{y,1}\left(\vec{a}\right),\;p_{y,2}\left(\vec{a}\right),\ldots ,p_{y,M}\left(\vec{a}\right)\;\right\}$ that define position $\vec{a}$ in module *i* along principal axes *x* and *y* (which, in this case, are separated by 60°; see Figures 2B and 2C). Again, consider three grid cell modules with scales *s*_1_ = 50 *cm*, *s*_2_ = 30 *cm* and *s*_3_ = 20 *cm*. If the displacement vector between the current location $\vec{a}$ and goal location $\vec{b}$ is $\vec{d}=\left(75\;cm,\;37.5\;cm\right)$, and the phase of each module on each axis is 0 at the current location, then at $\vec{b}$ the phases of the modules will be:

$$\left\{p_{x,i}\left(\vec{b}\right)\right\}=\left\{\pi ,\pi ,\frac{3\pi }{2}\right\}$$

$$\left\{p_{y,i}\left(\vec{b}\right)\right\}=\left\{\frac{3\pi }{2},\frac{\pi }{2},\frac{7\pi }{4}\right\}.$$

The 2D vector navigation problem is to recover the displacement vector $\vec{d}$ from $\left\{p_{x,i}\left(\vec{b}\right)\right\}$ and $\left\{p_{y,i}\left(\vec{b}\right)\right\}$ (Figure 3B). Note that this corresponds to a simple generalization of the 1D vector navigation problem to multiple axes.

### Algorithmic Solution in 1D

The cyclical nature of the grid representation within each module *i* is such that an activity bump at phase *p*_*i*_ implicitly represents an infinite set of “unwrapped” phases *p*_*i*_ + 2*πn*_*i*_, where *n*_*i*_ can take any integer value, corresponding to an infinite set of distances *s*_*i*_(*p*_*i*_/2*π* + *n*_*i*_) along that 1D axis that are separated by the scale *s*_*i*_ of module *i*. Initially, we assume that all phases are zero at the current location *a* and the distance *d* to a goal location *b* must be inferred from the grid cell representation across modules at that location {*p*_*i*_(*b*)} = {*p*_1_(*b*), *p*_2_(*b*),…,*p*_*M*_(*b*)}. The grid representation of the goal location *b* is such that there is a set of unwrapped phases (one for each module) that explicitly represent the same distance—i.e., there is a set of integers {*n*_*i*_} for which:

(Equation 1) $$d=s_{i}\left(\frac{p_{i}}{2\pi }+n_{i}\right)\text{for}\;\text{all}\;i.$$

**Figure 4:**
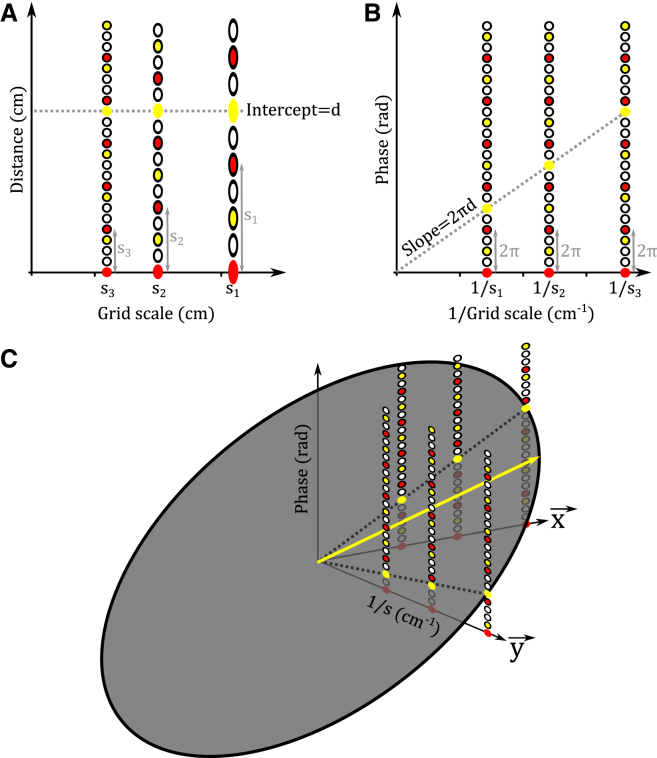
Algorithmic Solution to the Problem of Navigation with Grid Cells (A) In 1D, if we assume that the phase of all grid modules at the starting location *a* is zero, then the “unwrapped” set of periodic displacements corresponding to the goal location *b*—i.e., *s*_*i*_(*p*_*i*_(*b*)/2*π* + *n*_*i*_), where *s*_*i*_ is the grid scale and *n*_*i*_ an integer for module *i*—fall on a horizontal line *y* = *d* corresponding to the goal location (gray dashed line). (B) Similarly, the “unwrapped” set of spatial phases across modules *p*_*i*_(*b*) + 2*πn*_*i*_, when plotted against inverse grid scale 1/*s*_*i*_, fall on a straight line through the origin with gradient 2*πd* (gray dashed line). (C) In 2D, the “unwrapped” set of spatial phases corresponding to the goal location across modules {*p*_*x*,*i*_(*b*) + 2*πn*_*x*,*i*_, *p*_*y*,*i*_(*b*) + 2*πn*_*y*,*i*_}, when plotted against inverse grid scale 1/*s*_*i*_ along the principal axes $\vec{x}$ and $\vec{y}$, fall on a plane through the origin (gray ellipse) whose maximum slope (within the capacity of the grid cell system) corresponds to the distance and direction to the goal location (yellow arrow).

Graphically, this coherent set of unwrapped phases across modules falls on a horizontal line when plotted against a y axis of represented distance (i.e., *y* = *d*; Figure 4A) or, equivalently, on a straight line through the origin when plotted against a y axis of phase against inverse grid scale (Equation 2; Figure 4B)—i.e., there is a set of integers {*n*_*i*_} for which:

(Equation 2) $$p_{i}+2\pi n_{i}=2\pi d\left(\frac{1}{s_{i}}\right)\text{for}\;\text{all}\;i.$$

This latter relationship is obtained by re-arranging Equation 1 and corresponds to the Fourier shift theorem (see discussion of the Algorithmic Solution in 2D below; Orchard et al., 2013). Thus, the distance *d* to a goal location represented by the set of module phases {*p*_*i*_} can be inferred by fitting a straight line through the origin on a plot of unwrapped phases *p*_*i*_ + 2*πn*_*i*_ against inverse grid scale 1/*s*_*i*_ across modules (Figure 4B). Moreover, this result can be generalized to any pair of arbitrary current and goal locations on that 1D axis, by replacing the absolute phase *p*_*i*_ at the goal location with the phase difference *Δp*_*i*_ between grid cell representations of current and goal locations in each module (Equation 3):

$$d=b-a=\;s_{i}\left(\frac{p_{i}\left(b\right)}{2\pi }+n_{i}\left(b\right)\right)-s_{i}\left(\frac{p_{i}\left(a\right)}{2\pi }+n_{i}\left(a\right)\right)\text{for}\;\text{all}\;i$$

(Equation 3) $$\Delta p_{i}+2\pi n_{i}=2\pi d\left(\frac{1}{s_{i}}\right)\text{for}\;\text{all}\;i.$$

It is important to note that Equations 1 and 2 describe an underdetermined system, as there are more unknowns (*M* + 1, corresponding to *n*_*i*_ and *d*) than equations (*M*, one for each grid module). Hence, multiple possible solutions *d*(*k*) exist for each unique combination of phase values {*p*_*i*_} or phase differences {*Δp*_*i*_} across modules, such that one set of grid cell phases across modules represents more than one, periodically spaced location in the real world (Figure S1). The capacity of the grid cell system is defined as the maximum spatial range within which each combination of phase values {*p*_*i*_} (or phase differences {*Δp*_*i*_}) corresponds to a unique decoded location (or displacement)—i.e., the distance between locations encoded by the same set of grid cell phases or the period of the grid cell system as a whole. Theoretical studies suggest that this capacity is much greater than the typical foraging range of an animal (Gorchetchnikov and Grossberg, 2007; Fiete et al., 2008; Sreenivasan and Fiete, 2011; Mathis et al., 2012; see Supplemental Experimental Procedures). Beyond that capacity, the spatial representation provided by the grid cell network as a whole is periodic. Hence, Equations 1 and 2 only convert the spatial representation between residue-like and linear number systems within this capacity, and more generally convert between two residue-like number systems—one with a discrete set of smaller spatial scales and one with a single, much larger spatial scale.

### Algorithmic Solution in 2D

In 2D, the location of an activity bump can be defined by considering any two non-collinear axes (denoted by unit vectors $\vec{x}$ and $\vec{y}$). An activity bump at phase $\vec{p_{i}}=\left(p_{x,i},\;p_{y,i}\right)$ in module *i* then maps onto an infinite series of periodic locations $s_{i}\left(\left(p_{x,i}/2\pi \right)+n_{x,i}\right)\vec{x}$ + $s_{i}\left(\left(p_{y,i}/2\pi \right)+n_{y,i}\right)\vec{y}$, where $\left\{\vec{n_{i}}\right\}=\left\{n_{x,i},\;n_{y,i}\right\}$ can be any pair of integers. We initially assume, for simplicity, that the orientations of different grid modules are aligned, i.e., $\vec{x}$ and $\vec{y}$ are independent of *i* (Barry et al., 2007; Stensola et al., 2012) and that grid firing fields are circularly symmetric as opposed to elliptical, i.e., the scale *s*_*i*_ is the same for directions $\vec{x}$ and $\vec{y}$ (but see Stensola et al., 2012). In this case, the location of an activity bump within grid module *i* can be visualized on a cylindrical polar plot as $\left(r,\theta ,z\right)=\left(\left(1/s_{i}\right),\;arg\left(\vec{u_{j}}\right),\;p_{j,i}+2\pi n_{j,i}\right)$, where $arg\left(\vec{u_{j}}\right)$ represents the direction of the grid axes (e.g., $\vec{u_{1}}=\vec{x}$ and $\vec{u_{2}}=\vec{y}$). Considering the phases of the goal representation along each axis, and following the logic of the 1D solution, the distance and direction to the goal location is indicated by the maximum gradient of a plane through the origin that fits the phase points *p*_*j*,*i*_ + 2*πn*_*j*,*i*_ for all modules *i* and axes *j* and lies within the capacity of the grid cell system (Figure 4C).

Again, this result can be generalized to arbitrary current and goal locations by replacing the absolute phases *p*_*j*,*i*_ that define the goal location with the phase difference *Δp*_*j*,*i*_ between the current and goal locations on each axis and in each module. This solution is consistent with the Fourier shift theorem (see Orchard et al., 2013), with the sets of grid cells in each module that share a common phase on each axis acting as Fourier components of the spatial representation. If grid cell orientations are identical across modules, then the displacement between start and goal locations can be solved independently on each axis as in the 1D case: by finding the line through the origin that best fits the phase points along that axis. We note that it is sufficient to solve for two directions, as more axes do not provide additional independent information—the constraint that $\sum_{j}p_{j,i}=0$ for 3 directions separated by 120° (Burgess and Burgess, 2014) is implicitly included by fitting lines through the origin. However, given independent noise in the firing rates of biological neurons, pooling estimates across directions could potentially mitigate error in the extracted translation vector. Additionally, if grid cell orientations are not conserved across modules, or if grid firing fields are elliptical, then the solution still holds, but the plane must be fit to a family of phase points that differ in axes $\vec{u_{j,i}}$ across grid cell modules and in spatial scale *s*_*i*_ across axes.

### Neural Network Implementations

There are many potential neural network implementations of vector navigation using grid cells, which exhibit varying degrees of efficiency, parsimony, and biological plausibility. Here, building on previous work (e.g., Sun and Yao, 1994; Gorchetchnikov and Grossberg, 2007; Fiete et al., 2008; Huhn et al., 2009; Mhatre et al., 2012; Masson and Girard, 2011; Erdem and Hasselmo, 2012; Kubie and Fenton, 2012; Erdem and Hasselmo, 2014), we describe two broad classes of solution and present neural network simulations that demonstrate the potential accuracy with which they can compute translation vectors between arbitrary locations in large-scale space (see Supplemental Information for details). The first class of solution uses additional neural circuitry to directly decode grid cell activity at current and goal locations and then read out the distance between those locations along specific 1D axes, effectively converting the grid cell residue-like number system to a linear spatial metric (see also Sun and Yao, 1994; Fiete et al., 2008; Huhn et al., 2009; Masson and Girard, 2011). The second class of solution uses network dynamics to perform sequential, directed searches along specific 1D axes, the search being initiated from either the current or goal location in order to ascertain the distance between those locations (see also Erdem and Hasselmo, 2012; Kubie and Fenton, 2012; Erdem and Hasselmo, 2014). Having described each neural network implementation, we discuss their relative strengths and weaknesses as well as the experimental predictions they make.

#### Vector Navigation by Direct Decoding: The “Distance Cell” Model

**Figure 5:**
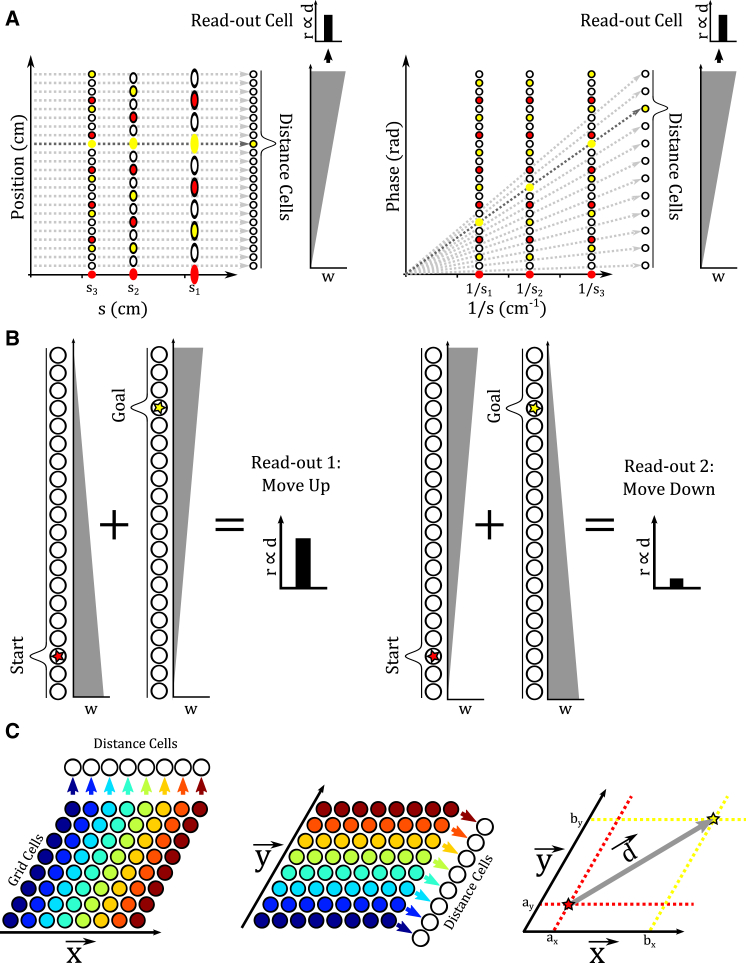
The Distance Cell Model (A) An array of distance cells encode specific locations along a one-dimensional axis and receive input from grid cells that are active at each location along that axis. When grid cells that encode a goal location in each module fire, they activate a single distance cell that encodes that location, and winner-take-all dynamics eliminates activity in other distance cells. All distance cells provide input to a single readout cell with synaptic strengths that increase linearly with increasing displacement along the axis. The firing rate of that readout cell then encodes the displacement from the origin to the goal location along that axis. (B) Combining two distance cell arrays allows the distance between arbitrary start and goal locations in either direction along the axis to be decoded. One distance cell array decodes the start location, and the other decodes the goal location. Each distance cell array projects to one “move up” (left) and one “move down” (right) readout cell with synaptic weights *w* that increase linearly in opposing directions along the axis. These readout cells then encode the displacement between start and goal locations in each direction along that axis. (C) The distance cell model can be extended to two dimensions if all grid cells that share a common phase on each of at least two non-collinear axes project to the same distance cell. Combining the displacements encoded by the pair of readout cells for each axis provides the vector between start and goal locations in two-dimensional space. For full simulation details, see Supplemental Experimental Procedures and Figure S2.

The “distance cell” model decodes both the absolute current and goal locations from rate-coded modular grid cell representations and then calculates the translation vector between those locations. An array of distance cells each encode a unique location *a* along a single directional axis $\vec{x}$ (see also Fiete et al., 2008; Huhn et al., 2009). Distance cells receive input from grid cells in each module with synaptic weights proportional to their mean firing rate at that location *a* on the axis $\vec{x}$. Hence, each distance cell is maximally activated by a specific set of phase values across grid cell modules {*p*_*x*,*i*_(*a*)} (Figure 5A), and winner-take-all dynamics within each distance cell array prevents firing in distance cells that receive lower levels of input. The total number of distance cells is limited by the capacity of the grid cell system to encode locations as unique sets of phases across grid cell modules {*p*_*x*,*i*_(*a*)} (see Supplemental Experimental Procedures; Figure S1), and all potential locations within that capacity are encoded by a distance cell. All distance cells provide input to a readout neuron with synaptic weights that increase in strength with increasing distance along the axis. The firing rate of this readout neuron then signals the distance from the origin to that location along the directional axis $\vec{x}$ (Figure 5A).

The distance cell model can be extended to deal with arbitrary start and goal locations along the axis $\vec{x}$ by incorporating an additional array of distance cells and an additional readout cell, analogous to neural network models of the mental number line (Dehaene, 1997; Chen and Verguts, 2010). In this case, one distance cell array decodes current location *a* and the other decodes goal location *b* (Figure 5B). Both distance cell arrays project to both readout cells, but the strength of connections from each distance cell array to each readout cell increases in opposite directions along the axis $\vec{x}$. The relative firing rates of the two readout cells then encode the relative distance between current and goal locations along that axis in each direction (Figure 5B). Translation vectors in 2D space can be constructed from at least two pairs of distance cell arrays that decode current and goal positions $\vec{a}$ and $\vec{b}$ on non-collinear axes $\vec{x}$ and $\vec{y}$. In this case, each distance cell on each axis receives input from all grid cells within a module that share a common phase on that axis (Figure 5C). This model can also accommodate grid modules that differ in their ellipticity and orientation, provided that synaptic connections between grid cells and distance cells accurately project the position encoded by those grid cells onto the distance cell axis. Simulations demonstrate that the distance cell model can accurately decode translation vectors between arbitrary start and goal locations in large-scale 2D space (see Supplemental Experimental Procedures; Figure S2).

#### Vector Navigation by Direct Decoding: The “Rate-Coded Vector Cell” Model

**Figure 6:**
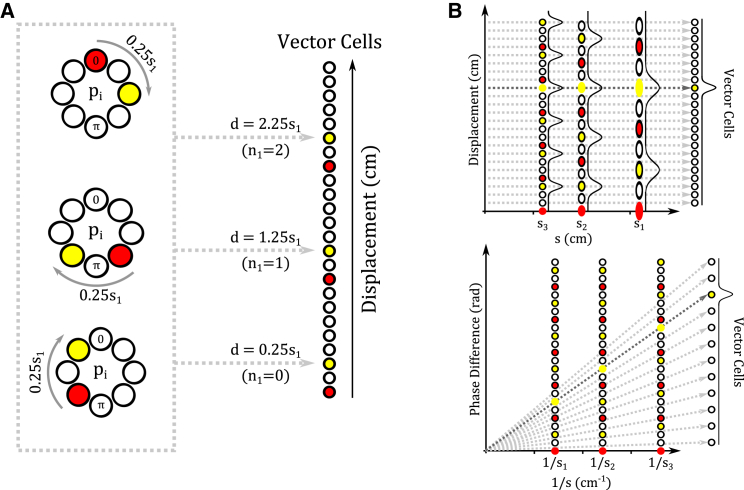
The Rate-Coded Vector Cell Model (A) An array of vector cells encode specific displacements *d* along a one-dimensional axis $\vec{x}$ and receive input from pairs of grid cells within each module *i* encoding current (red) and goal (yellow) locations whose unwrapped phase difference *Δp*_*x*,*i*_ corresponds to that displacement, i.e., *d* = ((*Δp*_*x*,*i*_/2*π*) + *n*_*i*_)*s*_*i*_ for some integer *n*_*i*_, where *s*_*i*_ is the grid scale. (B) When grid cells encoding current (red) and goal (yellow) locations in each module fire simultaneously, they activate a single vector cell that encodes the consistent displacement across modules, and winner-take-all dynamics eliminates activity in other vector cells. Combining the activity of vector cells across at least two non-collinear axes provides the overall translation vector between start and goal locations in two-dimensional space. For full simulation details, see Supplemental Experimental Procedures and Figure S3.

The distance cell model independently decodes current and goal locations from sets of grid cell phases across modules {*p*_*x*,*i*_} before computing the linear displacement between them. As an alternative, it is possible to decode the linear displacement directly from the set of phase differences between grid cell representations at current and goal locations across modules {*Δp*_*x*,*i*_}. In this “rate-coded vector cell” model, an array of vector cells each encode a specific displacement *d* from the current position along a single directional axis $\vec{x}$. Each vector cell receives input from all pairs of grid cells within each module whose unwrapped spatial phase difference *Δp*_*x*,*i*_ along the axis $\vec{x}$ corresponds to that displacement, i.e., *d* = ((*Δp*_*x*,*i*_/2*π*) + *n*_*i*_)*s*_*i*_ for some integer *n*_*i*_, through multiplicative synapses (Figure 6A). Vector cells also receive input from all grid cell pairs in other modules whose unwrapped phase difference corresponds to the same absolute displacement *d*. Hence, each vector cell is maximally activated by a specific set of phase differences between current and goal locations across grid cell modules along axis $\vec{x}$ {*Δp*_*x*,*i*_} (Figure 6B). The total number of vector cells is limited by the capacity of the grid cell system to encode different displacements with unique sets of phase difference values across grid cell modules {*Δp*_*i*_} (see Supplemental Experimental Procedures; Figure S1). When grid cells encoding the current and goal locations across modules are simultaneously activated, winner-take-all dynamics ensure that only a single vector cell corresponding to the distance and direction between those locations becomes active. Translation vectors in 2D space can be constructed from at least two pairs of vector cell arrays that encode displacements in each direction on non-collinear axes $\vec{x}$ and $\vec{y}$. Simulations demonstrate that the rate-coded vector cell model can accurately decode translation vectors between arbitrary start and goal locations in large-scale 2D space (see Supplemental Experimental Procedures; Figure S3).

#### Vector Navigation by Direct Decoding: The “Phase-Coded Vector Cell” Model

**Figure 7:**
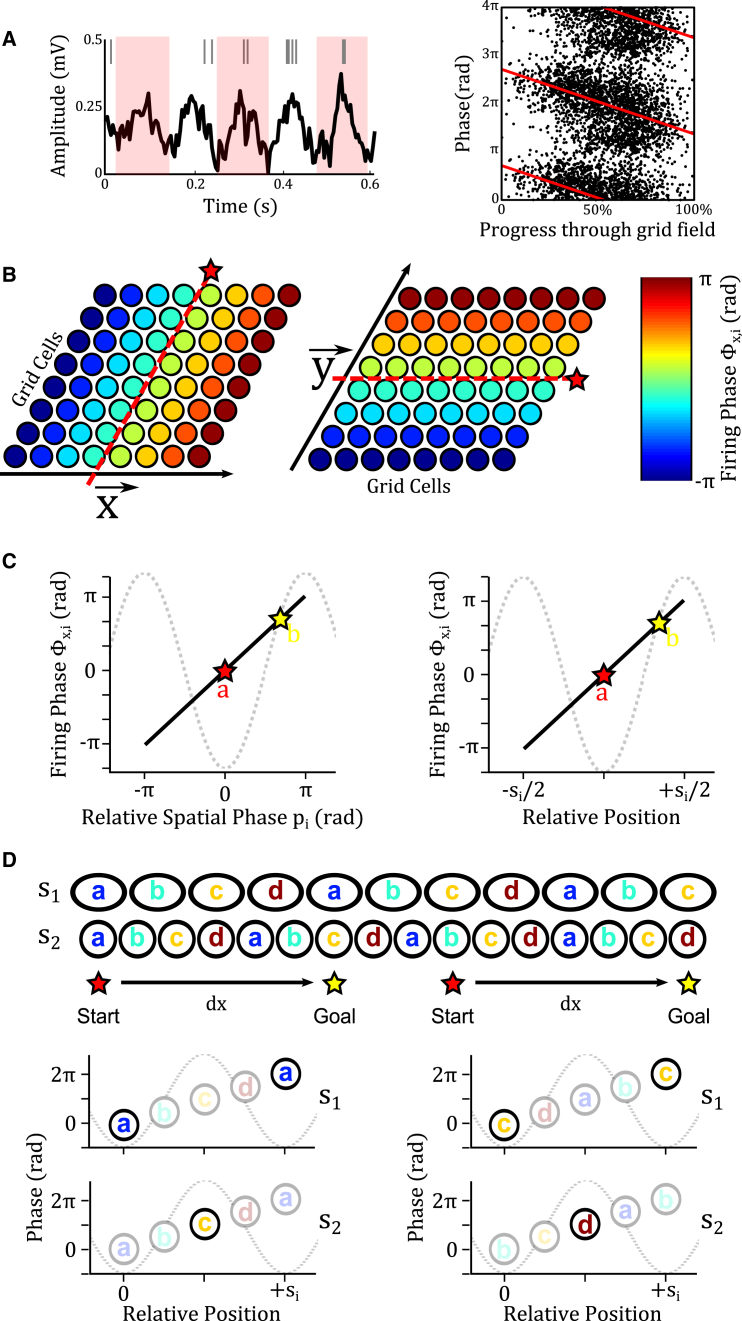
The Phase-Coded Vector Cell Model (A) As animals transit through a firing field, a large proportion of grid cells exhibit theta phase precession, firing spikes progressively earlier relative to the 5–11 Hz theta oscillation in the local field potential. This results in an approximately linear relationship between firing phase and progress through the grid field. (B) If phase precession in grid cells is aligned with a specific one-dimensional axis—that is, theta firing phase encodes the distance traveled through the grid module along that axis, regardless of the trajectory taken—then theta firing phase can be used to infer the relative spatial phase of any two grid cells within a module along that axis. Red dashed line/star indicates the current location on each axis. (C) Phase precession aligned with a specific one-dimensional axis ensures that the difference in theta firing phase between grid cells encoding the current location *a* (which will be ∼0 radians) and a goal location *b* is proportional to the difference in their spatial phase *Δp*_*i*_ or relative position within each grid module. Hence, if the goal location *b* is less than half a grid scale ahead of the current location *a* (as shown here), then grid cells encoding that location will fire at a later theta phase. (D) The set of grid cells across modules *i =* 1 to *M* that encode a goal location at a set displacement from the current location along a specific one-dimensional axis will always fire at a specific combination of theta phase values $\left\{{Ø}_{x,i}\right\}$, irrespective of the current location. Here, we plot the theta firing phase at the current location of grid cells in two modules with scales *s*_*i*_ = {30, 20 cm} against the location of their firing fields relative to the current location. Grid cells encoding the current (“start”) location always fire at the trough of theta (i.e., *0* rad), while grid cells encoding a goal location that is d = 30 cm from the current location along that axis are always encoded by grid cells firing at phases ${Ø}_{x}$ = {0, π rad}, respectively. Hence, an array of vector cells that are sensitive to specific combination of phase values ${Ø}_{x}$ across modules can decode the distance to the goal location along that axis. For full simulation details, see Supplemental Experimental Procedures and Figure S4.

As an alternative to the firing rate model described above, vector cells could make use of the temporal code for location provided by theta phase precession in grid cells. As animals transit through a firing field, a large proportion of grid cells fire spikes progressively earlier relative to the 5–11 Hz theta oscillation in the local field potential (LFP; Hafting et al., 2008; Reifenstein et al., 2012; Climer et al., 2013; Jeewajee et al., 2014; Figure 7A). In place cells, phase precession is stable across trials, while firing rates vary from trial to trial (Fenton and Muller, 1998; Huxter et al., 2003); and in both place and grid cells, phase precession scales with the size of firing fields (Huxter et al., 2003; Climer et al., 2013; Jeewajee et al., 2014) and conveys information about an animal’s location beyond that encoded by the firing rate alone (Jensen and Lisman, 2000; Reifenstein et al., 2012). Importantly, phase precession dictates that the location of each grid field relative to the current location—i.e., the spatial phase difference *Δp*_*i*_—is encoded in the theta firing phase of the corresponding grid cells.

Consider a population of grid cells that exhibit phase precession aligned with a specific 1D axis $\vec{x}$—that is, their theta firing phase encodes the distance traveled through the grid module along that axis, regardless of the trajectory taken (see Figure 7B; Climer et al., 2013; Jeewajee et al., 2014). Under these circumstances, the spatial phase difference between current location *a* and goal location *b* along that axis in module *i* (*Δp*_*x*,*i*_; see Figure 2A) is proportional to the difference in theta firing phases of grid cells encoding the current location *GC*_*a*_ and goal location *GC*_*b*_ in that module, $\emptyset_{i}\left(GC_{a}\right)$ and $\emptyset_{i}\left(GC_{b}\right)$, i.e., $\Delta p_{x,i}\propto \Delta \emptyset_{i}$. If we assume that grid cells encoding the current location consistently fire at the trough of theta (i.e., ∼0 rad), then the relative spatial phase of grid cells encoding the goal location *b* within each module will be proportional to their theta firing phase, i.e., $\left(\left(b-a\right)mod\;s_{i}/s_{i}\right)\propto \emptyset_{i}\left(GC_{b}\right)$ (Figure 7C). Hence, the spatial phase difference *Δp*_*x*,*i*_ between grid cells encoding current and goal locations within each module will also be proportional to the theta firing phase of grid cells encoding the goal location, i.e., $\Delta p_{x,i}\propto \emptyset_{i}\left(GC_{b}\right)$. Vector cells that are sensitive to a specific pattern of spike phases in grid cells encoding the goal location across modules can therefore directly decode the displacement between current and goal locations.

As an example, consider two grid cell modules of scales *s*_1_ = 30 *cm* and *s*_2_ = 20 *cm* on the 1D axis $\vec{x}$ and, for simplicity, assume that phase precession is linear and covers the full range of theta phase values—from *π* rad at field entry, through 0 rad at the field center to −*π* rad at the exit. If the current location is *a* = 0 *cm*, then grid cells encoding a goal location at *b* = 30 *cm* will fire at $\left\{\emptyset_{i}\left(GC_{b}\right)\right\}=\left\{0,\pi \;rad\right\}$ in the two modules, corresponding to the phase difference between their firing fields within each module *Δp*_*x*,*i*_ (Figure 7D). Similarly, if the current location is *a* = 45 *cm*, then grid cells encoding a goal location with the same displacement, i.e., *b* = 75 *cm*, will again fire at $\left\{\emptyset_{i}\left(GC_{b}\right)\right\}=\left\{0,\pi \;rad\right\}$, corresponding to the same phase difference between their firing fields within each module *Δp*_*x*,*i*_ (Figure 7D). Hence, if only grid cells encoding the goal location fire in a single theta cycle, then a vector cell that is sensitive to this specific pattern of firing phases in grid cells encoding the goal location across modules—i.e., $\left\{\emptyset_{i}\left(GC_{b}\right)\right\}$—can directly decode the displacement between current and goal locations. More generally, this result implies that the distance from the current position to every known goal location along that axis is encoded in each theta cycle by the relative phase of firing in grid cells across modules that encode those locations $\left\{\emptyset_{i}\left(GC_{b}\right)\right\}$. Translation vectors between current and goal locations in 2D space can be decoded from the pattern of firing phases in separate populations of grid cells that exhibit phase precession aligned with two non-collinear axes $\vec{x}$ and $\vec{y}$ (Figure 7B). Simulations demonstrate that the phase-coded vector cell model can accurately decode translation vectors between arbitrary start and goal locations in large-scale 2D space (see Supplemental Experimental Procedures; Figure S4).

#### Vector Navigation by Directed Search: The “Linear Look Ahead” Model

**Figure 8:**
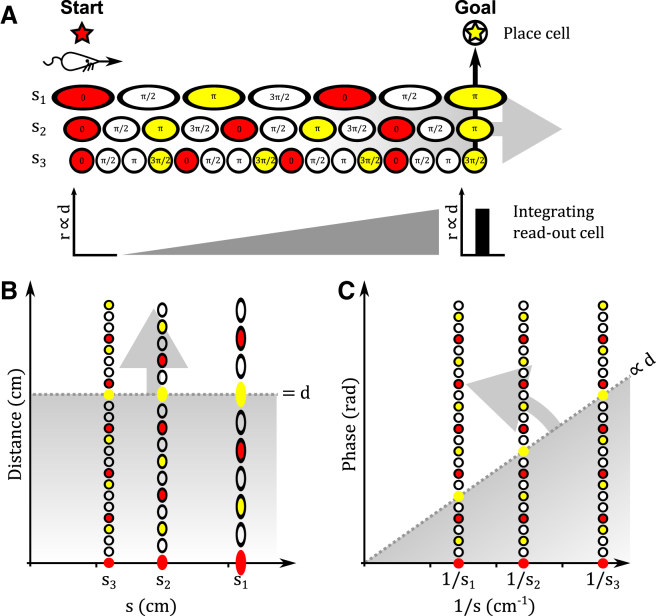
The Linear Look-Ahead Model (A) During linear look-ahead events, activity in the grid cell network is initiated at the current (or goal) location and updated according to simulated movement away from that location along a specific one-dimensional axis. The firing rate of a single readout cell that integrates activity in one or more grid cell modules over time will then indicate the distance traveled along that axis. Arrival at the goal (or current) location is signaled by simultaneous activity in grid cells representing that location across modules. (B and C) Linear look-ahead effectively performs a directed search for the goal (or current) location, starting from the current (or goal) location and moving sequentially through locations of increasing displacement along that directional axis. For full simulation details, see Supplemental Experimental Procedures and Figure S5.

An alternative to directly decoding the translation vector between current and goal locations is a directed search along specific 1D axes, beginning at either of those locations, in order to compute their relative position. During exploration, activity in the grid cell network is believed to reflect an animal’s estimate of self-location that is updated by self-motion signals (Fuhs and Touretzky, 2006; McNaughton et al., 2006; Burgess et al., 2007). However, it is possible that simulated movement signals, decoupled from the animal’s actual motion, could also be used to update the grid cell spatial representation, e.g., perform a “linear look ahead” (Erdem and Hasselmo, 2012; Kubie and Fenton, 2012; Erdem and Hasselmo, 2014) by simulating movement away from the current position *a* along an arbitrary axis $\vec{x}$ at a constant speed (Figure 8A). In the 1D case, this is equivalent to shifting the activity bump within each grid cell module around a putative ring attractor circuit at a rate corresponding to a constant spatial velocity across grid cell modules (i.e., faster for smaller scale modules; Figure 2A). The duration of the linear look ahead event, or the activity of a neuron that integrates total activity during the event, then encodes the displacement *d* of the represented location along the direction $\vec{x}$. The displacement of the goal location along that axis is signaled by simultaneous activity in grid cells encoding the goal location in each module, which could be achieved by coincidence detection in the corresponding place cell, for example (Figure 8A).

In effect, linear look ahead systematically searches for a match between phase values across modules {*p*_*x*,*i*_} that encode the goal location on the axis $\vec{x}$ with phase values that encode a sequence of positions moving away from the current location (Figures 8B and 8C). Alternatively, linear look ahead could be initiated from the goal location and systematically search for a match with phase values across grid modules that match the current location. Translation vectors in 2D space can be constructed using linear look ahead in each direction along at least two non-collinear axes, during which all grid cells that share a phase {*p*_*j*,*i*_} on each axis in each module are simultaneously active (Figure 5C). Hence, during each unidirectional linear look ahead, different sub-populations of grid cells fire simultaneously, according to their spatial phase on that axis. Simulations demonstrate that the linear look-ahead model can accurately decode translation vectors between arbitrary start and goal locations in large-scale 2D space (see Supplemental Experimental Procedures; Figure S5).

#### Critique of Grid Cell Vector Navigation Models

The direct decoding and linear look-ahead models described above exhibit divergent strengths and weaknesses and make different predictions for future experimental studies. Direct decoding models compute translation vectors quickly, without the need to search multiple possible solutions, while the linear look-ahead model predicts that the time required to compute translation vectors scales with their length, because the directed search takes longer to reach more distant locations (Figure S5C). This latter pattern might be more consistent with reports that human response times correlate with the length of imagined paths (Kosslyn et al., 1978) and metabolic activity in the hippocampal formation correlates with the distance to a goal during route planning (Sherrill et al., 2013; Howard et al., 2014).

Each of the direct decoding models requires significant additional neural circuitry to compute translation vectors. This raises the question as to how this circuitry develops or is learned during active navigation but provides experimental predictions regarding the existence of distance and readout or vector cell firing patterns. The distance cell model, for example, requires one or more neurons to encode each unique current and goal location on at least two principal axes, or at least four times as many distance cells as potential locations. These distance cells would exhibit a band-like firing pattern, as they encode a series of known allocentric locations along a specific 1D axis (Figure 5C) with a spatial periodicity equal to the capacity of the grid cell system. Synaptic connections from grid to distance cells could develop under a straightforward Hebbian learning rule during exploration, analogous to models of the grid to place cell transformation (Rolls et al., 2006; Solstad et al., 2006). Graded connection weights between distance and readout cells could be formed developmentally by Hebbian learning during the propagation of a wave of activity along the distance cell population, while the readout cell firing rate increased gradually over time. This process need only occur once, as the same connectivity is then utilized across all environments.

The vector cell models require fewer additional neurons, as the need for an intermediate representation of absolute location is eliminated. This also allows the spatial resolution of vector cells to be reduced for greater encoded displacements, with translation vectors being dynamically recalculated as the goal is approached (see Supplemental Experimental Procedures; Figures S3 and S4). Vector cells would fire whenever an animal planned to navigate to any one of a band of goal locations at a fixed distance from the current location along a specific 1D axis. This response would be invariant to translation of the current and goal locations, in contrast to the purely allocentric co-ordinate frame utilized by distance cells. Synaptic connectivity between pairs of grid cells in each module and vector cells could be formed developmentally by Hebbian learning during the coordinated propagation of two parallel waves of activity across the sheet of grid cells in each module, where the separation between the waves reflects a specific phase difference along a 1D axis and the corresponding set of vector cells remain active. Again, we note that this process need only occur once. In the case of the rate-coded vector cell model, those synaptic connections must also be multiplicative, which lacks biological plausibility (but see Mel, 1993), although the same functionality could be achieved by the integration of inputs on distinct dendritic branches (London and Häusser, 2005). The phase-coded vector cell model avoids the need for multiplicative synapses but requires grid cells that exhibit phase precession aligned with specific 1D axes, and it is unclear from current data whether such temporal coding exists within grid cells of the mEC (Hafting et al., 2008; Reifenstein et al., 2012; Climer et al., 2013; Jeewajee et al., 2014).

Unlike direct decoding models, the linear look-ahead model makes use of neural mechanisms that are already in place to update grid cell activity according to self-motion. In continuous attractor network models of grid cell firing, directional input from conjunctive cells in deeper layers could drive grid cell activity during linear look ahead (Fuhs and Touretzky, 2006; Sargolini et al., 2006; Burak and Fiete, 2009); whereas in oscillatory interference models this input would come from velocity controlled oscillators (VCOs; Burgess et al., 2007; Burgess, 2008; Hasselmo, 2008; Welday et al., 2011). Independent of either model, the necessary synaptic connectivity could also develop through temporally asymmetric Hebbian learning within networks of conjunctive grid by head-direction cells in the deeper layers of mEC that would allow linear look ahead along the preferred firing direction of those cells (Sargolini et al., 2006; Kubie and Fenton, 2012).

Each of the direct decoding models predicts activity in grid cells encoding goal locations during route planning. It has been demonstrated that place cells in humans are reactivated during the retrieval of an episodic memory associated with that location (Miller et al., 2013), but whether similar reactivation occurs during route planning or in grid cells has yet to be determined. Importantly, the phase-coded vector cell model also predicts that the relative timing of this activity in grid cells across modules would correspond to their theta firing phase at the current location. Conversely, the linear look-ahead model predicts the sequential activation of bands of grid cells that share a common phase on a specific 1D axis during route planning, analogous to place cell replay and preplay events (Foster and Wilson, 2006; Davidson et al., 2009; Pfeiffer and Foster, 2013; see S.G. Trettel and L.L. Colgin, 2014, Soc. Neurosci., abstract for similar activity in grid cells during sleep). Interestingly, recent data indicates that place cell ripple related preplay can include novel routes (Ólafsdóttir et al., 2015)—a key property of vector navigation. However, the linear look-ahead model proposes activity sweeps along two non-collinear axes, not necessarily oriented toward the goal, in contrast to reports of goal-directed preplay (Pfeiffer and Foster, 2013).

## Discussion

We have described an algorithmic solution to the computational problem of large-scale vector navigation with grid cells. That is, how to accurately compute translation vectors between arbitrary locations in large-scale 2D space using the grid cell representations of those locations. This problem is the inverse of that thought to be performed by grid cells during path integration—extracting the translation vector between current and goal locations, as opposed to combining a previously known location with a subsequent movement vector to estimate the current location. Specifically, we have shown how the spatial phases of activity in grid cell modules of different spatial scales at start and goal locations can be used to extract the distance and direction between those locations. This is achieved by finding the maximum slope of a plane that fits the family of points defined by the phase difference in each grid module and the inverse scale of that module on at least two non-collinear axes (Figure 4C). Importantly, this solution is robust to differences in grid orientation between grid modules and ellipticity (i.e., differences in scale between axes) within each grid module (Stensola et al., 2012). This solution relates to the Fourier shift theorem, whereby the 2D translation applied to a basis set of Fourier components can be recovered from the phase changes across components (Orchard et al., 2013).

We have also described several neural network implementations of this algorithmic solution, building on a large body of previous work that has explored how grid cells efficiently encode location (Fiete et al., 2008; Mathis et al., 2012) and might contribute to vector navigation (Huhn et al., 2009; Masson and Girard, 2011; Erdem and Hasselmo, 2012; Kubie and Fenton, 2012; Erdem and Hasselmo, 2014). These models assume only that the grid representations of current and goal locations are known and produce direct vectors between those locations that may traverse previously unknown terrain. Each proposed implementation can decode 2D translation vectors with an accuracy and range that is comparable to the theoretical capacity of the grid cell system, and each model offers specific strengths, weaknesses, and experimental predictions. Several computational implementations that make use of the Chinese Remainder Theorem to perform this conversion have previously been proposed (Sun and Yao, 1994; Fiete et al., 2008; Masson and Girard, 2011). These models have limitations, however, such as requiring a hard-wired energy landscape or readout weights, producing linear outputs that are only correct modulo the lowest common multiple of grid scales or performing gradient descent on an energy landscape with multiple local minima (Masson and Girard, 2011).

Two critical considerations for all grid cell models of vector navigation are whether grid cells provide a single, global representation of large-scale space and how vector navigation might be affected by local distortions of that representation. Several studies have demonstrated that grid firing patterns in isolated environments can become distorted or fragmented by local boundaries (Barry et al., 2007; Derdikman et al., 2009; Krupic et al., 2015; Stensola et al., 2015). These deformations will impair the ability of grid firing patterns to support vector navigation unless they affect all grid cell modules equally, which is not clear from current data (but see Stensola et al., 2012). Interestingly, however, it has recently been shown that grid firing patterns in two separate environments are initially local but become globally consistent when the animal is allowed sufficient experience of navigating between environments (Carpenter et al., 2015). Hence, given the opportunity to learn the relative location of different local environments within a larger space, grid cell firing patterns could provide a universal spatial metric for vector navigation across large distances.

It is also important to note that the vector navigation models described here cannot function in isolation. Grid cell firing patterns must be anchored to environmental sensory stimuli, both to prevent noise-related drift in the grid cell representation of space and to facilitate the subsequent planning and execution of real behavioral trajectories, which would incorporate the sensory and affective features of locations lying along the decoded vector. This sensory input might be mediated, in part, by projections from place, head direction, and boundary vector cells. Moreover, the allocentric translation vectors extracted from the grid cell network by each of the models presented here would generally need to be converted into egocentric movement strategies elsewhere in the brain before they could be utilized for actual navigation (Byrne et al., 2007).

Similarly, we note that both band cells (Burgess et al., 2007; Krupic et al., 2012; Mhatre et al., 2012) and velocity-controlled oscillators (VCOs), postulated by the oscillatory interference model (Burgess et al., 2007; Burgess, 2008; Hasselmo, 2008) and identified in the hippocampal formation (Welday et al., 2011), encode periodic spatial phase with a constant scale along a specific 1D axis. Such firing patterns correspond to Fourier components of a 2D spatial representation (Orchard et al., 2013) and could therefore be used in place of grid cell inputs to support both the algorithmic solutions and various neural network implementations presented here. Moreover, VCOs show the appropriate frequency dependence on velocity to encode displacement along a specific direction in their firing phase relative to the baseline (LFP) oscillation and subsequently support the phase-coded vector cell model. It is possible, therefore, that band cells or VCOs perform path integration and support vector navigation, while grid cells represent the interface between those cells and sensory information encoded by place cells (e.g., O’Keefe and Burgess, 2005; Bush et al., 2014). Further experiments are required to determine whether band cells or VCOs exist with the discrete range of spatial scales that would be required to support vector navigation over large distances.

To conclude, we have provided a theoretical framework within which to examine the computational problem of large-scale vector navigation using grid cells and presented an algorithmic solution to the problem and several biologically plausible implementations of that solution. Although the system we have described is focused on navigation, the same procedure could be applied to compute the displacement between any arbitrary pair of positions in any physical or conceptual space and in any number of dimensions. Future experiments must determine whether mEC is needed for vector navigation and, if so, what neurophysiological signatures it is associated with.

## Author Contributions

D.B. conceived the distance cell model and implemented all models; D.M. conceived the rate-coded vector cell model; C.B. conceived the phase-coded vector cell model; N.B. conceived the algorithmic solution; C.B. and N.B. instigated the research; and D.B., C.B., D.M., and N.B. wrote the manuscript.

## Supplemental Information

Document S1. Supplemental Experimental Procedures and Figures S1–S5

Document S2. Article plus Supplemental Information
