# Supplementary material for: Using Grid Cells for Navigation
Source: Neuron. 2015 Aug 5;87(3):507–20. doi: 10.1016/j.neuron.2015.07.006 (PMC4534384; doi:10.1016/j.neuron.2015.07.006)
Supplement: Document S1. Supplemental Experimental Procedures and Figures S1–S5 [file mmc1.pdf]

Neuron

Supplemental Information

Using Grid Cells for Navigation

Daniel Bush, Caswell Barry, Daniel Manson, and Neil Burgess

Supplemental Data

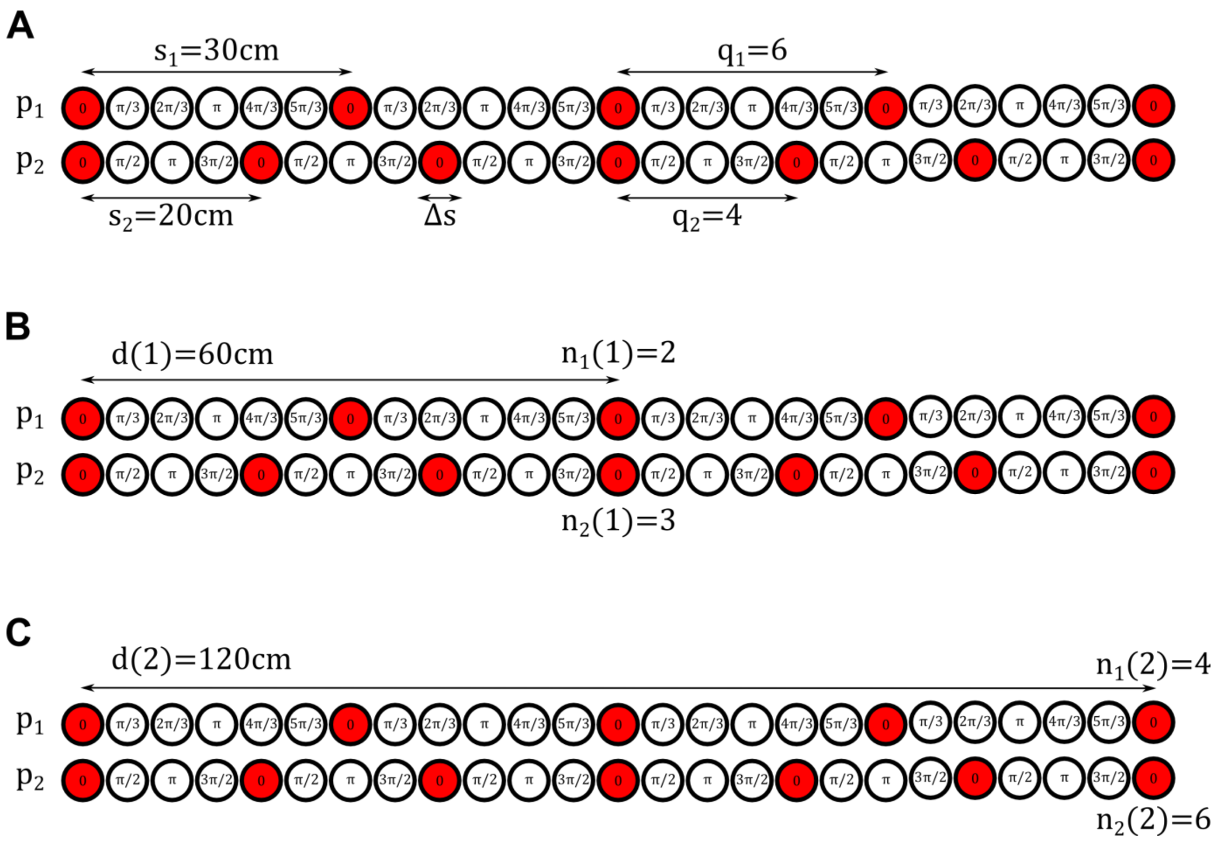

Figure S1: Capacity of the Grid Cell System (see also Algorithmic Solution in 1D, Supplemental Experimental Procedures). The displacement d of a location encoded by a specific combination of grid cell phases p_i across modules must satisfy the equation $d(k) = s_i(p_i/2\pi + n_i(k))$ for all $i = 1$ to M (Equation S1), where s_i is the scale of module i and n_i an integer value for that module. There are multiple solutions to this equation, indexed here by k, such that specific combinations of grid cell phases across modules correspond to multiple, periodically spaced locations along the axis. The capacity of the grid cell system is defined as the distance between consecutive solutions $d(k+1) - d(k)$ (Equation S2). If we express each grid scale s_i as an integer multiple q_i of the distance resolution of coding across modules Δs (i.e. $s_i = q_i \Delta s$, but see Supplemental Experimental Procedures), then we can show that the distance between successive locations $d(k+1) - d(k)$ is equal to the lowest common multiple of $\{q_i\}$ multiplied by the distance resolution Δs (Equation S5). In the example shown here, $M = 2$ grid cell modules with a distance resolution $\Delta s = 5cm$ have scales of $s_1 = 30cm$ and $s_2 = 20cm$ (i.e. $q_1 = 6$ and $q_2 = 4$). When both grid modules have a phase of $p_i = 0\,rad$, **(A)** the first solution to Equation S1 is $d(0) = 0cm$, given by $n_1 = 0$ and $n_2 = 0$; **(B)** the second solution is $d(1) = 60cm$, given by $n_1 = 2$ and $n_2 = 3$; and **(C)** the third solution is $d(2) = 120cm$, given by $n_1 = 4$ and $n_2 = 6$. Hence, the capacity of the grid cell system $d(k+1) - d(k) = 60cm$ which is equal to the lowest common multiple of $q_1 = 6$ and $q_2 = 4$, i.e. 12, multiplied by the distance resolution $\Delta s = 5cm$.

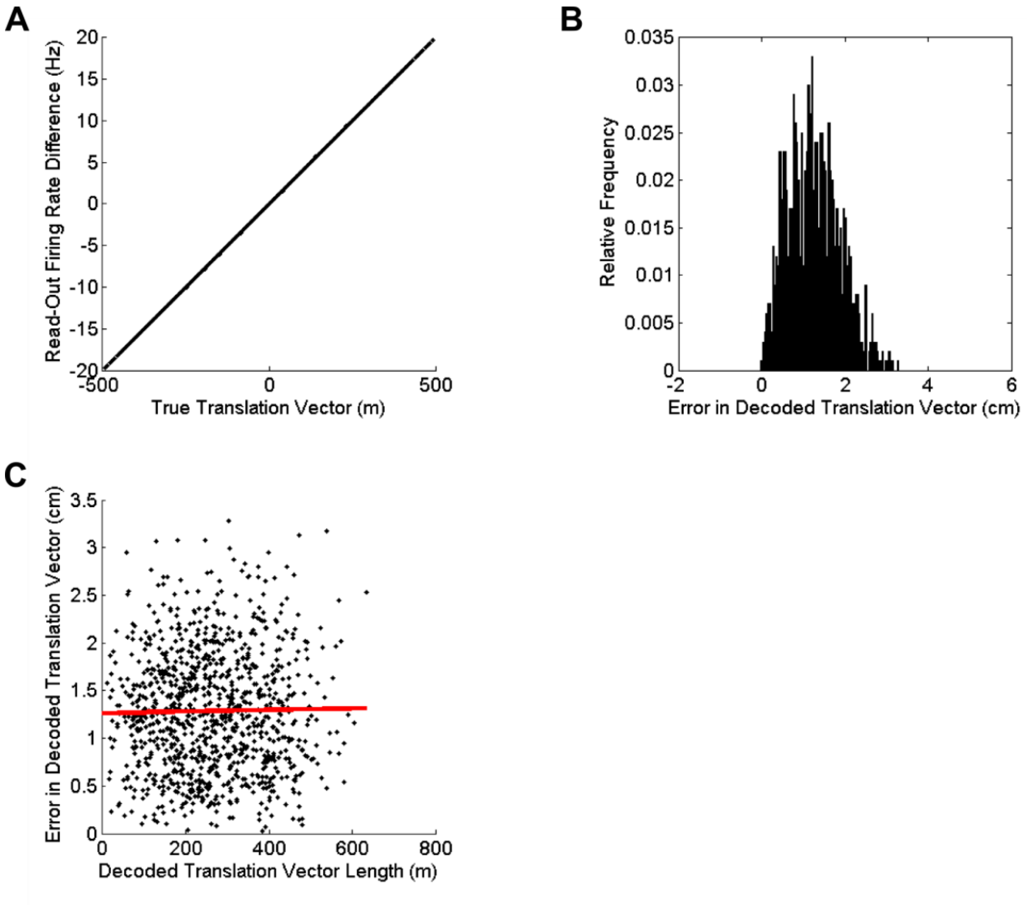

Figure S2: Simulations of the Distance Cell Model (see also Figure 5, Supplemental Experimental Procedures). We postulate two arrays of N_{DC}=12500 distance cells that encode start and goal locations, respectively, on each of two axes $\vec{x}$ and $\vec{y}$. Grid cells in each module project to distance cells with synaptic weights that are proportional to their mean firing rate at the corresponding location on that axis (Figure 5A). The number of spikes fired by N_{GC}=400 grid cells, representing m_i=20 equally distributed spatial phases p_i along each axis, is given by a Poisson process with a rate function that is cosine tuned for location along that axis (Equation S6) and a time window of 100ms. Distance cells in each array are subject to winner-take-all dynamics, and all distance cells in each array project to a pair of read-out cells with synaptic weights that increase topographically in different directions along each axis (Figure 5B). Hence, the relative firing rate of those read-out cells encodes the displacement between start and goal locations along that axis. **(A)** The difference in read-out cell firing rates (collapsed across both principal axes) plotted against the true translation vector for N=1000 simulations with randomly assigned start and goal locations in a 500m sided 2D arena (see Experimental Procedures). This illustrates that translation vectors can be accurately decoded from the relative firing rate of read-out cells. **(B)** The distribution of errors in 2D translation vector lengths decoded from the relative firing rate of read-out cells across the same N=1000 simulations. Note that distance cells were given a resolution of 4cm in these simulations. **(C)** Total decoded 2D translation vector lengths plotted against decoding error. This demonstrates that there is no correlation (r=0.017, p=0.60) between the length of translation vectors and the decoding error, as the spatial resolution of distance cells is even across the entire 2D arena.

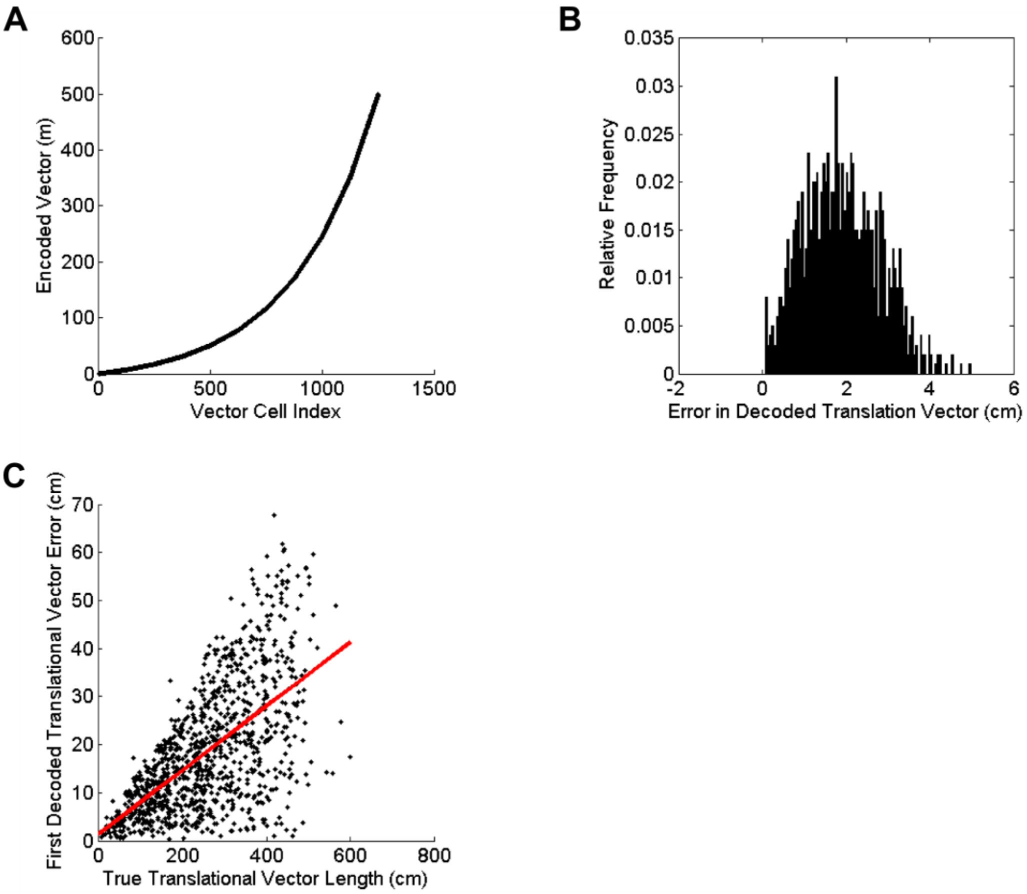

Figure S3: Simulations of the Rate-coded Vector Cell Model (see also Figure 6, Supplemental Experimental Procedures). We postulate two arrays of N_{VC}=1250 vector cells that encode the displacement between start and goal locations in either direction along each of two axes $\vec{x}$ and $\vec{y}$. Vector cells receive input from grid cell pairs within each module whose unwrapped phase difference corresponds to that absolute vector on that axis through multiplicative synapses (Figure 6A). The number of spikes fired by N_{GC}=400 grid cells, representing m_i=20 equally distributed spatial phases p_i along each axis, is given by a Poisson process with a rate function that is cosine tuned for location along that axis (Equation S6) and a time window of 100ms. Vector cells in each array are subject to winner-take-all dynamics, and translation vectors are directly decoded from the weighted mean of vector cell activity. Translation vectors are calculated iteratively, with the start location updated by 80% of the total decoded vector length along each axis prior to each new calculation, until the start location is within 1m of the true goal. **(A)** Pseudo-exponential distribution of translation vectors encoded by vector cells. **(B)** The distribution of translation vector errors decoded in the final iterative step from the activity of vector cells across N=1000 simulations with randomly assigned start and goal locations in a 500m sided 2D arena (see Experimental Procedures). **(C)** Total decoded 2D translation vector lengths plotted against decoding error in the first iterative step. This demonstrates that there is a significant correlation ($r=0.61$, $p<0.001$) between the length of translation vectors and initial decoding error, as the spatial resolution of vector cells decreases with increasing encoded displacement (Figure S3A).

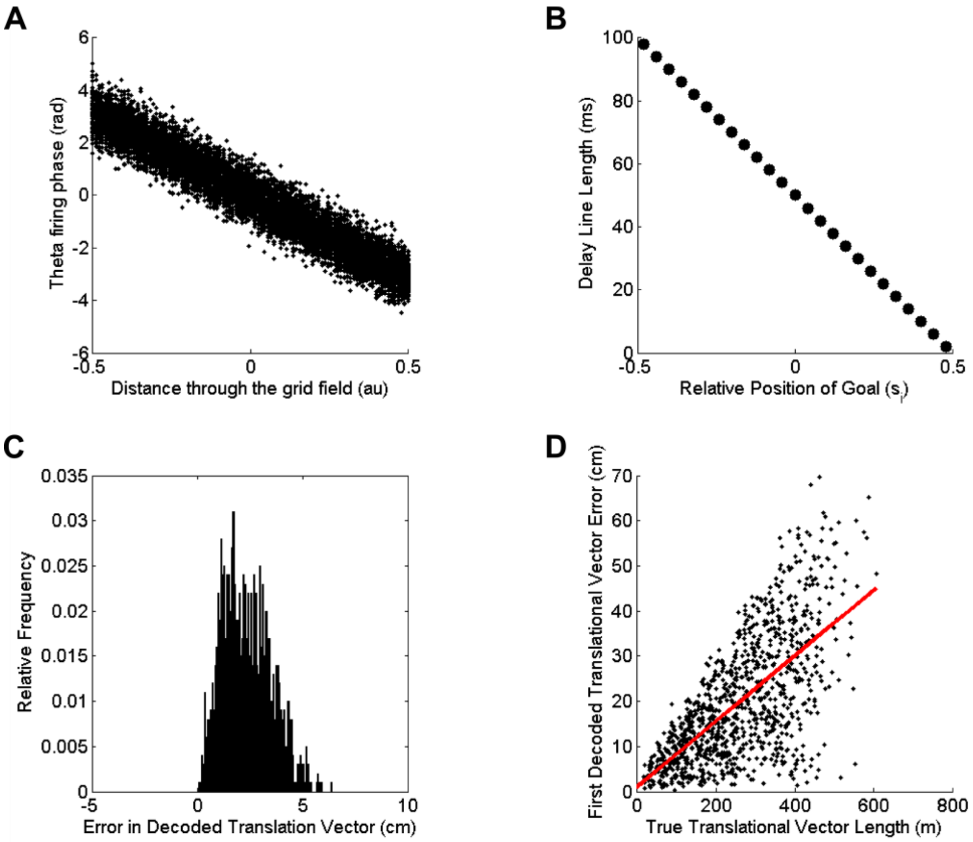

Figure S4: Simulations of the Phase-coded Vector Cell Model (see also Figure 7, Supplemental Experimental Procedures). We postulate two populations of grid cells that exhibit phase precession aligned with specific non-collinear axes (Figure 7A, B); and that only the grid cells within each module which exhibit the maximum rate at the goal location are reactivated, firing one spike within a single t_{theta}=100ms at a theta phase consistent with the current location. **(A)** Theta firing phases are drawn from a circular normal distribution with a mean that is linearly correlated with the distance (modulo grid scale) between the peak of that grid cell's firing field and the current location along the axis of phase precession (Equation S7) and a circular standard deviation of $\mu = \pi/6$ rad (compare to Figure 7A). **(B)** Grid cells project to vector cells through delay lines with transmission delays which ensure that spikes from grid cells encoding the goal location across modules arrive simultaneously at the corresponding vector cell (Equation S8). The activity of vector cells is determined by the temporal proximity of incoming spikes, i.e. vector cells perform temporal coincidence detection on the inputs that arrive from grid cells through delay lines. Vector cells in each array are subject to winner-take-all dynamics, and translation vectors can be directly decoded from the weighted mean of vector cell activity. Translation vectors are calculated iteratively, with the start location updated by 80% of the total decoded vector length along each axis prior to each new calculation, until the start location is within 1m of the true goal. **(C)** The distribution of translation vector errors decoded in the final iterative step from the activity of vector cells across *N*=1000 simulations with randomly assigned start and goal locations in a 500m sided 2D arena (see Experimental Procedures). **(D)** Total decoded 2D vector lengths plotted against decoding error in the first iterative step. This demonstrates that there is a significant correlation (r=0.66, p<0.001) between the length of translation vectors and initial decoding error, as the spatial resolution of vector cells decreases with increasing encoded displacement (Figure S3A).

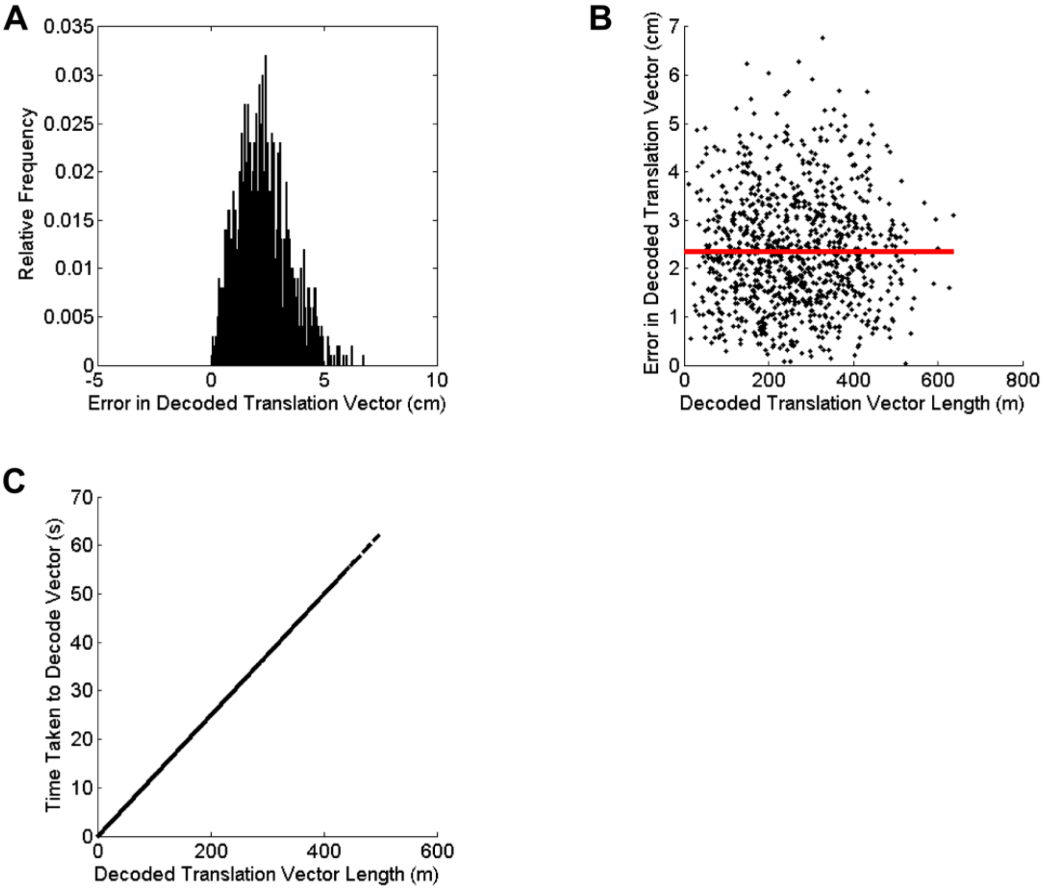

Figure S5: Simulations of the Linear Look-ahead Model (see also Figure 8, Supplemental Experimental Procedures). Linear look ahead activity is initiated by setting grid cell firing rates in each module to match those at the start location and then updating those firing rates in each subsequent 5ms time step according to simulated movement away from the current location at a constant speed of $8ms^{-1}$ along a specific directional axis. Grid cells in each module project to an array of place cells that evenly cover the 2D arena with synaptic weights that are proportional to their mean firing rate at the centre of the place field, and place cells are subject to winner-take-all dynamics. The time taken for grid cells encoding the goal location across modules to become simultaneously active, or the firing rate of a neuron that integrates total activity in the grid cell network during linear look ahead, subsequently encodes the displacement between start and goal locations along that axis (Figure 8A). This is indicated by activity in the corresponding place cell. The number of spikes fired by N_{GC}=400 grid cells, representing m_i=20 equally distributed spatial phases p_i along each axis, is given by a Poisson process with a rate function that is cosine tuned for location along that axis (Equation S6). Overall translation vectors in 2D are constructed from linear look ahead in each direction along two non-collinear axes. **(A)** The distribution of decoding errors across N=1000 simulations with randomly assigned start and goal locations in a 500m sided 2D arena. **(B)** Total decoded 2D translation vector lengths plotted against decoding error. This demonstrates that there is no correlation (r=0.017, p=0.60) between the length of translation vectors and the decoding error, as place cell firing fields evenly cover the entire 2D arena. **(C)** The time taken to decode translation vectors along each axis (i.e. the length of each linear look ahead event) is determined by the displacement between start and goal locations along that axis.

Supplemental Experimental Procedures

Capacity of the Grid Cell System

We can calculate the capacity of the grid cell system within our formalism (following Fiete et al., 2008). If we consider multiple distinct solutions $d(k)$ that satisfy Equations 1 and 2 for different sets of integer values $n_i(k)$, where $i = 1$ to M, then from Equation 1 (or Figure 4A) we have:

$$d(k) = s_i(p_i/2\pi + n_i(k)) \qquad \text{for all } i = 1 \text{ to } M$$

[Equation S1]

The distance between adjacent solutions is:

$$d(k+1) - d(k) = s_i(n_i(k+1) - n_i(k)) \qquad \text{for all } i = 1 \text{ to } M$$

[Equation S2]

If we express s_i in integer multiples $\{q_i\}$ of the distance resolution of coding within each grid module (i.e. the minimum distinguishable distance, Δs, which we assume to be constant across modules, but see below), so that $s_i = q_i \Delta s$, then:

$$\frac{(d(k+1) - d(k))}{\Delta s} = (n_i(k+1) - n_i(k))q_i \quad \text{for all } i = 1 \text{ to } M$$

[Equation S3]

If there are no common factors in $\{q_i\}$, where $i = 1$ to M, then Equation S3 requires that:

$$\big(n_i(k+1) - n_i(k)\big) = \prod_j q_j \qquad \text{for } j = 1 \text{ to } M, j \neq i$$

[Equation S4]

so that:

$$d(k+1) - d(k) = \Delta s \prod_j q_j \qquad \text{for } j = 1 \text{ to } M$$

[Equation S5]

Thus the separation between consecutive ambiguous solutions is the product of the grid scales in units of the minimum distinguishable distance in a grid module (unless there are common factors in $\{q_i\}$, in which case the separation is equal to the least common multiple of grid scales; see Fiete et al., 2008), and displacements within this range are encoded unambiguously. As a consequence, grid scales should avoid common factors, or the separation between ambiguous solutions will be correspondingly reduced (for further discussion, see Mathis et al., 2012, 2013; Towse et al., 2014). However, a fixed distance resolution across modules seems unlikely, assuming that neurons in different modules have common biophysical properties and given the apparently greater number of grid cells with small rather than large scales (Stensola et al., 2012). A more realistic assumption would be a fixed phase resolution across modules (i.e. distance resolution is proportional to grid scale, $\Delta s_i \propto s_i$), in which case Fiete et al. (2008) show that capacity has a similar exponential dependence on the number of modules *M*.

Neural Network Simulations

In all simulations, locations are encoded by M=10 grid cell modules whose spatial scales are arranged in a geometric progression (i.e. $s_i = s_M \alpha^{M-i}$) with a minimum grid scale of s_{10}=25cm and a common factor of α=1.4 (giving a maximum grid scale of ~5m). Each module consists of N_{GC}=400 grid cells distributed evenly among m_i=20 equally distributed spatial phases p_i. We note that the exact distribution of grid scales has no effect on the simulations presented here, aside from determining the capacity of the grid cell system to encode unique locations (Fiete et al., 2008; Mathis et al., 2012; Wei et al., 2013; Towse et al., 2014; see 'Capacity of the Grid Cell System' above) and the ability of the grid cell system to deal with neural noise (Sreenivasan and Fiete, 2011; Mathis et al., 2013).

For each model, translation vectors are decoded for N=1000 randomly assigned current and goal locations in a 2D arena. These locations are constructed from randomly assigned displacements along two 500m principal axes of that arena. For simplicity, these axes are oriented at $\vec{x} = 0°$ and $\vec{y} = 60°$ to match the principal axes of the grid pattern. We note that the size of this arena is significantly less than the capacity of this grid cell network to encode locations with a unique set of grid cell phases across modules. Using the formalism above and a distance resolution across modules of $\Delta s = 0.4m$, which represents the maximum accuracy with which distances can be resolved from noisy grid cell firing in the largest grid module alone, we estimate a capacity of ~3.3km for the grid cell system examined here.

In all rate-coded simulations, the firing of grid cells is modelled as a Poisson process with a rate function $r_{j,i}$ that exhibits cosine tuning for location a in module i along each principal axis u_j and a maximum rate r_{max}=30Hz (Equation S6):

$$r_{j,i}(a) = r_{max} \frac{1 + \cos\left(\left[\frac{a - s_i\left(\frac{p_{j,i}}{2\pi}\right)}{s_i}\right] \times 2\pi\right)}{2}$$

[Equation S6]

All Matlab code used to perform these simulations is available on ModelDB (http://modeldb.yale.edu/182685).

The Distance Cell Model

In distance cell simulations, distance cells encode current or goal locations in allocentric space along the principal axes $\vec{x}$ and $\vec{y}$ (Fiete et al., 2008; Huhn et al., 2009). Two distance cell arrays (one for each of the principal axes $\vec{x}$ and $\vec{y}$) receive input from a population of grid cells whose firing rates encode the current location, and two separate distance cell arrays (one for each of the principal axes $\vec{x}$ and $\vec{y}$) receive input from a population of grid cells whose firing rates encode the goal location. It has been suggested that distance cells may correspond to place cells in the dentate gyrus (DG; Huhn et al., 2009), which significantly outnumber those in the CA3 and CA1 subfields (~1x10^6 granule cells in rat DG and ~2.5x10^5 pyramidal cells in rat CA3 or CA1; Amaral and Lavenex, 2007) and whose properties have not been well characterised (Jung and McNaughton, 1993). However, distance cells in this model exhibit a band-like firing pattern, as they encode a series of allocentric locations along a specific 1D axis with a spatial periodicity equal to the capacity of the grid cell system.

Grid cells project to distance cells in each array with synaptic weights that are proportional to their mean firing rate at the allocentric location a encoded by that distance cell on the directional axis $\vec{x}$ or $\vec{y}$, i.e. $w_{DC,GC} \propto r_{j,i}(a)$ (Figure 5A; Equation S6), analogous to models of the grid cell to place cell transformation (Rolls et al., 2006; Solstad et al., 2006). The number of spikes fired by each grid cell is dictated by a Poisson process with a cosine tuned rate function (Equation S6) and a time window of 100ms.

Distance cells have a spatial resolution of 0.04m to give N_{DC}=12500 distance cells in each of the four arrays (corresponding to current and goal locations on the two axes $\vec{x}$ and $\vec{y}$), or a total of 50000 distance cells overall. Distance cells within each array are subject to winner-take-all dynamics implemented with an E%-max algorithm, such that cells only fire if their input is within k=1% of the maximum feed-forward excitation (de Almeida et al., 2009). Activity within each distance cell array is then normalised, such that the integrated activity across all distance cells within each 100ms time window is consistent across simulations.

Synaptic weights between each pair of (current and goal location) distance cell arrays and two read-out neurons for each principal axis $\vec{x}$ or $\vec{y}$ increase linearly in opposing directions along topographically ordered distance cells (Figure 5B). Hence, the relative firing rate of these two read-out neurons encodes the displacement between current and goal locations on that axis. The magnitude of translation vectors on each principal axis is decoded by fitting a line of regression to a plot of the difference in the firing rate of read-out cells against the true displacement vector along that axis across all N=1000 iterations (Fig S2A). We note that these putative read-out cells require both a very large and highly precise range of firing rates to represent planned trajectories, and this function might more easily be supported by populations of read-out cells.

Simulations demonstrate that the distance cell model can decode translation vectors between arbitrary current and goal locations in large-scale 2D space within a 100ms time window with a mean accuracy of <4cm (Fig S2B). Importantly, there is no correlation between the magnitude of the vector and the error with which it is decoded (r=0.017, p=0.60, Fig S2C), as distance cells evenly cover the arena.

The Vector Cell Models

In vector cell simulations, separate populations of vector cells decode the displacement between arbitrary start and goal locations in each direction along the principal axes $\vec{x}$ and $\vec{y}$. The spatial resolution of vector cells is pseudo-exponentially distributed in the 0 : 500m range (Fig S3A) to give a total of N_{VC}=1250 vector cells in each of four arrays (corresponding to each direction of movement along the two principal axes $\vec{x}$ and $\vec{y}$), or a total of 5000 vector cells overall. This is an order of magnitude smaller than the additional number or cells required by the distance cell model (see above), as the need for an intermediate representation of absolute location is eliminated. In turn, this allows the spatial resolution of vector cells to be reduced for greater encoded displacements, as translation vectors can be dynamically recalculated with increasing accuracy as the goal is approached (see also Erdem and Hasselmo, 2014). We note that this does not reduce the capacity of the grid cell system, it merely allows that capacity to be under-sampled at greater distances from the current location.

In these simulations, for each pair of N=1000 arbitrary start and goal locations, the current location is updated by 80% of the decoded vector length along each axis in each step, and then translation vectors are iteratively re-calculated, until the current location is within 1m of the true goal. In each step, vector cells within each pair of arrays encoding displacements along a single axis are subject to winner-take-all dynamics implemented with an E%-max algorithm, such that cells only fire if their input is within k=1% of the maximum feed-forward excitation (de Almeida et al., 2009). The overall translation vector is decoded by combining the average of all active vector cells along each axis $\vec{x}$ or $\vec{y}$, weighted by their firing rate.

In rate-coded vector cell simulations, each pair of vector cell arrays that encode displacement in either direction along the principal axis $\vec{x}$ or $\vec{y}$ receive input from a population of grid cells that encodes start and goal locations on that axis. Vector cells receive input from grid cell pairs within each module whose unwrapped phase difference corresponds to that absolute vector on that directional axis through multiplicative synapses, such that activity only reaches the vector cell if both grid cells in a start-goal pair are active (Figure 6A). The number of spikes fired by each grid cell in each step is dictated by a Poisson process with a cosine tuned rate function (Equation S6) and a time window of 100ms.

Simulations demonstrate that the rate-coded vector cell model can decode translation vectors between arbitrary current and goal locations in large-scale 2D space with a mean accuracy of <4cm (Fig S3B). These translation vectors are decoded rapidly, in the minimum possible number of iterative 100ms steps in all simulations (mean = 3.83 steps, range of 2-4 steps). The magnitude of error in the translation vector decoded in the first step correlates with the length of that vector (r=0.61, p<0.001; Fig S3C), due to the decrease in vector cell precision for greater vector lengths, while no such relationship is observed for the distance cell model (Fig S2C).

In phase-coded vector cell simulations, pairs of vector cell arrays that encode displacement in either direction along the principal axis $\vec{x}$ or $\vec{y}$ receive input from separate populations of grid cells that exhibit phase precession aligned with each axis. Only the grid cells within each module GC_b that exhibit the maximum firing rate at the goal location b fire within a single t_{theta}=100ms theta cycle. Their theta firing phase is drawn from a circular normal distribution with a mean $\overline{\emptyset_\iota}$ that is linearly correlated with the circular distance between the peak of that grid cell's firing field b and the current location a along that axis (Equation S7) and a circular standard deviation of $\mu = {}^{\pi}/_{6}$ rad (Figure S4A).

$$\overline{\emptyset_\iota}(GC_b) = 2\pi \frac{mod(b - a, s_i)}{s_i}$$

[Equation S7]

In these simulations, vector cells must be sensitive to specific spike timing input patterns, analogous to models of polychronous computation (Jeffress, 1948; Hopfield, 1995; Izhikevich, 2006). For simplicity, this is achieved by grid cells in each module projecting to vector cells through delay lines with transmission delays t_i that are proportional to the difference between the grid scale s_i and the encoded vector d according to Equation S8 (Figure S4B). The activity of each vector cell is then taken as the resultant vector length of input spike phases from grid cells encoding the goal location in all modules.

$$t_i(d) = \frac{mod(\frac{s_i}{2} - d, s_i)}{s_i} t_{theta}$$

[Equation S8]

Simulations demonstrate that the phase-coded vector cell model can decode translation vectors between arbitrary current and goal locations in large-scale 2D space with an accuracy of <4cm (Fig S4C). These translation vectors are decoded rapidly, in the minimum number of iterative 100ms steps in all simulations (mean = 3.82 steps, range of 2-4 steps). Again, the magnitude of error correlates with the size of decoded vectors (r=0.66, p<0.001; Fig S4D), due to the decrease in vector cell precision for greater vector lengths, while no such relationship is observed for the distance cell model (Fig S2C).

We note that there is little empirical evidence for the existence of delay line connectivity of the kind employed here in cortex (but see McKenzie et al., 2014). However, the same functionality could be achieved by dendritic integration that allows the sequence and relative timing of inputs to be discriminated (Branco et al., 2010; Vaidya and Johnston, 2013). Interestingly, differences in theta phase and ionic conductances along the dendrite could affect the integration of inputs at the soma to make hippocampal pyramidal neurons sensitive to the relative timing and distribution of their inputs (Bullock et al., 1990; Vaidya and Johnston, 2013). It is important to note that this model is insensitive to the actual underlying oscillatory frequency against which phase precession occurs, so that a more realistic time constant of dendritic integration (i.e. <10ms) could be used if phase precession transiently occurred against a higher frequency oscillation. Moreover, the model does not rely on linear phase precession, or on phase precession covering the full range of 2π radians - any constant, monotonic relationship between position and theta firing phase is sufficient to support the decoding of translation vectors.

One experimental prediction of the phase-coded vector cell model is the existence of separate populations of grid cells that exhibit phase precession along specific 1D axes. Phase precession has been best characterised for non-directional grid cells and corresponds to distance travelled through the firing field irrespective of direction (Hafting et al., 2008; Reifenstein et al., 2012; Climer et al., 2013; Jeewajee et al., 2014). However, a subpopulation of conjunctive head-direction modulated grid cells in the deeper layers of mEC do show reliable theta phase precession (Hafting et al., 2008; Newman and Hasselmo, 2014; Reifenstein et al., 2014) which might serve this purpose (but see Climer et al., 2013).

The Linear Look Ahead Model

In the linear look ahead model, four linear look ahead events are required to compute translation vectors between arbitrary start and goal locations in 2D space, corresponding to the sequential exploration of each direction along the two principal axes $\vec{x}$ and $\vec{y}$. During each linear look ahead event, activity is initiated by setting grid cell firing rates in each module to match those at the start location and then updating grid cell firing in each subsequent time step according to simulated movement away from the current location at a constant speed along that directional axis.

The distance between start and goal locations in either direction along each principal axis is decoded from the time elapsed between the initiation of linear look ahead activity and firing activity in the

place cell encoding the goal location. This could be encoded by the firing rate of a cell that integrates total activity in a single grid cell module, or the grid cell system as a whole, over the course of that linear look ahead event (for a similar proposal during active navigation, see Kubie and Fenton, 2009). Some layer II mEC cells are known to exhibit bistable persistent spiking, such that their firing rate reflects a stable integral of the depolarisation reaching the cell (Klink and Alonso, 1997; Egorov et al., 2002; Fransen et al., 2006).

Overall translation vectors in 2D space are constructed by combining the decoded distances and direction along each of the principal axes. We note that the principal axes along which linear look ahead is performed need not be consistent between events, nor must the same axes be explored in either direction – any set of non-collinear, directional axes that are sufficient to triangulate an arbitrary location in 2D space will suffice. Moreover, as linear look ahead events are carried out sequentially, these could correspond to successive head directions of the animal during scanning behaviour.

In these simulations, we set a time step *dt*=5ms that corresponds to the integration time constant of a post-synaptic neuron – that is, grid cells across modules that fire within a 5ms time window encode a single location that can be decoded by an output place cell. We then set a constant, virtual speed for linear look ahead events of v_{sweep}=8ms^{-1} (Davidson et al., 2009), which subsequently determines the distance increment between each integration time step and therefore the spatial resolution of output place cells (0.04m in this case). Synaptic weights between grid cells and place cells are proportional to the grid cell firing rate at the peak of the place cell firing field along the axis $\vec{x}$ or $\vec{y}$, i.e. $w_{DC,GC} \propto r_{j,i}(a)$ (Equation S6). The number of spikes fired by each grid cell in each time step is dictated by a Poisson process with a cosine tuned rate function (Equation S6) and the time window of *dt*=5ms. Activity within each place cell array is subject to winner-take-all dynamics implemented with an E%-max algorithm such that cells only fire if their input is within *k*=1% of the maximum feed-forward excitation (de Almeida et al., 2009).

Simulations demonstrate that the linear look ahead model can decode translation vectors between arbitrary current and goal locations in 2D space with an accuracy of <4cm (Fig S5A). Like the distance cell model, there is no correlation between the magnitude of the vector and the error with which it is decoded (r=0.017, p=0.60, Fig S5B), as the firing fields of output place cells evenly cover the arena. However, the time taken to decode translation vectors is correlated with their magnitude, and can be an order of magnitude larger than the direct decoding models (Fig S5C). For example, linear look ahead at a constant speed of v_{sweep}=8ms^{-1} takes approximately one minute to decode a single 500m vector, and four of these linear look ahead events, corresponding to independent searches in each direction along two non-collinear axes, are required to compute a 2D translation vector. It is possible to increase the speed of linear look ahead activity, but at the cost of precision, as a broader population of grid cells fire within each integration time window. Moreover, the capacity of the grid cell system will be reduced if smaller scales can no longer contribute to encoding distinct locations, as will occur if all grid cells in those modules fire within the time window of post-synaptic integration. Similarly, if the distance to the goal is not known a priori, then the speed of linear look ahead activity cannot be tailored to match the scale of the decoded vector (but see Erdem and Hasselmo, 2014).

We note that linear look ahead activity in the grid cell network is phenomenologically similar to hippocampal replay and preplay events, during which trajectories often originate close to the animal and travel coherently through space (Foster and Wilson, 2006; Davidson et al,. 2009; Pfeiffer and Foster, 2013). However, preplay trajectories in place cells also appear to move directly towards future goals (Pfeiffer and Foster, 2013; Olafsdottir et al., 2015), which requires a priori knowledge of the direction towards those locations. Hence, it is possible that the planning of a complete navigational trajectory using linear look ahead might take place in two stages. First, linear look ahead in grid cells could be used to compute a direct vector to the goal location. During this period, place cell read-out activity would be driven by grid cell firing and used to ascertain the point at which grid cells encoding the goal location across modules became simultaneously active. Next, preplay activity in place cells could be used to sequentially check the sensory attributes of each location along that direct vector, in order to identify any potential hazards or impediments to navigation. During this period, place cell preplay activity would resemble a trajectory moving directly away from the current location towards, and terminating at, the goal (Pfeiffer and Foster, 2013; Olafsdottir et al., 2015). We note that this latter stage could also be used to simulate the sensory content of planned trajectories after a translation vector was computed from grid cell representations using any of the direct decoding models, and would be consistent with experimental indications that sharp wave/ripple (SWR) dynamics originate in the recurrent collaterals of CA3 (Chrobak and Buzsaki, 1996; Sullivan et al., 2011).

Supplemental References

Amaral D, Lavenex P (2007) Hippocampal Neuroanatomy. The Hippocampus Book, Oxford University Press, Oxford, UK

Branco T, Clark BA, Häusser M (2010) Dendritic discrimination of temporal input sequences in cortical neurons. Science 329: 1671-1675

Bullock TH, Buzsáki G, McClune MC (1990) Coherence of compound field potentials reveals discontinuities in the CA1-subiculum of the hippocampus in freely-moving rats. Neuroscience 38: 609-619

Chrobak JJ, Buzsáki G (1996) High-frequency oscillations in the output networks of the hippocampal-entorhinal axis of the freely behaving rat. Journal of Neuroscience 16: 3056-3066

de Almeida L, Idiart M, Lisman JE (2009) A second function of gamma frequency oscillations: an E%-max winner-take-all mechanism selects which cells fire. Journal of Neuroscience 29: 7497-7503

Egorov AV, Hamam BN, Fransén E, Hasselmo ME, Alonso AA (2002) Graded persistent activity in entorhinal cortex neurons. Nature 420: 173-178

Fransen E, Tahvildari B, Egorov AV, Hasselmo ME, Alonso AA (2006) Mechanism of graded persistent cellular activity of entorhinal cortex layer V pyramidal neurons. Neuron 49: 735-746

Hopfield JJ (1995) Pattern recognition computation using action potential timing for stimulus representation. Nature 376: 33-36

Izhikevich EM (2006) Polychronisation: Computation With Spikes. Neural Computation 18: 245-282

Jeffress LA (1948) A Place Theory of Sound Localisation. Journal of Comparative and Physiological Psychology 41: 35-39

Jung MW, McNaughton BL (1993) Spatial selectivity of unit activity in the hippocampal granular layer. Hippocampus 3: 165-182

Klink R, Alonso A (1997) Muscarinic modulation of the oscillatory and repetitive firing properties of entorhinal cortex layer II neurons. Journal of Neurophysiology 77: 1813-1828

Kubie JL, Fenton AA (2009) Heading-vector navigation based on head-direction cells and path integration. Hippocampus 19: 456-479

McKenzie IA, Ohayon D, Li H, de Faria JP, Emery B, Tohyama K, Richardson WD (2014) Motor skill learning requires active central myelination. Science 346: 318-322

Newman EL, Hasselmo ME (2014) Grid cell firing properties vary as a function of theta phase locking preferences in the rat medial entorhinal cortex. Frontiers in Systems Neuroscience 8: 193

Sullivan D, Csicsvari J, Mizuseki K, Montgomery S, Diba K, Buzsáki G (2011) Relationships between hippocampal sharp waves, ripples, and fast gamma oscillation: influence of dentate and entorhinal cortical activity. Journal of Neuroscience 31: 8605-8616

Towse BW, Barry C, Bush D, Burgess N (2014) Optimal configurations of spatial scale for grid cell firing under noise and uncertainty. Philosophical Transactions of the Royal Society B: Biological Sciences 369: 20130290

Vaidya SP, Johnston D (2013) Temporal synchrony and gamma-to-theta power conversion in the dendrites of CA1 pyramidal neurons. Nature Neuroscience 16: 1812-1820

Wei XX, Prentice J, Balasubramanian V (2013) The sense of place: grid cells in the brain and the transcendental number e. arXiv: 1304.0031
