# Supplementary material for: Using Grid Cells for Navigation
Source: Neuron. 2015 Aug 5;87(3):507–20. doi: 10.1016/j.neuron.2015.07.006 (PMC4534384; doi:10.1016/j.neuron.2015.07.006)
Supplement: Document S2. Article plus Supplemental Information [file mmc2.pdf]

Viewpoint

Neuron

Using Grid Cells for Navigation

Highlights

- Grid cells (GCs) are believed to provide a path integration input to place cells
- However, GCs also provide a powerful context-independent metric for large-scale space
- Hence, we show how GCs can be used for vector navigation between arbitrary locations
- We simulate various neural implementations and make testable experimental predictions

Authors

Daniel Bush, Caswell Barry, Daniel Manson, Neil Burgess

Correspondence

drdanielbush@gmail.com (D.B.),
n.burgess@ucl.ac.uk (N.B.)

In Brief

Grid cells are thought to support path integration, but also provide a context-independent metric for large-scale space. Here, Bush et al. show how grid cells could be used for vector navigation and explore the predictions of several potential neural implementations.

CrossMark

Bush et al., 2015, Neuron *87*, 507–520
August 5, 2015 ©2015 The Authors
http://dx.doi.org/10.1016/j.neuron.2015.07.006

CellPress

CellPress

Using Grid Cells for Navigation

Daniel Bush,[1,2,5,*] Caswell Barry,[3,5] Daniel Manson,[3,4] and Neil Burgess[1,2,*]
[1]UCL Institute of Cognitive Neuroscience, 17 Queen Square, London, WC1N 3AR, UK
[2]UCL Institute of Neurology, Queen Square, London, WC1N 3BG, UK
[3]UCL Department of Cell and Developmental Biology, Gower Street, London, WC1E 6BT, UK
[4]UCL Centre for Mathematics and Physics in the Life Sciences and Experimental Biology, Gower Street, London, WC1E 6BT, UK
[5]Co-first author
*Correspondence: drdanielbush@gmail.com (D.B.), n.burgess@ucl.ac.uk (N.B.)
http://dx.doi.org/10.1016/j.neuron.2015.07.006
This is an open access article under the CC BY license (http://creativecommons.org/licenses/by/4.0/).

SUMMARY

Mammals are able to navigate to hidden goal locations by direct routes that may traverse previously unvisited terrain. Empirical evidence suggests that this "vector navigation" relies on an internal representation of space provided by the hippocampal formation. The periodic spatial firing patterns of grid cells in the hippocampal formation offer a compact combinatorial code for location within large-scale space. Here, we consider the computational problem of how to determine the vector between start and goal locations encoded by the firing of grid cells when this vector may be much longer than the largest grid scale. First, we present an algorithmic solution to the problem, inspired by the Fourier shift theorem. Second, we describe several potential neural network implementations of this solution that combine efficiency of search and biological plausibility. Finally, we discuss the empirical predictions of these implementations and their relationship to the anatomy and electrophysiology of the hippocampal formation.

INTRODUCTION

It is believed that mammals can use an internal representation of space to navigate directly to goal locations (O'Keefe and Nadel, 1978; Gallistel, 1990) without following explicit sensory cues (Morris et al., 1982) or a well-learned sequence of actions (Packard and McGaugh, 1996). This "vector navigation" problem can be posed in terms of how the representation of a goal location can be combined with that of the current location to infer the vector between the two. Importantly, the resulting trajectory may be novel, having never before been taken by the animal, and could pass through regions of the environment that have not previously been visited (Tolman, 1948). Moreover, this ability does not require learning from reinforcement over multiple trials (e.g., Sutton and Barto, 1998) as it can occur within a single trial (Steele and Morris, 1999), benefit from "latent" learning in the absence of reinforcement (Tolman, 1948; Bendig, 1952; Keith and McVety, 1988), and need not show blocking or overshadowing between multiple cues (Hayward et al., 2003; Doeller and Burgess, 2008).

The ability to perform vector navigation is impaired by bilateral damage to the hippocampal formation (Morris et al., 1982; Parron and Save, 2004; Steffenach et al., 2005; Van Cauter et al., 2013). Similarly, metabolic activity in the human hippocampus correlates with navigational performance (Maguire et al., 1998; Hartley et al., 2003; Iaria et al., 2003), and damage to the hippocampus is associated with impaired spatial navigation (Kolb and Whishaw, 1996; Abrahams et al., 1997; Burgess et al., 2002) in addition to more general mnemonic deficits (Scoville and Milner, 1957; Squire and Zola-Morgan, 1991; Cohen and Eichenbaum, 1993). At the neural level, the mammalian hippocampal formation contains several different representations of self-location and orientation including place cells in the hippocampus proper (O'Keefe and Dostrovsky, 1971; Muller and Kubie, 1987); head direction cells in the subicular complex and deeper layers of mEC (J.B. Ranck, 1984, Soc. Neurosci., abstract; Taube et al., 1990; Sargolini et al., 2006); and grid cells in the superficial layers of mEC, pre- and para-subiculum (Hafting et al., 2005; Sargolini et al., 2006; Boccara et al., 2010). Earlier models of vector navigation generally focused on the well-characterized spatial activity of place cells (e.g., Dayan, 1991; Burgess et al., 1994; Sharp et al., 1996; Touretzky and Redish, 1996; Conklin and Eliasmith, 2005). In smaller environments, place cells typically exhibit a single spatial receptive field, firing whenever the animal enters a specific portion of the environment. As such, a simple way to navigate using place cells is to compare a representation of the goal location with that of the current location and move so as to increase the similarity between the two (Burgess and O'Keefe, 1996).

However, despite providing a potentially useful one-to-one relationship with the locations of specific sensory and affective environmental features, place cell firing patterns do not explicitly represent the structure of space (O'Keefe and Nadel, 1978). There appears to be no consistent relationship between the locations of a place cell's firing fields in different environments (O'Keefe and Conway, 1978; Thompson and Best, 1989) and no pattern relating the multiple firing fields that a place cell may have in larger environments (Fenton et al., 2008). These properties imply that any mapping between place cell representations and translation vectors used for navigation would have to be re-learned in each new environment. Moreover, navigation

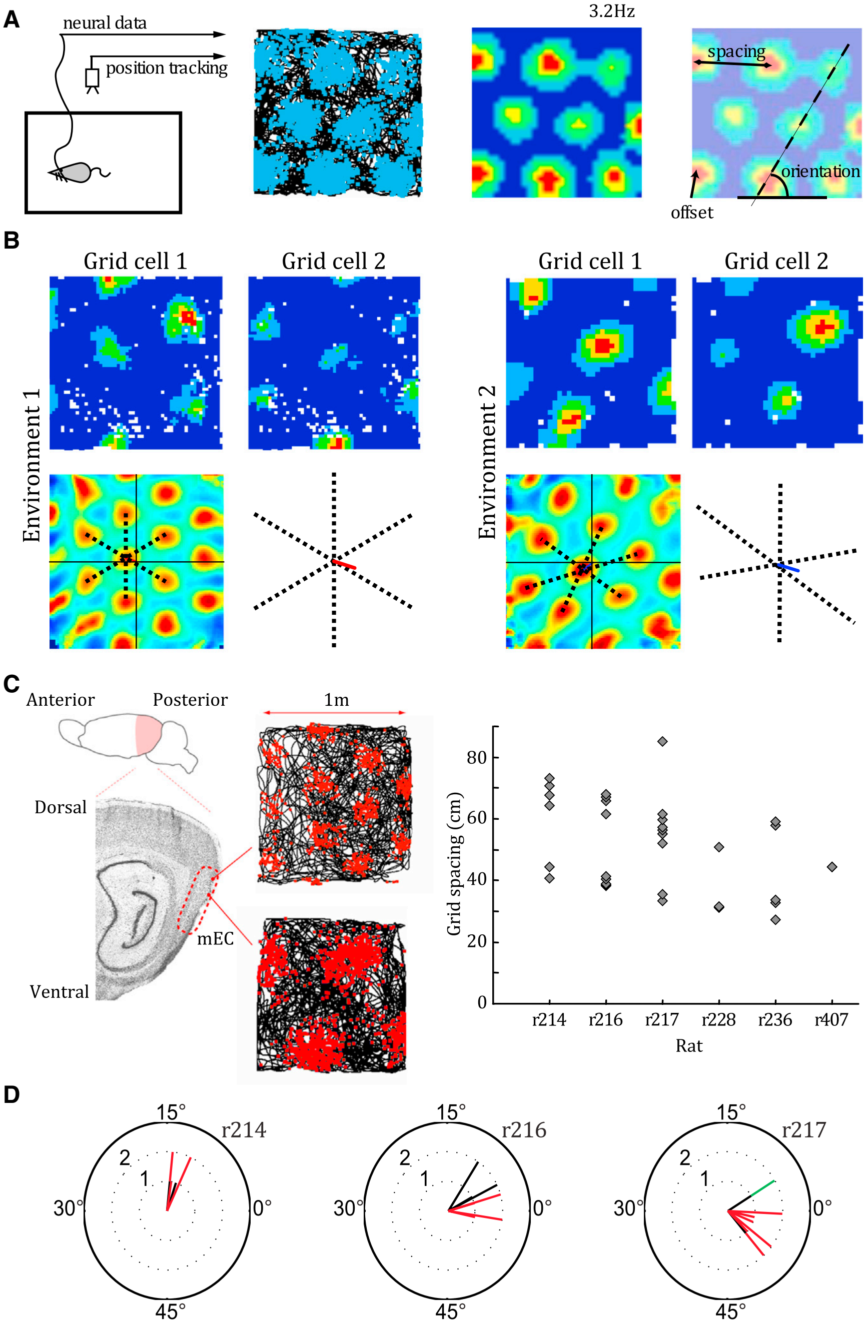

Figure 1. Properties of the Grid Cell System

(A) Left: schematic of single unit recording. Middle left: raw data from a sample mEC grid cell. The animal's path is indicated by the black line, and the positions at which action potentials were fired are superimposed in blue. Middle right: firing rate map for the same mEC grid cell, with high firing rates indicated by "hot" colors. Right: the regular grid-like firing pattern can be characterized by its orientation, scale, and offset or spatial "phase."

(B) Two mEC grid cells co-recorded on a single tetrode in different environments exhibit the same grid scale and orientation but differ in their offset or relative spatial phase. Top row: firing rate maps for a pair of grid cells recorded in a familiar (left) and novel (right) environment. Bottom row: spatial cross-correlation of the grid cell firing rate maps in each environment. Black dashed lines indicate the central six peaks of the cross-correlation; colored line shows the distance and direction from the central peak to the origin of the spatial cross-correlation, after correcting for changes in grid scale and ellipticity. This illustrates that the offset between the firing fields of those two grid cells is preserved between environments, even when the grid pattern has expanded and deformed (adapted from Barry et al., 2012).

(C) Grid cells appear to be organized into discrete functional modules whose scale increases in discrete steps along the dorso-ventral axis of mEC (adapted from Barry et al., 2007).

(D) Grid field orientation of grid cells recorded in three different rats. The orientations of grid firing patterns are significantly clustered within and between modules. Grid cells with spatial scales that differ by less than 20% are assumed to belong to a single module and grouped by color (adapted from Barry et al., 2007).

using place cell representations is limited in range to the diameter of the largest place fields, unless combined with experience-dependent learning over multiple trials (e.g., Dayan 1991; Blum and Abbott, 1996; Brown and Sharp, 1995; Foster et al., 2000), which will tend to bias behavior toward previously learned routes. Beyond this range, the similarity of the current and goal place cell representations will be zero, providing no gradient in similarity leading to the goal location. Although large place fields have been recorded (~10 m; Kjelstrup et al., 2008), these properties clearly limit the utility of place cell representations for large-scale vector navigation.

In contrast to place cells, grid cells exhibit several properties that afford large-scale vector navigation. Grid cells also show stable spatial firing correlates but with multiple firing fields distributed in a regular hexagonal array that covers all environments visited by the animal (Hafting et al., 2005; Sargolini et al., 2006; Figure 1A). Grid cells are organized into functional modules within medial entorhinal cortex (mEC): cells that are proximate in the brain tend to have firing patterns that share the same scale and orientation but a fixed spatial offset relative to one another (i.e., exhibit a different spatial "phase"; Hafting et al., 2005; Barry et al., 2007; Stensola et al., 2012). Importantly, the relative spatial phase of any two simultaneously recorded grid cells from the same module appears to be conserved across all environments visited by the animal, and a small number of grid firing patterns can completely cover the environment (Hafting et al., 2005; Sargolini et al., 2006; Figure 1B). Grid scale increases between modules in discontinuous steps along the dorso-ventral axis of mEC, with the smallest

being around 25 cm and the largest so far recorded exceeding 300 cm and probably representing the fourth or fifth of up to ten discrete scales (Barry et al., 2007; Stensola et al., 2012; Figure 1C). The orientations of grid firing patterns in different modules are also clustered (Barry et al., 2007; Stensola et al., 2012; Figure 1D). It is not yet clear whether grid cells in the pre- and para-subiculum have the same topography (Boccara et al., 2010).

The regular periodic firing patterns of grid cells potentially provide a compact code for location that resembles a residue number system, encoding positions over a very large range that approaches the lowest common multiple of the spatial scales of all grid modules (Gorchetchnikov and Grossberg, 2007; Fiete et al., 2008; Sreenivasan and Fiete, 2011; Mathis et al., 2012). Interestingly, grid cells are widely believed to provide a path integration input to place cells, updating the representation of self-location by a vector describing the animal's recent motion (Hafting et al., 2005; O'Keefe and Burgess, 2005; McNaughton et al., 2006; Rolls et al., 2006; Solstad et al., 2006). However, by providing a context-independent spatial metric, grid cells also have the potential to solve the inverse problem of vector navigation—to compute a translation vector between current and previously known locations, as opposed to combining a previously known location with the subsequent movement vector to compute the current location. More generally, the periodic firing patterns of grid cells appear to provide a framework with which to infer the vector between two locations, even when those locations are much farther apart than the largest grid scale (Gorchetchnikov and Grossberg, 2007; Fiete et al., 2008; Huhn et al., 2009; Masson and Girard, 2011; Erdem and Hasselmo, 2012; Kubie and Fenton, 2012).

Here, we consider the problem of large-scale vector navigation with grid cells at Marr's three levels of analysis (Marr and Poggio, 1977). First, we outline the computational problem to be solved: how to compute a translation vector between coordinates encoded in an idealized grid cell system, and describe how this relates to the capacity of that system to encode unique locations (see also Gorchetchnikov and Grossberg, 2007; Fiete et al., 2008; Sreenivasan and Fiete, 2011). Second, we describe an algorithmic solution to this problem, based on the grid cell network and inspired by the Fourier shift theorem (see also Orchard et al., 2013). This solution is focused on resolving ambiguity between the multiple, periodic locations represented by activity within each grid module, rather than optimizing the efficiency and accuracy of the grid cell code for location within the scale of the largest grid (for this latter topic, see Mathis et al., 2012, 2013; Wei et al., 2013). Finally, we describe several plausible neural network implementations that use grid cells to calculate the translation vector between start and goal locations in 2D space over distances that can exceed the largest grid scale (see also Fiete et al., 2008; Huhn et al., 2009; Erdem and Hasselmo, 2012; Kubie and Fenton, 2012; Erdem and Hasselmo, 2014). We focus on proposed mechanisms that can perform vector navigation relatively rapidly (i.e., without an exhaustive search of the numerous possible solutions) and that provide experimentally testable predictions.

RESULTS

The Computational Problem

The Grid Cell Representation of Space

We parameterize the grid cell spatial representation as follows: there are M grid cell "modules" with spatial scale s_i (s_1 being the largest and s_M the smallest) that each consist of a topographically ordered population of m_i cells. In 1D space, we can visualize each module of grid cells as a ring that supports a population activity bump centered at phase p_i where $0 \le p_i < 2\pi$ (Figure 2A). In 2D space, we can visualize each module of grid cells as a twisted torus supporting a single activity bump centered at phases $\overrightarrow{p_i} = (p_{x,i},\ p_{y,i})$ along the principal axes of a unitary "tile" of the grid pattern (i.e., unit vectors $\overrightarrow{x}$ and $\overrightarrow{y}$; see Figures 2B and 2C; Guanella et al., 2007). Note that one can choose any two non-collinear axes to define the grid phase and corresponding unit tile (Kubie and Fenton, 2012) but, for simplicity, we consider two of the axes of symmetry of the grid pattern so that grid scale is equal on each. Moreover, twisted torus connectivity is only necessary when considering grid cell activity as a single bump—other network topologies can account for the grid cell firing pattern when multiple activity bumps are present (e.g., Fuhs and Touretzky, 2006; Burak and Fiete, 2009). Finally, increasing the number of grid cells within a module improves precision, but not the amount of information encoded beyond the two degrees of freedom needed to define the animal's location within the corresponding tile.

The Vector Navigation Problem

The 1D vector navigation problem can be stated thus: given the grid cell representations of two locations a and b, calculate the displacement between those locations $d = b - a$ (Figure 3A). More specifically, the grid cell representations of locations a and b correspond to the spatial phases of activity bumps in each grid module $\{p_i(a) \mid i = 1\ to\ M\} = \{p_1(a), p_2(a), \ldots, p_M(a)\}$ and $\{p_i(b) \mid i = 1\ to\ M\} = \{p_1(b), p_2(b), \ldots, p_M(b)\}$. As an example, consider three grid cell modules with scales $s_1 = 50\ cm$, $s_2 = 30\ cm$ and $s_3 = 20\ cm$. If the distance between the current location a and goal location b is $d = 75\ cm$, and (for the sake of simplicity, but without loss of generalization) the phase of each module is 0 at the current location a—i.e., $p_1(a) = 0$, $p_2(a) = 0$ and $p_3(a) = 0$—then at b the phase of each module will be proportional to the distance d modulo grid scale s_i:

$$p_1(b) = \frac{75\ mod\ 50}{50} \times 2\pi = \pi$$

$$p_2(b) = \frac{75\ mod\ 30}{30} \times 2\pi = \pi$$

$$p_3(b) = \frac{75\ mod\ 20}{20} \times 2\pi = \frac{3\pi}{2}.$$

The 1D vector navigation problem is to recover the displacement d from these values of $\{p_i(b)\}$ (Figure 3A).

Similarly, the 2D vector navigation problem can be stated thus: given the grid cell representations of two locations $\overrightarrow{a}$ and $\overrightarrow{b}$, calculate the displacement vector between those locations $\overrightarrow{d} = \overrightarrow{b} - \overrightarrow{a}$. More specifically, the grid cell

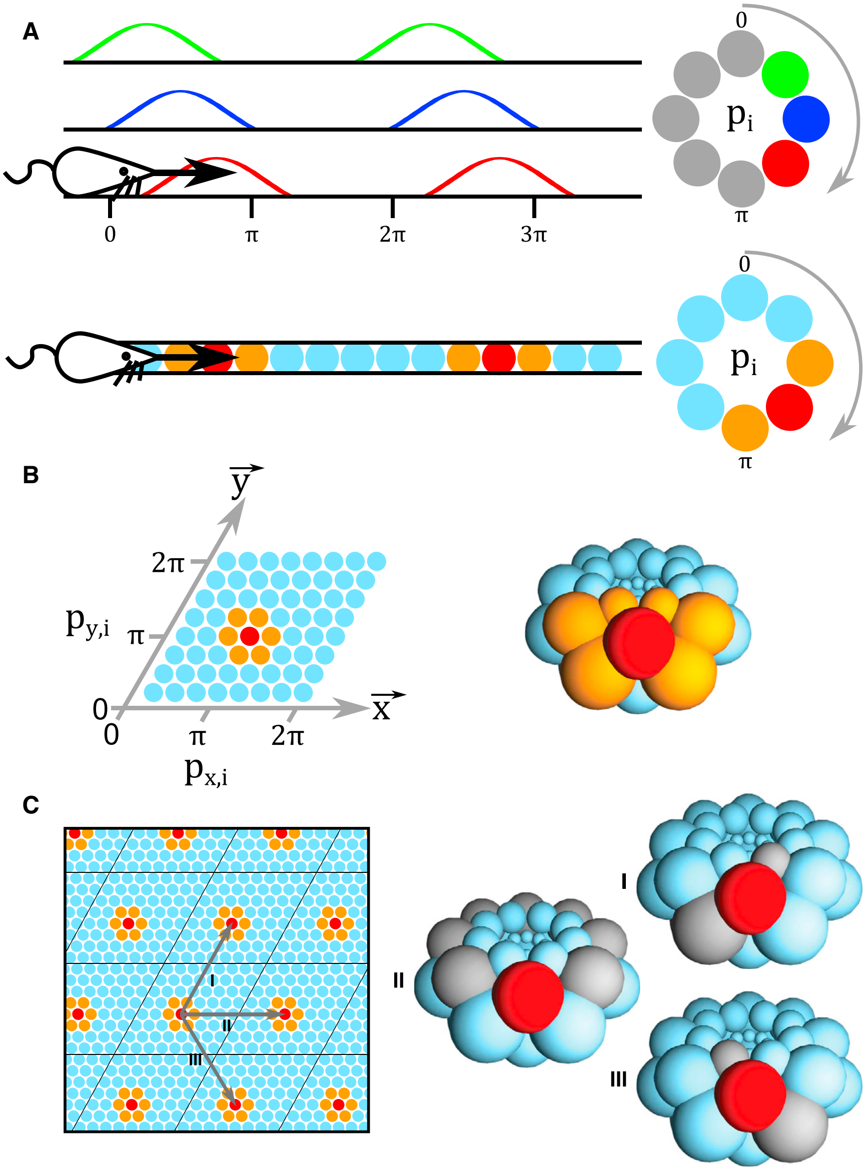

Figure 2. The Grid Cell Representation of Space

(A) Top: in 1D, a single module of grid cells encode location with spatially offset, periodic firing fields corresponding to different phases of activity p_i in a ring of cells. Bottom: the position of an animal can therefore be described by the periodic spatial phase p_i that corresponds to a single activity bump (hot colors) moving around the ring of cells according to the animal's self-motion.

(B) In 2D, a single module i of grid cells encodes the location of an animal as a pair of spatial phases $\vec{p}_i = (p_{x,i}, p_{y,i})$ along the grid axes $\vec{x}$ and $\vec{y}$ (left). These axes define a single, rhombic grid cell tile that can be joined along all edges to create a twisted torus topology (right).

(C) Movement along each of the principal axes of the grid field in space (left) corresponds to movement around each of the principal axes of the twisted torus (right) in the grid cell module.

representations of locations a and b correspond to the sets of spatial phases $\{p_{x,i}(\vec{a})\} = \{p_{x,1}(\vec{a}), p_{x,2}(\vec{a}), \ldots, p_{x,M}(\vec{a})\}$ and $\{p_{y,i}(\vec{a})\} = \{p_{y,1}(\vec{a}), p_{y,2}(\vec{a}), \ldots, p_{y,M}(\vec{a})\}$ that define position $\vec{a}$ in module i along principal axes x and y (which, in this case, are separated by 60°; see Figures 2B and 2C). Again, consider three grid cell modules with scales s_1 = 50 cm, s_2 = 30 cm and s_3 = 20 cm. If the displacement vector between the current location $\vec{a}$ and goal location $\vec{b}$ is $\vec{d} = (75\,cm, 37.5\,cm)$, and the phase of each module on each axis is 0 at the current location, then at $\vec{b}$ the phases of the modules will be:

$$\{p_{x,i}(\vec{b})\} = \left\{\pi, \pi, \frac{3\pi}{2}\right\}$$

$$\{p_{y,i}(\vec{b})\} = \left\{\frac{3\pi}{2}, \frac{\pi}{2}, \frac{7\pi}{4}\right\}.$$

The 2D vector navigation problem is to recover the displacement vector $\vec{d}$ from $\{p_{x,i}(\vec{b})\}$ and $\{p_{y,i}(\vec{b})\}$ (Figure 3B). Note that this corresponds to a simple generalization of the 1D vector navigation problem to multiple axes.

Algorithmic Solution in 1D

The cyclical nature of the grid representation within each module i is such that an activity bump at phase p_i implicitly represents an infinite set of "unwrapped" phases $p_i + 2\pi n_i$, where n_i can take any integer value, corresponding to an infinite set of distances $s_i(p_i/2\pi + n_i)$ along that 1D axis that are separated by the scale s_i of module i. Initially, we assume that all phases are zero at the current location a and the distance d to a goal location b must be inferred from the grid cell representation across modules at that location $\{p_i(b)\} = \{p_1(b), p_2(b), \ldots, p_M(b)\}$. The grid representation of the goal location b is such that there is a set of unwrapped phases (one for each module) that explicitly represent the same distance—i.e., there is a set of integers $\{n_i\}$ for which:

$$d = s_i\left(\frac{p_i}{2\pi} + n_i\right) \text{for all } i. \qquad \text{(Equation 1)}$$

Graphically, this coherent set of unwrapped phases across modules falls on a horizontal line when plotted against a y axis of represented distance (i.e., $y = d$; Figure 4A) or, equivalently, on a straight line through the origin when plotted against a y axis of phase against inverse grid scale (Equation 2; Figure 4B)—i.e., there is a set of integers $\{n_i\}$ for which:

$$p_i + 2\pi n_i = 2\pi d\left(\frac{1}{s_i}\right) \text{for all } i. \qquad \text{(Equation 2)}$$

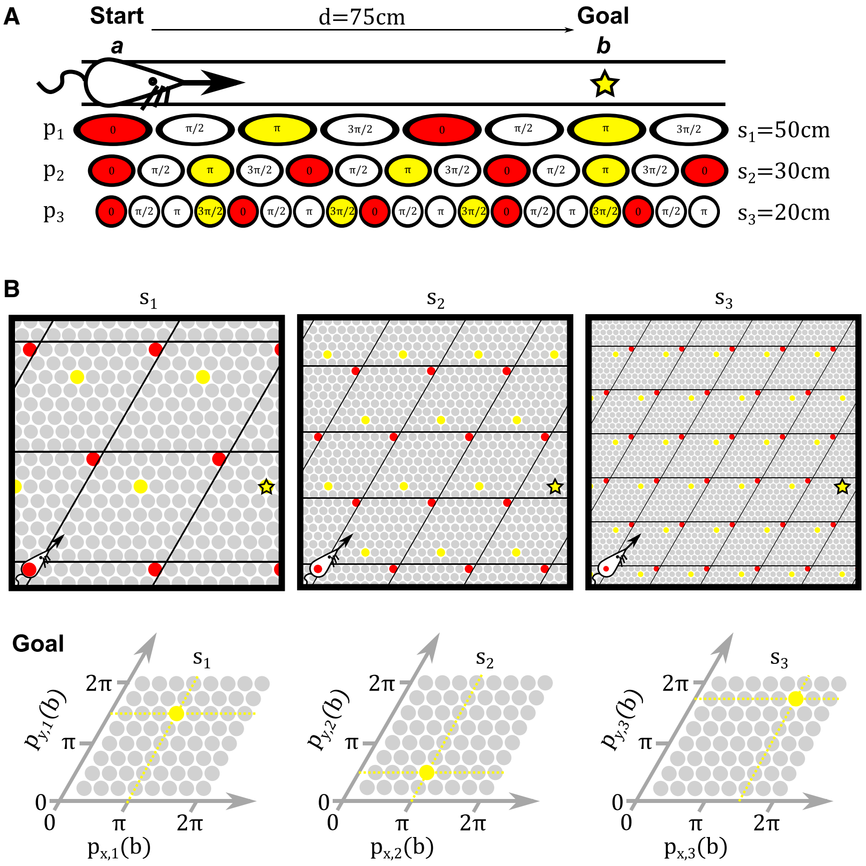

Figure 3. The Problem of Vector Navigation with Grid Cells

(A) In 1D, how do we find the displacement d between the starting location a (red) and goal location b (yellow) given the grid cell representations of those locations (i.e., sets of spatial phases across grid modules $\{p_i(a)\}$ and $\{p_i(b)\}$)?

(B) In 2D, how do we find the distance and direction between start and goal locations given the grid cell representations of those locations (i.e., sets of spatial phases across grid modules and principal axes: $\{p_x(\vec{a}), p_y(\vec{a})\}$, $\{p_x(\vec{b}), p_y(\vec{b})\}$)?

This latter relationship is obtained by re-arranging Equation 1 and corresponds to the Fourier shift theorem (see discussion of the Algorithmic Solution in 2D below; Orchard et al., 2013). Thus, the distance d to a goal location represented by the set of module phases $\{p_i\}$ can be inferred by fitting a straight line through the origin on a plot of unwrapped phases $p_i + 2\pi n_i$ against inverse grid scale $1/s_i$ across modules (Figure 4B). Moreover, this result can be generalized to any pair of arbitrary current and goal locations on that 1D axis, by replacing the absolute phase p_i at the goal location with the phase difference Δp_i between grid cell representations of current and goal locations in each module (Equation 3):

$$d = b - a = s_i\left(\frac{p_i(b)}{2\pi} + n_i(b)\right) - s_i\left(\frac{p_i(a)}{2\pi} + n_i(a)\right) \text{for all } i$$

$$\Delta p_i + 2\pi n_i = 2\pi d\left(\frac{1}{s_i}\right) \text{for all } i. \qquad \text{(Equation 3)}$$

It is important to note that Equations 1 and 2 describe an under-determined system, as there are more unknowns (M + 1, corresponding to n_i and d) than equations (M, one for each grid module). Hence, multiple possible solutions $d(k)$ exist for each unique combination of phase values $\{p_i\}$ or phase differences $\{\Delta p_i\}$ across modules, such that one set of grid cell phases across modules represents more than one, periodically spaced location in the real world (Figure S1). The capacity of the grid cell system is defined as the maximum spatial range within which each combination of phase values $\{p_i\}$ (or phase differences $\{\Delta p_i\}$) corresponds to a unique decoded location (or displacement)—i.e., the distance between locations encoded by the same set of grid cell phases or the period of the grid cell system as a whole. Theoretical studies suggest that this capacity is much greater than the typical foraging range of an animal (Gorchetchnikov and Grossberg, 2007; Fiete et al., 2008; Sreenivasan and Fiete, 2011; Mathis et al., 2012; see Supplemental Experimental Procedures). Beyond that capacity, the spatial representation provided by the grid cell network as a whole is periodic. Hence, Equations 1 and 2 only convert the spatial representation between residue-like and linear number systems within this capacity, and more generally convert between two residue-like number systems—one with a discrete set of smaller spatial scales and one with a single, much larger spatial scale.

Algorithmic Solution in 2D

In 2D, the location of an activity bump can be defined by considering any two non-collinear axes (denoted by unit vectors $\vec{x}$ and $\vec{y}$). An activity bump at phase $\vec{p_i} = (p_{x,i}, p_{y,i})$ in module i then maps onto an infinite series of periodic locations $s_i((p_{x,i}/2\pi) + n_{x,i})\vec{x} + s_i((p_{y,i}/2\pi) + n_{y,i})\vec{y}$, where $\{\vec{n_i}\} = \{n_{x,i}, n_{y,i}\}$ can be any pair of integers. We initially assume, for simplicity, that the orientations of different grid modules are aligned, i.e., $\vec{x}$ and $\vec{y}$ are independent of i (Barry et al., 2007; Stensola et al., 2012) and that grid firing fields are circularly symmetric as opposed to elliptical, i.e., the scale s_i is the same for directions $\vec{x}$ and $\vec{y}$ (but see Stensola et al., 2012). In this case, the location of an activity bump within grid module i can be visualized on a cylindrical polar plot as $(r, \theta, z) = ((1/s_i), arg(\vec{u_j}), p_{j,i} + 2\pi n_{j,i})$, where $arg(\vec{u_j})$ represents the direction of the grid axes (e.g., $\vec{u_1} = \vec{x}$ and $\vec{u_2} = \vec{y}$). Considering the phases of the goal representation along each axis, and following the logic of the 1D solution, the distance and direction to the goal location is indicated by the maximum gradient of a plane through the origin that fits the phase points $p_{j,i} + 2\pi n_{j,i}$ for all modules i and axes j and lies within the capacity of the grid cell system (Figure 4C).

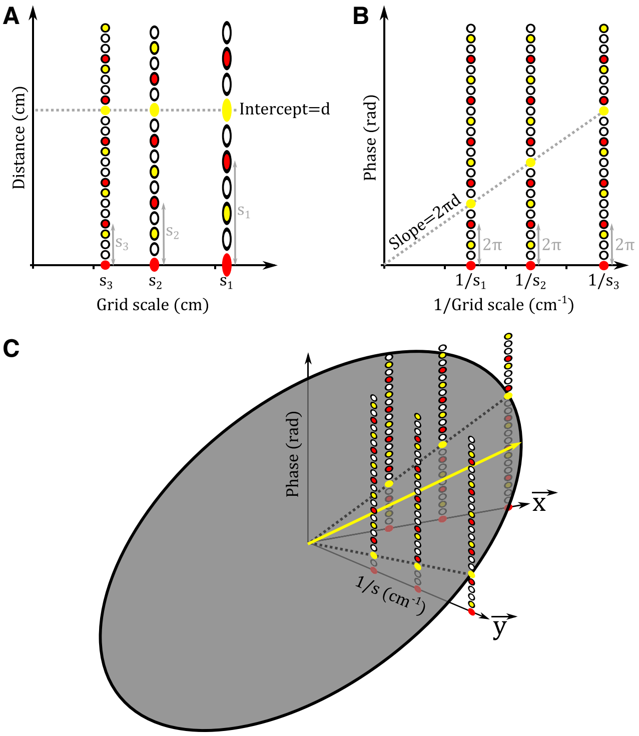

Figure 4. Algorithmic Solution to the Problem of Navigation with Grid Cells

(A) In 1D, if we assume that the phase of all grid modules at the starting location *a* is zero, then the "unwrapped" set of periodic displacements corresponding to the goal location *b*—i.e., $s_i(p_i(b)/2\pi + n_i)$, where s_i is the grid scale and n_i an integer for module *i*—fall on a horizontal line $y = d$ corresponding to the goal location (gray dashed line).

(B) Similarly, the "unwrapped" set of spatial phases across modules $p_i(b) + 2\pi n_i$, when plotted against inverse grid scale $1/s_i$, fall on a straight line through the origin with gradient $2\pi d$ (gray dashed line).

(C) In 2D, the "unwrapped" set of spatial phases corresponding to the goal location across modules $\{p_{x,i}(b) + 2\pi n_{x,i}, p_{y,i}(b) + 2\pi n_{y,i}\}$, when plotted against inverse grid scale $1/s_i$ along the principal axes $\vec{x}$ and $\vec{y}$, fall on a plane through the origin (gray ellipse) whose maximum slope (within the capacity of the grid cell system) corresponds to the distance and direction to the goal location (yellow arrow).

Again, this result can be generalized to arbitrary current and goal locations by replacing the absolute phases $p_{j,i}$ that define the goal location with the phase difference $\Delta p_{j,i}$ between the current and goal locations on each axis and in each module. This solution is consistent with the Fourier shift theorem (see Orchard et al., 2013), with the sets of grid cells in each module that share a common phase on each axis acting as Fourier components of the spatial representation. If grid cell orientations are identical across modules, then the displacement between start and goal locations can be solved independently on each axis as in the 1D case: by finding the line through the origin that best fits the phase points along that axis. We note that it is sufficient to solve for two directions, as more axes do not provide additional independent information—the constraint that $\sum_j p_{j,i} = 0$ for 3 directions separated by 120° (Burgess and Burgess, 2014) is implicitly included by fitting lines through the origin. However, given independent noise in the firing rates of biological neurons, pooling estimates across directions could potentially mitigate error in the extracted translation vector. Additionally, if grid cell orientations are not conserved across modules, or if grid firing fields are elliptical, then the solution still holds, but the plane must be fit to a family of phase points that differ in axes $\overrightarrow{u_{j,i}}$ across grid cell modules and in spatial scale s_i across axes.

Neural Network Implementations

There are many potential neural network implementations of vector navigation using grid cells, which exhibit varying degrees of efficiency, parsimony, and biological plausibility. Here, building on previous work (e.g., Sun and Yao, 1994; Gorchetchnikov and Grossberg, 2007; Fiete et al., 2008; Huhn et al., 2009; Mhatre et al., 2012; Masson and Girard, 2011; Erdem and Hasselmo, 2012; Kubie and Fenton, 2012; Erdem and Hasselmo, 2014), we describe two broad classes of solution and present neural network simulations that demonstrate the potential accuracy with which they can compute translation vectors between arbitrary locations in large-scale space (see Supplemental Information for details). The first class of solution uses additional neural circuitry to directly decode grid cell activity at current and goal locations and then read out the distance between those locations along specific 1D axes, effectively converting the grid cell residue-like number system to a linear spatial metric (see also Sun and Yao, 1994; Fiete et al., 2008; Huhn et al., 2009; Masson and Girard, 2011). The second class of solution uses network dynamics to perform sequential, directed searches along specific 1D axes, the search being initiated from either the current or goal location in order to ascertain the distance between those locations (see also Erdem and Hasselmo, 2012; Kubie and Fenton, 2012; Erdem and Hasselmo, 2014). Having described each neural network implementation, we discuss their relative strengths and weaknesses as well as the experimental predictions they make.

Vector Navigation by Direct Decoding: The "Distance Cell" Model

The "distance cell" model decodes both the absolute current and goal locations from rate-coded modular grid cell representations and then calculates the translation vector between those locations. An array of distance cells each encode a unique location *a* along a single directional axis $\vec{x}$ (see also Fiete et al., 2008; Huhn et al., 2009). Distance cells receive input from grid cells in each module with synaptic weights proportional to their mean firing rate at that location *a* on the axis $\vec{x}$. Hence, each distance cell is maximally activated by a specific set of phase values across grid cell modules $\{p_{x,i}(a)\}$ (Figure 5A), and winner-take-all dynamics within each distance cell array prevents firing in distance cells that receive lower levels of input. The total number of distance cells is limited by the capacity of the grid cell system to encode locations as unique sets of phases across grid cell modules $\{p_{x,i}(a)\}$ (see Supplemental Experimental Procedures; Figure S1), and all potential locations within that capacity are encoded by a distance cell. All distance cells provide input to a readout neuron with synaptic weights that increase in strength with increasing distance along the axis. The firing rate of this readout neuron then signals the distance from the origin to that location along the directional axis $\vec{x}$ (Figure 5A).

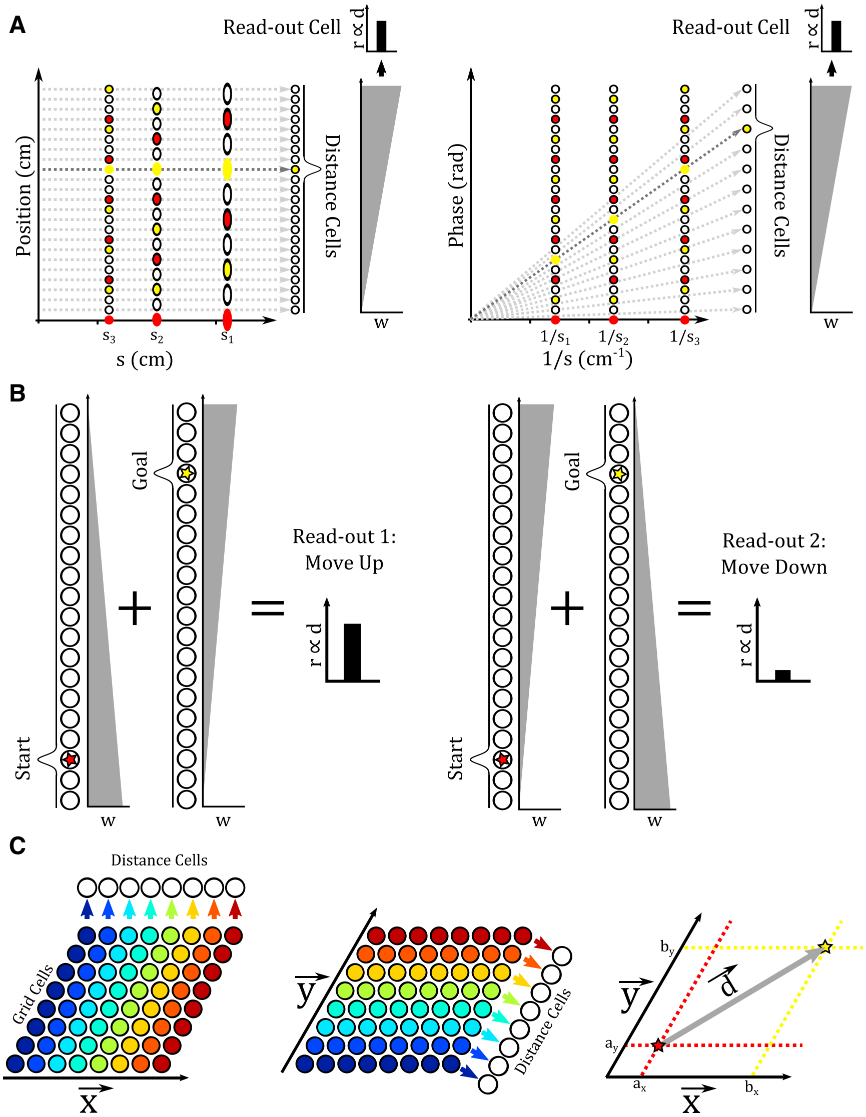

Figure 5. The Distance Cell Model

(A) An array of distance cells encode specific locations along a one-dimensional axis and receive input from grid cells that are active at each location along that axis. When grid cells that encode a goal location in each module fire, they activate a single distance cell that encodes that location, and winner-take-all dynamics eliminates activity in other distance cells. All distance cells provide input to a single readout cell with synaptic strengths that increase linearly with increasing displacement along the axis. The firing rate of that readout cell then encodes the displacement from the origin to the goal location along that axis.

(B) Combining two distance cell arrays allows the distance between arbitrary start and goal locations in either direction along the axis to be decoded. One distance cell array decodes the start location, and the other decodes the goal location. Each distance cell array projects to one "move up" (left) and one "move down" (right) readout cell with synaptic weights w that increase linearly in opposing directions along the axis. These readout cells then encode the displacement between start and goal locations in each direction along that axis.

(C) The distance cell model can be extended to two dimensions if all grid cells that share a common phase on each of at least two non-collinear axes project to the same distance cell. Combining the displacements encoded by the pair of readout cells for each axis provides the vector between start and goal locations in two-dimensional space. For full simulation details, see Supplemental Experimental Procedures and Figure S2.

The distance cell model can be extended to deal with arbitrary start and goal locations along the axis $\vec{x}$ by incorporating an additional array of distance cells and an additional readout cell, analogous to neural network models of the mental number line (Dehaene, 1997; Chen and Verguts, 2010). In this case, one distance cell array decodes current location a and the other decodes goal location b (Figure 5B). Both distance cell arrays project to both readout cells, but the strength of connections from each distance cell array to each readout cell increases in opposite directions along the axis $\vec{x}$. The relative firing rates of the two readout cells then encode the relative distance between current and goal locations along that axis in each direction (Figure 5B). Translation vectors in 2D space can be constructed from at least two pairs of distance cell arrays that decode current and goal positions $\vec{a}$ and $\vec{b}$ on non-collinear axes $\vec{x}$ and $\vec{y}$. In this case, each distance cell on each axis receives input from all grid cells within a module that share a common phase on that axis (Figure 5C). This model can also accommodate grid modules that differ in their ellipticity and orientation, provided that synaptic connections between grid cells and distance cells accurately project the position encoded by those grid cells onto the distance cell axis. Simulations demonstrate that the distance cell model can accurately decode translation vectors between arbitrary start and goal locations in large-scale 2D space (see Supplemental Experimental Procedures; Figure S2).

Vector Navigation by Direct Decoding: The "Rate-Coded Vector Cell" Model

The distance cell model independently decodes current and goal locations from sets of grid cell phases across modules $\{p_{x,i}\}$ before computing the linear displacement between them. As an alternative, it is possible to decode the linear displacement directly from the set of phase differences between grid cell representations at current and goal locations across modules $\{\Delta p_{x,i}\}$. In this "rate-coded vector cell" model, an array of vector cells each encode a specific displacement d from the current position along a single directional axis $\vec{x}$. Each vector cell receives input from all pairs of grid cells within each module whose unwrapped spatial phase difference $\Delta p_{x,i}$ along the axis $\vec{x}$ corresponds to that displacement, i.e., $d = ((\Delta p_{x,i}/2\pi) + n_i)s_i$ for some integer n_i, through multiplicative synapses (Figure 6A).

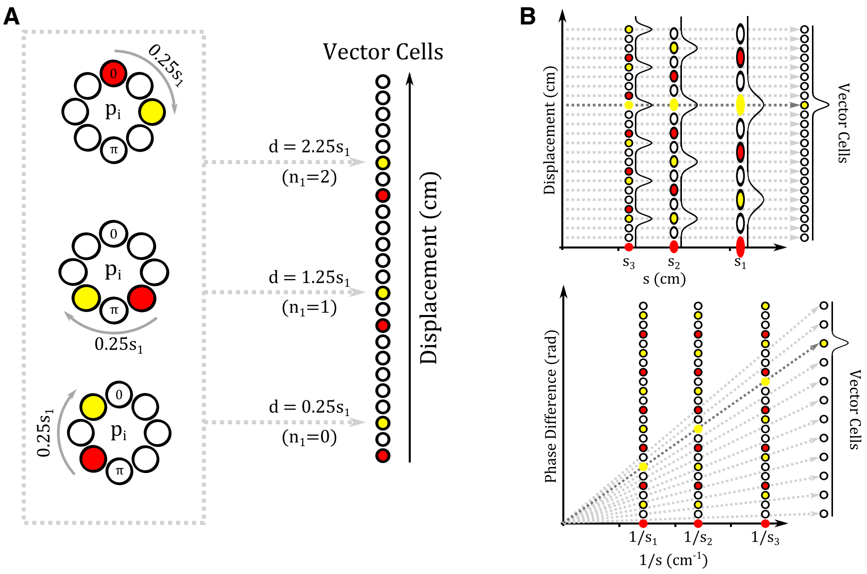

Figure 6. The Rate-Coded Vector Cell Model

(A) An array of vector cells encode specific displacements d along a one-dimensional axis $\vec{x}$ and receive input from pairs of grid cells within each module i encoding current (red) and goal (yellow) locations whose unwrapped phase difference $\Delta p_{x,i}$ corresponds to that displacement, i.e., $d = ((\Delta p_{x,i}/2\pi) + n_i)s_i$ for some integer n_i, where s_i is the grid scale.

(B) When grid cells encoding current (red) and goal (yellow) locations in each module fire simultaneously, they activate a single vector cell that encodes the consistent displacement across modules, and winner-take-all dynamics eliminates activity in other vector cells. Combining the activity of vector cells across at least two non-collinear axes provides the overall translation vector between start and goal locations in two-dimensional space. For full simulation details, see Supplemental Experimental Procedures and Figure S3.

Vector cells also receive input from all grid cell pairs in other modules whose unwrapped phase difference corresponds to the same absolute displacement d. Hence, each vector cell is maximally activated by a specific set of phase differences between current and goal locations across grid cell modules along axis $\vec{x}$ $\{\Delta p_{x,i}\}$ (Figure 6B). The total number of vector cells is limited by the capacity of the grid cell system to encode different displacements with unique sets of phase difference values across grid cell modules $\{\Delta p_i\}$ (see Supplemental Experimental Procedures; Figure S1). When grid cells encoding the current and goal locations across modules are simultaneously activated, winner-take-all dynamics ensure that only a single vector cell corresponding to the distance and direction between those locations becomes active. Translation vectors in 2D space can be constructed from at least two pairs of vector cell arrays that encode displacements in each direction on non-collinear axes $\vec{x}$ and $\vec{y}$. Simulations demonstrate that the rate-coded vector cell model can accurately decode translation vectors between arbitrary start and goal locations in large-scale 2D space (see Supplemental Experimental Procedures; Figure S3).

Vector Navigation by Direct Decoding: The "Phase-Coded Vector Cell" Model

As an alternative to the firing rate model described above, vector cells could make use of the temporal code for location provided by theta phase precession in grid cells. As animals transit through a firing field, a large proportion of grid cells fire spikes progressively earlier relative to the 5–11 Hz theta oscillation in the local field potential (LFP; Hafting et al., 2008; Reifenstein et al., 2012; Climer et al., 2013; Jeewajee et al., 2014; Figure 7A). In place cells, phase precession is stable across trials, while firing rates vary from trial to trial (Fenton and Muller, 1998; Huxter et al., 2003); and in both place and grid cells, phase precession scales with the size of firing fields (Huxter et al., 2003; Climer et al., 2013; Jeewajee et al., 2014) and conveys information about an animal's location beyond that encoded by the firing rate alone (Jensen and Lisman, 2000; Reifenstein et al., 2012). Importantly, phase precession dictates that the location of each grid field relative to the current location—i.e., the spatial phase difference Δp_i—is encoded in the theta firing phase of the corresponding grid cells.

Consider a population of grid cells that exhibit phase precession aligned with a specific 1D axis $\vec{x}$—that is, their theta firing phase encodes the distance traveled through the grid module along that axis, regardless of the trajectory taken (see Figure 7B; Climer et al., 2013; Jeewajee et al., 2014). Under these circumstances, the spatial phase difference between current location a and goal location b along that axis in module i ($\Delta p_{x,i}$; see Figure 2A) is proportional to the difference in theta firing phases of grid cells encoding the current location GC_a and goal location GC_b in that module, $\varnothing_i(GC_a)$ and $\varnothing_i(GC_b)$, i.e., $\Delta p_{x,i} \propto \Delta\varnothing_i$. If we assume that grid cells encoding the current location consistently fire at the trough of theta (i.e., ~0 rad), then the relative spatial phase of grid cells encoding the goal location b within each module will be proportional to their theta firing phase, i.e., $((b-a) mod\, s_i/s_i) \propto \varnothing_i(GC_b)$ (Figure 7C). Hence, the spatial phase difference $\Delta p_{x,i}$ between grid cells encoding current and goal locations within each module will also be proportional to the theta firing phase of grid cells encoding the goal location, i.e., $\Delta p_{x,i} \propto \varnothing_i(GC_b)$. Vector cells that are sensitive to a specific pattern of spike phases in grid cells encoding the goal location across modules can therefore directly decode the displacement between current and goal locations.

As an example, consider two grid cell modules of scales $s_1 = 30\ cm$ and $s_2 = 20\ cm$ on the 1D axis $\vec{x}$ and, for simplicity, assume that phase precession is linear and covers the full range of theta phase values—from π rad at field entry, through 0 rad at the field center to $-\pi$ rad at the exit. If the current location is $a = 0\ cm$, then grid cells encoding a goal location at $b = 30\ cm$ will fire at $\{\varnothing_i(GC_b)\} = \{0, \pi\ rad\}$ in the two modules, corresponding to the phase difference between their firing fields within each module $\Delta p_{x,i}$ (Figure 7D). Similarly, if the current location is $a = 45\ cm$, then grid cells encoding a goal location with the

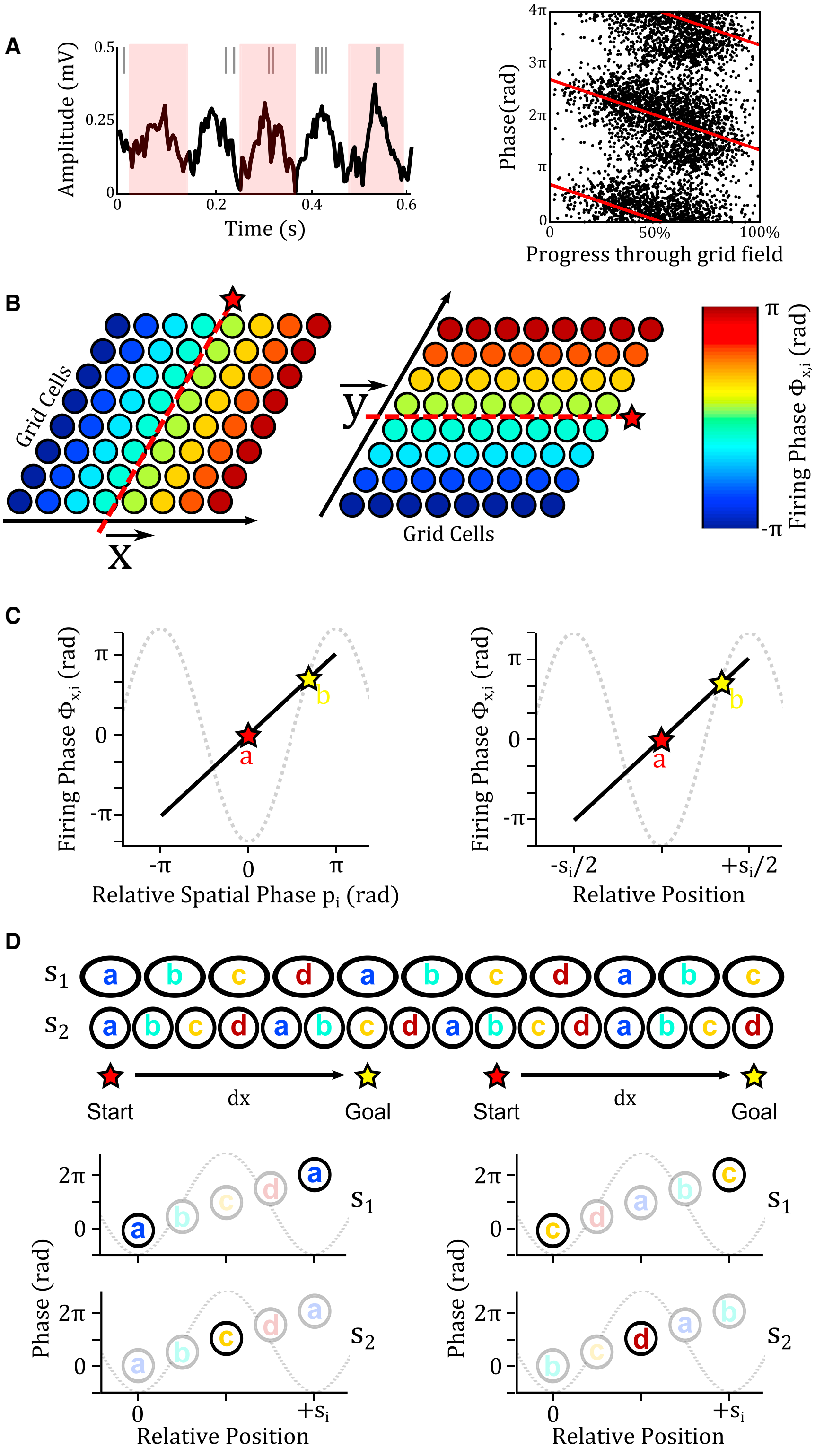

Figure 7. The Phase-Coded Vector Cell Model

(A) As animals transit through a firing field, a large proportion of grid cells exhibit theta phase precession, firing spikes progressively earlier relative to the 5–11 Hz theta oscillation in the local field potential. This results in an approximately linear relationship between firing phase and progress through the grid field.

(B) If phase precession in grid cells is aligned with a specific one-dimensional axis—that is, theta firing phase encodes the distance traveled through the grid module along that axis, regardless of the trajectory taken—then theta firing phase can be used to infer the relative spatial phase of any two grid cells within a module along that axis. Red dashed line/star indicates the current location on each axis.

(C) Phase precession aligned with a specific one-dimensional axis ensures that the difference in theta firing phase between grid cells encoding the current location *a* (which will be ~0 radians) and a goal location *b* is proportional to the difference in their spatial phase Δp_i or relative position within each grid module. Hence, if the goal location *b* is less than half a grid scale ahead of the current location *a* (as shown here), then grid cells encoding that location will fire at a later theta phase.

(D) The set of grid cells across modules *i* = 1 to *M* that encode a goal location at a set displacement from the current location along a specific one-dimensional axis will always fire at a specific combination of theta phase values $\{\varnothing_{x,i}\}$, irrespective of the current location. Here, we plot the theta firing phase at the current location of grid cells in two modules with scales s_i = {30, 20 cm} against the location of their firing fields relative to the current location. Grid cells encoding the current ("start") location always fire at the trough of theta (i.e., *0* rad), while grid cells encoding a goal location that is d = 30 cm from the current location along that axis are always encoded by grid cells firing at phases $\varnothing_x$ = {0, π rad}, respectively. Hence, an array of vector cells that are sensitive to specific combination of phase values $\varnothing_x$ across modules can decode the distance to the goal location along that axis. For full simulation details, see Supplemental Experimental Procedures and Figure S4.

same displacement, i.e., *b* = 75 *cm*, will again fire at $\{\varnothing_i(GC_b)\} = \{0, \pi\ rad\}$, corresponding to the same phase difference between their firing fields within each module $\Delta p_{x,i}$ (Figure 7D). Hence, if only grid cells encoding the goal location fire in a single theta cycle, then a vector cell that is sensitive to this specific pattern of firing phases in grid cells encoding the goal location across modules—i.e., $\{\varnothing_i(GC_b)\}$—can directly decode the displacement between current and goal locations. More generally, this result implies that the distance from the current position to every known goal location along that axis is encoded in each theta cycle by the relative phase of firing in grid cells across modules that encode those locations $\{\varnothing_i(GC_b)\}$. Translation vectors between current and goal locations in 2D space can be decoded from the pattern of firing phases in separate populations of grid cells that exhibit phase

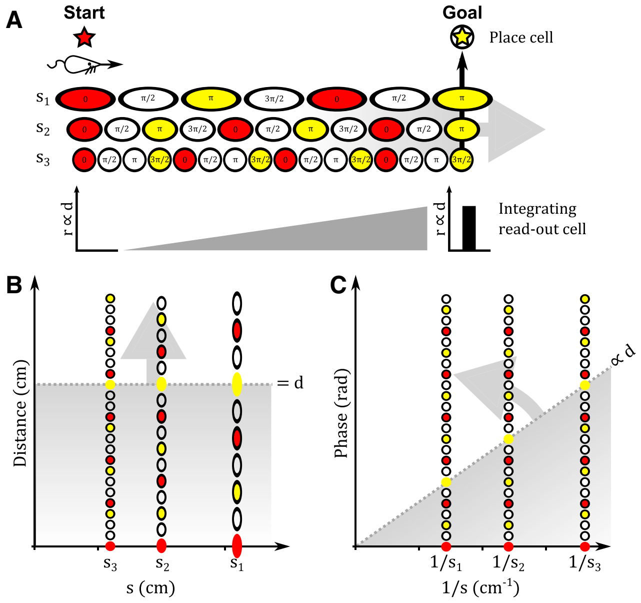

Figure 8. The Linear Look-Ahead Model

(A) During linear look-ahead events, activity in the grid cell network is initiated at the current (or goal) location and updated according to simulated movement away from that location along a specific one-dimensional axis. The firing rate of a single readout cell that integrates activity in one or more grid cell modules over time will then indicate the distance traveled along that axis. Arrival at the goal (or current) location is signaled by simultaneous activity in grid cells representing that location across modules.

(B and C) Linear look-ahead effectively performs a directed search for the goal (or current) location, starting from the current (or goal) location and moving sequentially through locations of increasing displacement along that directional axis. For full simulation details, see Supplemental Experimental Procedures and Figure S5.

precession aligned with two non-collinear axes $\vec{x}$ and $\vec{y}$ (Figure 7B). Simulations demonstrate that the phase-coded vector cell model can accurately decode translation vectors between arbitrary start and goal locations in large-scale 2D space (see Supplemental Experimental Procedures; Figure S4).

Vector Navigation by Directed Search: The "Linear Look Ahead" Model

An alternative to directly decoding the translation vector between current and goal locations is a directed search along specific 1D axes, beginning at either of those locations, in order to compute their relative position. During exploration, activity in the grid cell network is believed to reflect an animal's estimate of self-location that is updated by self-motion signals (Fuhs and Touretzky, 2006; McNaughton et al., 2006; Burgess et al., 2007). However, it is possible that simulated movement signals, decoupled from the animal's actual motion, could also be used to update the grid cell spatial representation, e.g., perform a "linear look ahead" (Erdem and Hasselmo, 2012; Kubie and Fenton, 2012; Erdem and Hasselmo, 2014) by simulating movement away from the current position *a* along an arbitrary axis $\vec{x}$ at a constant speed (Figure 8A). In the 1D case, this is equivalent to shifting the activity bump within each grid cell module around a putative ring attractor circuit at a rate corresponding to a constant spatial velocity across grid cell modules (i.e., faster for smaller scale modules; Figure 2A). The duration of the linear look ahead event, or the activity of a neuron that integrates total activity during the event, then encodes the displacement *d* of the represented location along the direction $\vec{x}$. The displacement of the goal location along that axis is signaled by simultaneous activity in grid cells encoding the goal location in each module, which could be achieved by coincidence detection in the corresponding place cell, for example (Figure 8A).

In effect, linear look ahead systematically searches for a match between phase values across modules $\{p_{x,i}\}$ that encode the goal location on the axis $\vec{x}$ with phase values that encode a sequence of positions moving away from the current location (Figures 8B and 8C). Alternatively, linear look ahead could be initiated from the goal location and systematically search for a match with phase values across grid modules that match the current location. Translation vectors in 2D space can be constructed using linear look ahead in each direction along at least two non-collinear axes, during which all grid cells that share a phase $\{p_{j,i}\}$ on each axis in each module are simultaneously active (Figure 5C). Hence, during each unidirectional linear look ahead, different sub-populations of grid cells fire simultaneously, according to their spatial phase on that axis. Simulations demonstrate that the linear look-ahead model can accurately decode translation vectors between arbitrary start and goal locations in large-scale 2D space (see Supplemental Experimental Procedures; Figure S5).

Critique of Grid Cell Vector Navigation Models

The direct decoding and linear look-ahead models described above exhibit divergent strengths and weaknesses and make different predictions for future experimental studies. Direct decoding models compute translation vectors quickly, without the need to search multiple possible solutions, while the linear look-ahead model predicts that the time required to compute translation vectors scales with their length, because the directed search takes longer to reach more distant locations (Figure S5C). This latter pattern might be more consistent with reports that human response times correlate with the length of imagined paths (Kosslyn et al., 1978) and metabolic activity in the hippocampal formation correlates with the distance to a goal during route planning (Sherrill et al., 2013; Howard et al., 2014).

Each of the direct decoding models requires significant additional neural circuitry to compute translation vectors. This raises the question as to how this circuitry develops or is learned during active navigation but provides experimental predictions regarding the existence of distance and readout or vector cell firing patterns. The distance cell model, for example, requires one or more neurons to encode each unique current and goal location on at least two principal axes, or at least four times as many distance cells as potential locations. These distance cells would exhibit a band-like firing pattern, as they encode a series of known allocentric locations along a specific 1D axis (Figure 5C) with a spatial periodicity equal to the capacity of the grid cell system. Synaptic connections from grid to distance cells could develop under a straightforward Hebbian learning rule during exploration, analogous to models of the grid to place cell transformation (Rolls et al., 2006; Solstad et al., 2006). Graded connection weights between distance and readout cells could be formed developmentally by Hebbian learning during the

propagation of a wave of activity along the distance cell population, while the readout cell firing rate increased gradually over time. This process need only occur once, as the same connectivity is then utilized across all environments.

The vector cell models require fewer additional neurons, as the need for an intermediate representation of absolute location is eliminated. This also allows the spatial resolution of vector cells to be reduced for greater encoded displacements, with translation vectors being dynamically recalculated as the goal is approached (see Supplemental Experimental Procedures; Figures S3 and S4). Vector cells would fire whenever an animal planned to navigate to any one of a band of goal locations at a fixed distance from the current location along a specific 1D axis. This response would be invariant to translation of the current and goal locations, in contrast to the purely allocentric co-ordinate frame utilized by distance cells. Synaptic connectivity between pairs of grid cells in each module and vector cells could be formed developmentally by Hebbian learning during the coordinated propagation of two parallel waves of activity across the sheet of grid cells in each module, where the separation between the waves reflects a specific phase difference along a 1D axis and the corresponding set of vector cells remain active. Again, we note that this process need only occur once. In the case of the rate-coded vector cell model, those synaptic connections must also be multiplicative, which lacks biological plausibility (but see Mel, 1993), although the same functionality could be achieved by the integration of inputs on distinct dendritic branches (London and Häusser, 2005). The phase-coded vector cell model avoids the need for multiplicative synapses but requires grid cells that exhibit phase precession aligned with specific 1D axes, and it is unclear from current data whether such temporal coding exists within grid cells of the mEC (Hafting et al., 2008; Reifenstein et al., 2012; Climer et al., 2013; Jeewajee et al., 2014).

Unlike direct decoding models, the linear look-ahead model makes use of neural mechanisms that are already in place to update grid cell activity according to self-motion. In continuous attractor network models of grid cell firing, directional input from conjunctive cells in deeper layers could drive grid cell activity during linear look ahead (Fuhs and Touretzky, 2006; Sargolini et al., 2006; Burak and Fiete, 2009); whereas in oscillatory interference models this input would come from velocity controlled oscillators (VCOs; Burgess et al., 2007; Burgess, 2008; Hasselmo, 2008; Welday et al., 2011). Independent of either model, the necessary synaptic connectivity could also develop through temporally asymmetric Hebbian learning within networks of conjunctive grid by head-direction cells in the deeper layers of mEC that would allow linear look ahead along the preferred firing direction of those cells (Sargolini et al., 2006; Kubie and Fenton, 2012).

Each of the direct decoding models predicts activity in grid cells encoding goal locations during route planning. It has been demonstrated that place cells in humans are reactivated during the retrieval of an episodic memory associated with that location (Miller et al., 2013), but whether similar reactivation occurs during route planning or in grid cells has yet to be determined. Importantly, the phase-coded vector cell model also predicts that the relative timing of this activity in grid cells across modules would correspond to their theta firing phase at the current location. Conversely, the linear look-ahead model predicts the sequential activation of bands of grid cells that share a common phase on a specific 1D axis during route planning, analogous to place cell replay and preplay events (Foster and Wilson, 2006; Davidson et al., 2009; Pfeiffer and Foster, 2013; see S.G. Trettel and L.L. Colgin, 2014, Soc. Neurosci., abstract for similar activity in grid cells during sleep). Interestingly, recent data indicates that place cell ripple related preplay can include novel routes (Ólafsdóttir et al., 2015)—a key property of vector navigation. However, the linear look-ahead model proposes activity sweeps along two non-collinear axes, not necessarily oriented toward the goal, in contrast to reports of goal-directed preplay (Pfeiffer and Foster, 2013).

DISCUSSION

We have described an algorithmic solution to the computational problem of large-scale vector navigation with grid cells. That is, how to accurately compute translation vectors between arbitrary locations in large-scale 2D space using the grid cell representations of those locations. This problem is the inverse of that thought to be performed by grid cells during path integration—extracting the translation vector between current and goal locations, as opposed to combining a previously known location with a subsequent movement vector to estimate the current location. Specifically, we have shown how the spatial phases of activity in grid cell modules of different spatial scales at start and goal locations can be used to extract the distance and direction between those locations. This is achieved by finding the maximum slope of a plane that fits the family of points defined by the phase difference in each grid module and the inverse scale of that module on at least two non-collinear axes (Figure 4C). Importantly, this solution is robust to differences in grid orientation between grid modules and ellipticity (i.e., differences in scale between axes) within each grid module (Stensola et al., 2012). This solution relates to the Fourier shift theorem, whereby the 2D translation applied to a basis set of Fourier components can be recovered from the phase changes across components (Orchard et al., 2013).

We have also described several neural network implementations of this algorithmic solution, building on a large body of previous work that has explored how grid cells efficiently encode location (Fiete et al., 2008; Mathis et al., 2012) and might contribute to vector navigation (Huhn et al., 2009; Masson and Girard, 2011; Erdem and Hasselmo, 2012; Kubie and Fenton, 2012; Erdem and Hasselmo, 2014). These models assume only that the grid representations of current and goal locations are known and produce direct vectors between those locations that may traverse previously unknown terrain. Each proposed implementation can decode 2D translation vectors with an accuracy and range that is comparable to the theoretical capacity of the grid cell system, and each model offers specific strengths, weaknesses, and experimental predictions. Several computational implementations that make use of the Chinese Remainder Theorem to perform this conversion have previously been proposed (Sun and Yao, 1994; Fiete et al., 2008; Masson and Girard, 2011). These models have limitations, however, such

as requiring a hard-wired energy landscape or readout weights, producing linear outputs that are only correct modulo the lowest common multiple of grid scales or performing gradient descent on an energy landscape with multiple local minima (Masson and Girard, 2011).

Two critical considerations for all grid cell models of vector navigation are whether grid cells provide a single, global representation of large-scale space and how vector navigation might be affected by local distortions of that representation. Several studies have demonstrated that grid firing patterns in isolated environments can become distorted or fragmented by local boundaries (Barry et al., 2007; Derdikman et al., 2009; Krupic et al., 2015; Stensola et al., 2015). These deformations will impair the ability of grid firing patterns to support vector navigation unless they affect all grid cell modules equally, which is not clear from current data (but see Stensola et al., 2012). Interestingly, however, it has recently been shown that grid firing patterns in two separate environments are initially local but become globally consistent when the animal is allowed sufficient experience of navigating between environments (Carpenter et al., 2015). Hence, given the opportunity to learn the relative location of different local environments within a larger space, grid cell firing patterns could provide a universal spatial metric for vector navigation across large distances.

It is also important to note that the vector navigation models described here cannot function in isolation. Grid cell firing patterns must be anchored to environmental sensory stimuli, both to prevent noise-related drift in the grid cell representation of space and to facilitate the subsequent planning and execution of real behavioral trajectories, which would incorporate the sensory and affective features of locations lying along the decoded vector. This sensory input might be mediated, in part, by projections from place, head direction, and boundary vector cells. Moreover, the allocentric translation vectors extracted from the grid cell network by each of the models presented here would generally need to be converted into egocentric movement strategies elsewhere in the brain before they could be utilized for actual navigation (Byrne et al., 2007).

Similarly, we note that both band cells (Burgess et al., 2007; Krupic et al., 2012; Mhatre et al., 2012) and velocity-controlled oscillators (VCOs), postulated by the oscillatory interference model (Burgess et al., 2007; Burgess, 2008; Hasselmo, 2008) and identified in the hippocampal formation (Welday et al., 2011), encode periodic spatial phase with a constant scale along a specific 1D axis. Such firing patterns correspond to Fourier components of a 2D spatial representation (Orchard et al., 2013) and could therefore be used in place of grid cell inputs to support both the algorithmic solutions and various neural network implementations presented here. Moreover, VCOs show the appropriate frequency dependence on velocity to encode displacement along a specific direction in their firing phase relative to the baseline (LFP) oscillation and subsequently support the phase-coded vector cell model. It is possible, therefore, that band cells or VCOs perform path integration and support vector navigation, while grid cells represent the interface between those cells and sensory information encoded by place cells (e.g., O'Keefe and Burgess, 2005; Bush et al., 2014). Further experiments are required to determine whether band cells or VCOs exist with the discrete range of spatial scales that would be required to support vector navigation over large distances.

To conclude, we have provided a theoretical framework within which to examine the computational problem of large-scale vector navigation using grid cells and presented an algorithmic solution to the problem and several biologically plausible implementations of that solution. Although the system we have described is focused on navigation, the same procedure could be applied to compute the displacement between any arbitrary pair of positions in any physical or conceptual space and in any number of dimensions. Future experiments must determine whether mEC is needed for vector navigation and, if so, what neurophysiological signatures it is associated with.

SUPPLEMENTAL INFORMATION

Supplemental Information includes Supplemental Experimental Procedures and five figures and can be found with this article online at http://dx.doi.org/10.1016/j.neuron.2015.07.006.

AUTHOR CONTRIBUTIONS

D.B. conceived the distance cell model and implemented all models; D.M. conceived the rate-coded vector cell model; C.B. conceived the phase-coded vector cell model; N.B. conceived the algorithmic solution; C.B. and N.B. instigated the research; and D.B., C.B., D.M., and N.B. wrote the manuscript.

ACKNOWLEDGMENTS

The authors would like to thank Andrej Bicanski, Francis Carpenter, Talfan Evans, Robin Hayman, and Aidan Horner for helpful comments. This work was supported by the Wellcome Trust, Medical Research Council UK, and the EU FP7 FET project SpaceCog.

Received: February 17, 2015
Revised: June 1, 2015
Accepted: July 13, 2015
Published: August 5, 2015

REFERENCES

Abrahams, S., Pickering, A., Polkey, C.E., and Morris, R.G. (1997). Spatial memory deficits in patients with unilateral damage to the right hippocampal formation. Neuropsychologia *35*, 11–24.

Barry, C., Hayman, R., Burgess, N., and Jeffery, K.J. (2007). Experience-dependent rescaling of entorhinal grids. Nat. Neurosci. *10*, 682–684.

Barry, C., Ginzberg, L.L., O'Keefe, J., and Burgess, N. (2012). Grid cell firing patterns signal environmental novelty by expansion. Proc. Natl. Acad. Sci. USA *109*, 17687–17692.

Bendig, A.W. (1952). Latent learning in a water maze. J. Exp. Psychol. *43*, 134–137.

Blum, K.I., and Abbott, L.F. (1996). A model of spatial map formation in the hippocampus of the rat. Neural Comput. *8*, 85–93.

Boccara, C.N., Sargolini, F., Thoresen, V.H., Solstad, T., Witter, M.P., Moser, E.I., and Moser, M.B. (2010). Grid cells in pre- and parasubiculum. Nat. Neurosci. *13*, 987–994.

Brown, M.A., and Sharp, P.E. (1995). Simulation of spatial learning in the Morris water maze by a neural network model of the hippocampal formation and nucleus accumbens. Hippocampus *5*, 171–188.

Burak, Y., and Fiete, I.R. (2009). Accurate path integration in continuous attractor network models of grid cells. PLoS Comput. Biol. *5*, e1000291.

Burgess, N. (2008). Grid cells and theta as oscillatory interference: theory and predictions. Hippocampus *18*, 1157–1174.

Burgess, C.P., and Burgess, N. (2014). Controlling phase noise in oscillatory interference models of grid cell firing. J. Neurosci. *34*, 6224–6232.

Burgess, N., and O'Keefe, J. (1996). Neuronal computations underlying the firing of place cells and their role in navigation. Hippocampus *6*, 749–762.

Burgess, N., Recce, M., and O'Keefe, J. (1994). A model of hippocampal function. Neural Netw. *7*, 1065–1081.

Burgess, N., Maguire, E.A., and O'Keefe, J. (2002). The human hippocampus and spatial and episodic memory. Neuron *35*, 625–641.

Burgess, N., Barry, C., and O'Keefe, J. (2007). An oscillatory interference model of grid cell firing. Hippocampus *17*, 801–812.

Bush, D., Barry, C., and Burgess, N. (2014). What do grid cells contribute to place cell firing? Trends Neurosci. *37*, 136–145.

Byrne, P., Becker, S., and Burgess, N. (2007). Remembering the past and imagining the future: a neural model of spatial memory and imagery. Psychol. Rev. *114*, 340–375.

Carpenter, F., Manson, D., Jeffery, K., Burgess, N., and Barry, C. (2015). Grid cells form a global representation of connected environments. Curr. Biol. *25*, 1176–1182.

Chen, Q., and Verguts, T. (2010). Beyond the mental number line: A neural network model of number-space interactions. Cognit. Psychol. *60*, 218–240.

Climer, J.R., Newman, E.L., and Hasselmo, M.E. (2013). Phase coding by grid cells in unconstrained environments: two-dimensional phase precession. Eur. J. Neurosci. *38*, 2526–2541.

Cohen, N.J., and Eichenbaum, H. (1993). Memory, Amnesia, and the Hippocampal System (MIT Press).

Conklin, J., and Eliasmith, C. (2005). A controlled attractor network model of path integration in the rat. J. Comput. Neurosci. *18*, 183–203.

Davidson, T.J., Kloosterman, F., and Wilson, M.A. (2009). Hippocampal replay of extended experience. Neuron *63*, 497–507.

Dayan, P. (1991). Navigating through temporal difference. NIPS *3*, 464–470.

Dehaene, S. (1997). The Number Sense: How the Mind Creates Mathematics (Oxford University Press).

Derdikman, D., Whitlock, J.R., Tsao, A., Fyhn, M., Hafting, T., Moser, M.-B., and Moser, E.I. (2009). Fragmentation of grid cell maps in a multicompartment environment. Nat. Neurosci. *12*, 1325–1332.

Doeller, C.F., and Burgess, N. (2008). Distinct error-correcting and incidental learning of location relative to landmarks and boundaries. Proc. Natl. Acad. Sci. USA *105*, 5909–5914.

Erdem, U.M., and Hasselmo, M. (2012). A goal-directed spatial navigation model using forward trajectory planning based on grid cells. Eur. J. Neurosci. *35*, 916–931.

Erdem, U.M., and Hasselmo, M.E. (2014). A biologically inspired hierarchical goal directed navigation model. J. Physiol. Paris *108*, 28–37.

Fenton, A.A., and Muller, R.U. (1998). Place cell discharge is extremely variable during individual passes of the rat through the firing field. Proc. Natl. Acad. Sci. USA *95*, 3182–3187.

Fenton, A.A., Kao, H.Y., Neymotin, S.A., Olypher, A., Vayntrub, Y., Lytton, W.W., and Ludvig, N. (2008). Unmasking the CA1 ensemble place code by exposures to small and large environments: more place cells and multiple, irregularly arranged, and expanded place fields in the larger space. J. Neurosci. *28*, 11250–11262.

Fiete, I.R., Burak, Y., and Brookings, T. (2008). What grid cells convey about rat location. J. Neurosci. *28*, 6858–6871.

Foster, D.J., and Wilson, M.A. (2006). Reverse replay of behavioural sequences in hippocampal place cells during the awake state. Nature *440*, 680–683.

Foster, D.J., Morris, R.G.M., and Dayan, P. (2000). A model of hippocampally dependent navigation, using the temporal difference learning rule. Hippocampus *10*, 1–16.

Fuhs, M.C., and Touretzky, D.S. (2006). A spin glass model of path integration in rat medial entorhinal cortex. J. Neurosci. *26*, 4266–4276.

Gallistel, C.R. (1990). The Organisation of Learning (MIT Press).

Gorchetchnikov, A., and Grossberg, S. (2007). Space, time and learning in the hippocampus: how fine spatial and temporal scales are expanded into population codes for behavioral control. Neural Netw. *20*, 182–193.

Guanella, A., Kiper, D., and Verschure, P. (2007). A model of grid cells based on a twisted torus topology. Int. J. Neural Syst. *17*, 231–240.

Hafting, T., Fyhn, M., Molden, S., Moser, M.B., and Moser, E.I. (2005). Microstructure of a spatial map in the entorhinal cortex. Nature *436*, 801–806.

Hafting, T., Fyhn, M., Bonnevie, T., Moser, M.B., and Moser, E.I. (2008). Hippocampus-independent phase precession in entorhinal grid cells. Nature *453*, 1248–1252.

Hartley, T., Maguire, E.A., Spiers, H.J., and Burgess, N. (2003). The well-worn route and the path less traveled: distinct neural bases of route following and wayfinding in humans. Neuron *37*, 877–888.

Hasselmo, M.E. (2008). Grid cell mechanisms and function: contributions of entorhinal persistent spiking and phase resetting. Hippocampus *18*, 1213–1229.

Hayward, A., McGregor, A., Good, M.A., and Pearce, J.M. (2003). Absence of overshadowing and blocking between landmarks and the geometric cues provided by the shape of a test arena. Q. J. Exp. Psychol. B *56*, 114–126.

Howard, L.R., Javadi, A.H., Yu, Y., Mill, R.D., Morrison, L.C., Knight, R., Loftus, M.M., Staskute, L., and Spiers, H.J. (2014). The hippocampus and entorhinal cortex encode the path and Euclidean distances to goals during navigation. Curr. Biol. *24*, 1331–1340.

Huhn, Z., Somogyvari, Z., Kiss, T., and Erdi, P. (2009). Extraction of distance information from the activity of entorhinal grid cells: a model study. In Proceedings of the International Joint Conference on Neural Networks, pp. 1298–1303.

Huxter, J., Burgess, N., and O'Keefe, J. (2003). Independent rate and temporal coding in hippocampal pyramidal cells. Nature *425*, 828–832.

Iaria, G., Petrides, M., Dagher, A., Pike, B., and Bohbot, V.D. (2003). Cognitive strategies dependent on the hippocampus and caudate nucleus in human navigation: variability and change with practice. J. Neurosci. *23*, 5945–5952.

Jeewajee, A., Barry, C., Douchamps, V., Manson, D., Lever, C., and Burgess, N. (2014). Theta phase precession of grid and place cell firing in open environments. Philos. Trans. R. Soc. Lond. B Biol. Sci. *369*, 20120532.

Jensen, O., and Lisman, J.E. (2000). Position reconstruction from an ensemble of hippocampal place cells: contribution of theta phase coding. J. Neurophysiol. *83*, 2602–2609.

Keith, J.R., and McVety, K.M. (1988). Latent place learning in a novel environment and the influences of prior training in rats. Psychobiology *16*, 146–151.

Kjelstrup, K.B., Solstad, T., Brun, V.H., Hafting, T., Leutgeb, S., Witter, M.P., Moser, E.I., and Moser, M.-B. (2008). Finite scale of spatial representation in the hippocampus. Science *321*, 140–143.

Kolb, B., and Whishaw, I.Q. (1996). Fundamentals of Human Neuropsychology (WH Freeman).

Kosslyn, S.M., Ball, T.M., and Reiser, B.J. (1978). Visual images preserve metric spatial information: evidence from studies of image scanning. J. Exp. Psychol. Hum. Percept. Perform. *4*, 47–60.

Krupic, J., Burgess, N., and O'Keefe, J. (2012). Neural representations of location composed of spatially periodic bands. Science *337*, 853–857.

Krupic, J., Bauza, M., Burton, S., Barry, C., and O'Keefe, J. (2015). Grid cell symmetry is shaped by environmental geometry. Nature *518*, 232–235.

Kubie, J.L., and Fenton, A.A. (2012). Linear look-ahead in conjunctive cells: an entorhinal mechanism for vector-based navigation. Front. Neural Circuits *6*, 20.

London, M., and Häusser, M. (2005). Dendritic computation. Annu. Rev. Neurosci. *28*, 503–532.

Maguire, E.A., Burgess, N., Donnett, J.G., Frackowiak, R.S., Frith, C.D., and O'Keefe, J. (1998). Knowing where and getting there: a human navigation network. Science *280*, 921–924.

Marr, D., and Poggio, T. (1977). From understanding computation to understanding neural circuitry. Neurosci. Res. Program Bull. *15*, 470–488.

Masson, C., and Girard, B. (2011). Decoding the grid cells for metric navigation using the residue numeral system. In Advances in Cognitive Neurodynamics, R. Wang and F. Gu, eds. (Springer), pp. 459–464.

Mathis, A., Herz, A.V., and Stemmler, M. (2012). Optimal population codes for space: grid cells outperform place cells. Neural Comput. *24*, 2280–2317.

Mathis, A., Herz, A.V.M., and Stemmler, M.B. (2013). Multiscale codes in the nervous system: the problem of noise correlations and the ambiguity of periodic scales. Phys. Rev. E Stat. Nonlin. Soft Matter Phys. *88*, 022713.

McNaughton, B.L., Battaglia, F.P., Jensen, O., Moser, E.I., and Moser, M.B. (2006). Path integration and the neural basis of the 'cognitive map'. Nat. Rev. Neurosci. *7*, 663–678.

Mel, B.W. (1993). Synaptic integration in an excitable dendritic tree. J. Neurophysiol. *70*, 1086–1101.

Mhatre, H., Gorchetchnikov, A., and Grossberg, S. (2012). Grid cell hexagonal patterns formed by fast self-organized learning within entorhinal cortex. Hippocampus *22*, 320–334.

Miller, J.F., Neufang, M., Solway, A., Brandt, A., Trippel, M., Mader, I., Hefft, S., Merkow, M., Polyn, S.M., Jacobs, J., et al. (2013). Neural activity in human hippocampal formation reveals the spatial context of retrieved memories. Science *342*, 1111–1114.

Morris, R.G., Garrud, P., Rawlins, J.N., and O'Keefe, J. (1982). Place navigation impaired in rats with hippocampal lesions. Nature *297*, 681–683.

Muller, R.U., and Kubie, J.L. (1987). The effects of changes in the environment on the spatial firing of hippocampal complex-spike cells. J. Neurosci. *7*, 1951–1968.

O'Keefe, J., and Burgess, N. (2005). Dual phase and rate coding in hippocampal place cells: theoretical significance and relationship to entorhinal grid cells. Hippocampus *15*, 853–866.

O'Keefe, J., and Conway, D.H. (1978). Hippocampal place units in the freely moving rat: why they fire where they fire. Exp. Brain Res. *31*, 573–590.

O'Keefe, J., and Dostrovsky, J. (1971). The hippocampus as a spatial map. Preliminary evidence from unit activity in the freely-moving rat. Brain Res. *34*, 171–175.

O'Keefe, J., and Nadel, L. (1978). The Hippocampus as a Cognitive Map (Oxford University Press).

Ólafsdóttir, H.F., Barry, C., Saleem, A.B., Hassabis, D., and Spiers, H.J. (2015). Hippocampal place cells construct reward related sequences through unexplored space. eLife. Published online June 26, 2015. http://dx.doi.org/10.7554/eLife.06063.

Orchard, J., Yang, H., and Ji, X. (2013). Does the entorhinal cortex use the Fourier transform? Front. Comput. Neurosci. *7*, 179.

Packard, M.G., and McGaugh, J.L. (1996). Inactivation of hippocampus or caudate nucleus with lidocaine differentially affects expression of place and response learning. Neurobiol. Learn. Mem. *65*, 65–72.

Parron, C., and Save, E. (2004). Evidence for entorhinal and parietal cortices involvement in path integration in the rat. Exp. Brain Res. *159*, 349–359.

Pfeiffer, B.E., and Foster, D.J. (2013). Hippocampal place-cell sequences depict future paths to remembered goals. Nature *497*, 74–79.

Reifenstein, E.T., Kempter, R., Schreiber, S., Stemmler, M.B., and Herz, A.V. (2012). Grid cells in rat entorhinal cortex encode physical space with independent firing fields and phase precession at the single-trial level. Proc. Natl. Acad. Sci. USA *109*, 6301–6306.

Rolls, E.T., Stringer, S.M., and Elliot, T. (2006). Entorhinal cortex grid cells can map to hippocampal place cells by competitive learning. Network *17*, 447–465.

Sargolini, F., Fyhn, M., Hafting, T., McNaughton, B.L., Witter, M.P., Moser, M.B., and Moser, E.I. (2006). Conjunctive representation of position, direction, and velocity in entorhinal cortex. Science *312*, 758–762.

Scoville, W.B., and Milner, B. (1957). Loss of recent memory after bilateral hippocampal lesions. J. Neurol. Neurosurg. Psychiatry *20*, 11–21.

Sharp, P.E., Blair, H.T., and Brown, M. (1996). Neural network modeling of the hippocampal formation spatial signals and their possible role in navigation: a modular approach. Hippocampus *6*, 720–734.

Sherrill, K.R., Erdem, U.M., Ross, R.S., Brown, T.I., Hasselmo, M.E., and Stern, C.E. (2013). Hippocampus and retrosplenial cortex combine path integration signals for successful navigation. J. Neurosci. *33*, 19304–19313.

Solstad, T., Moser, E.I., and Einevoll, G.T. (2006). From grid cells to place cells: a mathematical model. Hippocampus *16*, 1026–1031.

Squire, L.R., and Zola-Morgan, S. (1991). The medial temporal lobe memory system. Science *253*, 1380–1386.

Sreenivasan, S., and Fiete, I. (2011). Grid cells generate an analog error-correcting code for singularly precise neural computation. Nat. Neurosci. *14*, 1330–1337.

Steele, R.J., and Morris, R.G. (1999). Delay-dependent impairment of a matching-to-place task with chronic and intrahippocampal infusion of the NMDA-antagonist D-AP5. Hippocampus *9*, 118–136.

Steffenach, H.A., Witter, M., Moser, M.-B., and Moser, E.I. (2005). Spatial memory in the rat requires the dorsolateral band of the entorhinal cortex. Neuron *45*, 301–313.

Stensola, H., Stensola, T., Solstad, T., Frøland, K., Moser, M.B., and Moser, E.I. (2012). The entorhinal grid map is discretized. Nature *492*, 72–78.

Stensola, T., Stensola, H., Moser, M.-B., and Moser, E.I. (2015). Shearing-induced asymmetry in entorhinal grid cells. Nature *518*, 207–212.

Sun, H., and Yao, T.-R. (1994). A neural-like network approach to residue-to-decimal conversion. Neural Netw. *6*, 3883–3887.

Sutton, R.S., and Barto, A.G. (1998). Reinforcement Learning: An Introduction (MIT Press).

Taube, J.S., Muller, R.U., and Ranck, J.B., Jr. (1990). Head-direction cells recorded from the postsubiculum in freely moving rats. I. Description and quantitative analysis. J. Neurosci. *10*, 420–435.

Thompson, L.T., and Best, P.J. (1989). Place cells and silent cells in the hippocampus of freely-behaving rats. J. Neurosci. *9*, 2382–2390.

Tolman, E.C. (1948). Cognitive maps in rats and men. Psychol. Rev. *55*, 189–208.

Touretzky, D.S., and Redish, A.D. (1996). Theory of rodent navigation based on interacting representations of space. Hippocampus *6*, 247–270.

Van Cauter, T., Camon, J., Alvernhe, A., Elduayen, C., Sargolini, F., and Save, E. (2013). Distinct roles of medial and lateral entorhinal cortex in spatial cognition. Cereb. Cortex *23*, 451–459.

Wei, X.X., Prentice, J., and Balasubramanian, V. (2013). The sense of place: grid cells in the brain and the transcendental number e. arXiv, arXiv:1304.0031, http://arxiv.org/abs/1304.0031.

Welday, A.C., Shlifer, I.G., Bloom, M.L., Zhang, K., and Blair, H.T. (2011). Cosine directional tuning of theta cell burst frequencies: evidence for spatial coding by oscillatory interference. J. Neurosci. *31*, 16157–16176.

Neuron

Supplemental Information

Using Grid Cells for Navigation

Daniel Bush, Caswell Barry, Daniel Manson, and Neil Burgess

Supplemental Data

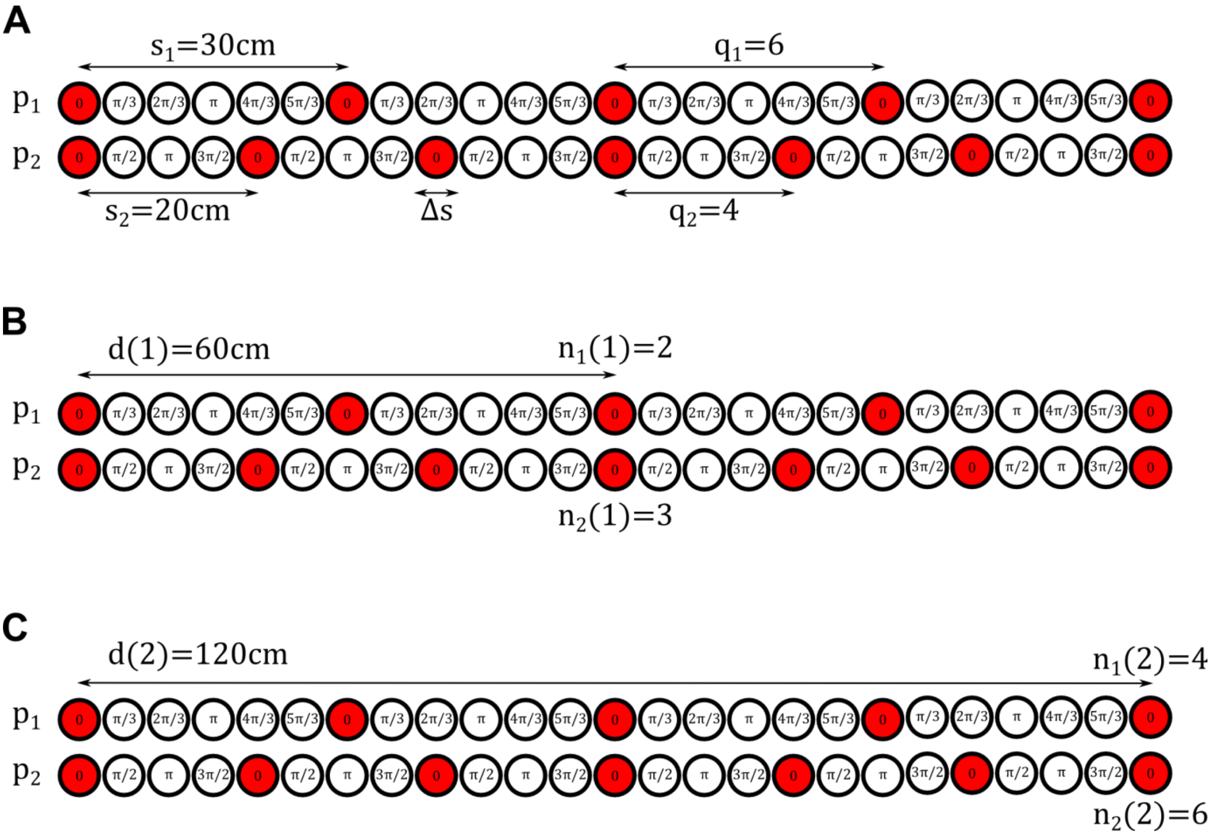

Figure S1: Capacity of the Grid Cell System (see also Algorithmic Solution in 1D, Supplemental Experimental Procedures). The displacement d of a location encoded by a specific combination of grid cell phases p_i across modules must satisfy the equation $d(k) = s_i(p_i/2\pi + n_i(k))$ for all $i = 1$ to M (Equation S1), where s_i is the scale of module i and n_i an integer value for that module. There are multiple solutions to this equation, indexed here by k, such that specific combinations of grid cell phases across modules correspond to multiple, periodically spaced locations along the axis. The capacity of the grid cell system is defined as the distance between consecutive solutions $d(k+1) - d(k)$ (Equation S2). If we express each grid scale s_i as an integer multiple q_i of the distance resolution of coding across modules Δs (i.e. $s_i = q_i \Delta s$, but see Supplemental Experimental Procedures), then we can show that the distance between successive locations $d(k+1) - d(k)$ is equal to the lowest common multiple of $\{q_i\}$ multiplied by the distance resolution Δs (Equation S5). In the example shown here, $M = 2$ grid cell modules with a distance resolution $\Delta s = 5cm$ have scales of $s_1 = 30cm$ and $s_2 = 20cm$ (i.e. $q_1 = 6$ and $q_2 = 4$). When both grid modules have a phase of $p_i = 0\, rad$, **(A)** the first solution to Equation S1 is $d(0) = 0cm$, given by $n_1 = 0$ and $n_2 = 0$; **(B)** the second solution is $d(1) = 60cm$, given by $n_1 = 2$ and $n_2 = 3$; and **(C)** the third solution is $d(2) = 120cm$, given by $n_1 = 4$ and $n_2 = 6$. Hence, the capacity of the grid cell system $d(k+1) - d(k) = 60cm$ which is equal to the lowest common multiple of $q_1 = 6$ and $q_2 = 4$, i.e. 12, multiplied by the distance resolution $\Delta s = 5cm$.

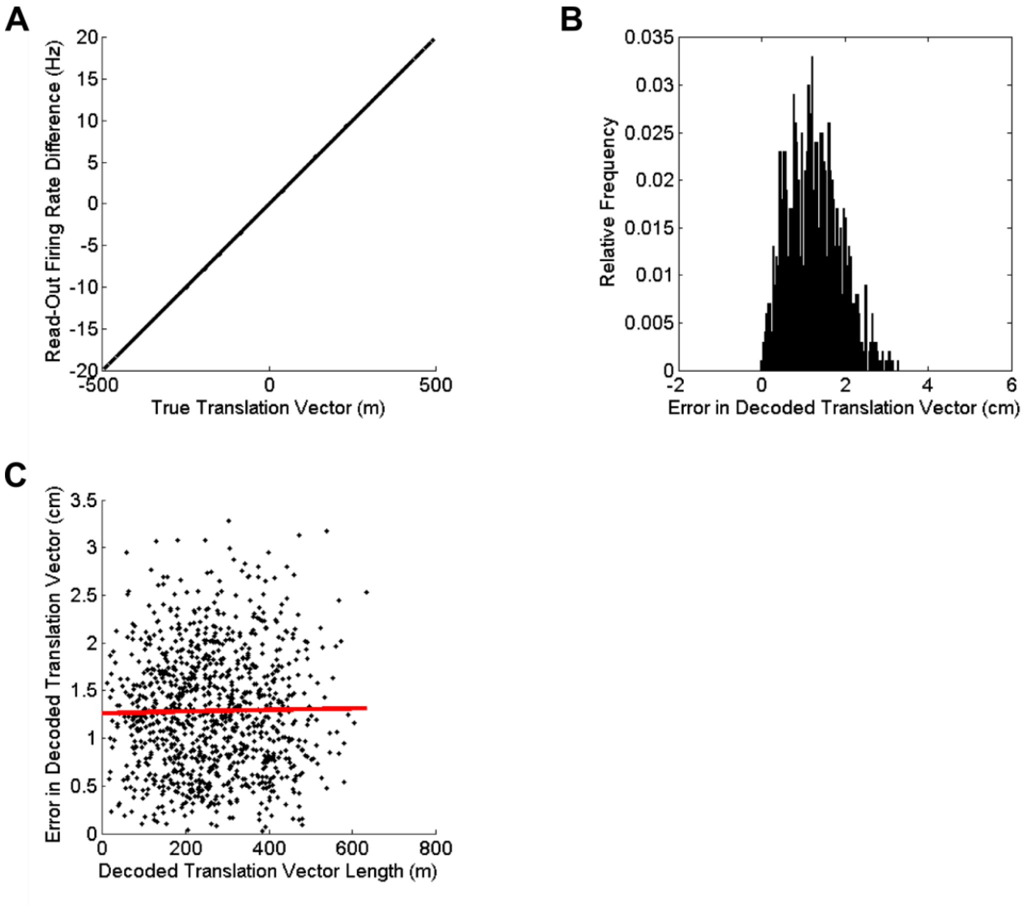

Figure S2: Simulations of the Distance Cell Model (see also Figure 5, Supplemental Experimental Procedures). We postulate two arrays of N_{DC}=12500 distance cells that encode start and goal locations, respectively, on each of two axes $\vec{x}$ and $\vec{y}$. Grid cells in each module project to distance cells with synaptic weights that are proportional to their mean firing rate at the corresponding location on that axis (Figure 5A). The number of spikes fired by N_{GC}=400 grid cells, representing m_i=20 equally distributed spatial phases p_i along each axis, is given by a Poisson process with a rate function that is cosine tuned for location along that axis (Equation S6) and a time window of 100ms. Distance cells in each array are subject to winner-take-all dynamics, and all distance cells in each array project to a pair of read-out cells with synaptic weights that increase topographically in different directions along each axis (Figure 5B). Hence, the relative firing rate of those read-out cells encodes the displacement between start and goal locations along that axis. **(A)** The difference in read-out cell firing rates (collapsed across both principal axes) plotted against the true translation vector for N=1000 simulations with randomly assigned start and goal locations in a 500m sided 2D arena (see Experimental Procedures). This illustrates that translation vectors can be accurately decoded from the relative firing rate of read-out cells. **(B)** The distribution of errors in 2D translation vector lengths decoded from the relative firing rate of read-out cells across the same N=1000 simulations. Note that distance cells were given a resolution of 4cm in these simulations. **(C)** Total decoded 2D translation vector lengths plotted against decoding error. This demonstrates that there is no correlation (r=0.017, p=0.60) between the length of translation vectors and the decoding error, as the spatial resolution of distance cells is even across the entire 2D arena.

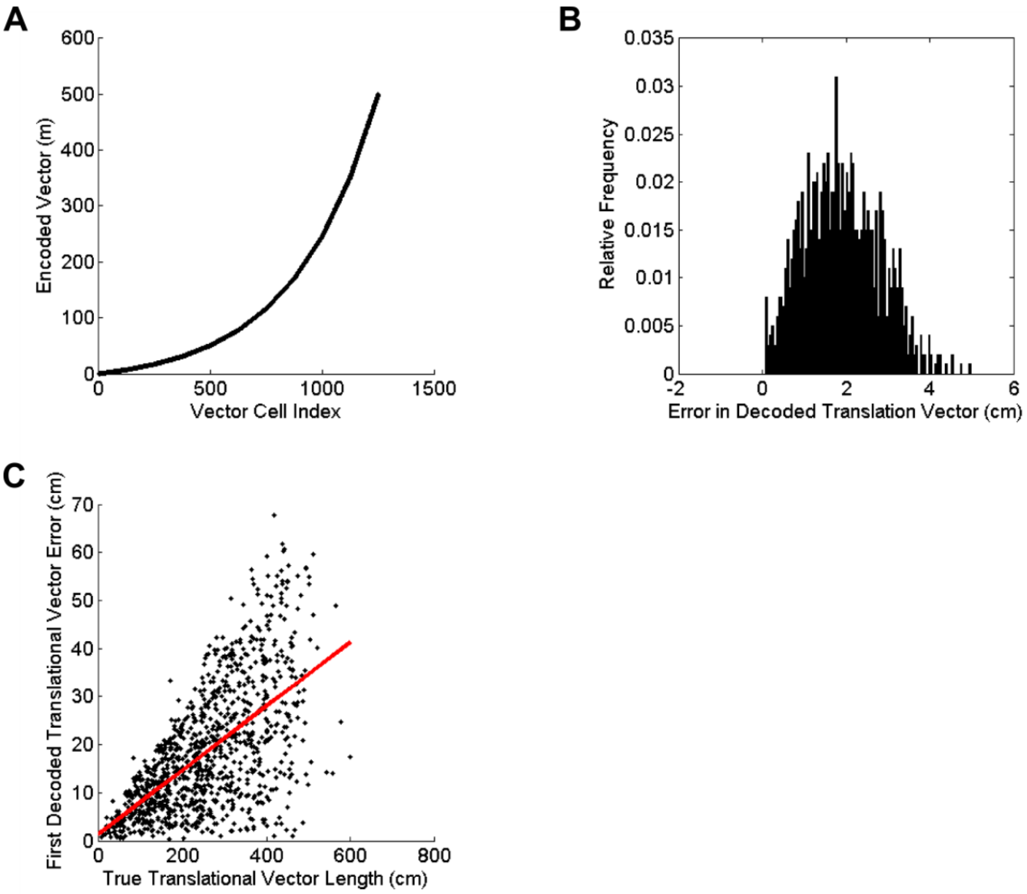

Figure S3: Simulations of the Rate-coded Vector Cell Model (see also Figure 6, Supplemental Experimental Procedures). We postulate two arrays of N_{VC}=1250 vector cells that encode the displacement between start and goal locations in either direction along each of two axes $\vec{x}$ and $\vec{y}$. Vector cells receive input from grid cell pairs within each module whose unwrapped phase difference corresponds to that absolute vector on that axis through multiplicative synapses (Figure 6A). The number of spikes fired by N_{GC}=400 grid cells, representing m_i=20 equally distributed spatial phases p_i along each axis, is given by a Poisson process with a rate function that is cosine tuned for location along that axis (Equation S6) and a time window of 100ms. Vector cells in each array are subject to winner-take-all dynamics, and translation vectors are directly decoded from the weighted mean of vector cell activity. Translation vectors are calculated iteratively, with the start location updated by 80% of the total decoded vector length along each axis prior to each new calculation, until the start location is within 1m of the true goal. **(A)** Pseudo-exponential distribution of translation vectors encoded by vector cells. **(B)** The distribution of translation vector errors decoded in the final iterative step from the activity of vector cells across N=1000 simulations with randomly assigned start and goal locations in a 500m sided 2D arena (see Experimental Procedures). **(C)** Total decoded 2D translation vector lengths plotted against decoding error in the first iterative step. This demonstrates that there is a significant correlation (r=0.61, p<0.001) between the length of translation vectors and initial decoding error, as the spatial resolution of vector cells decreases with increasing encoded displacement (Figure S3A).

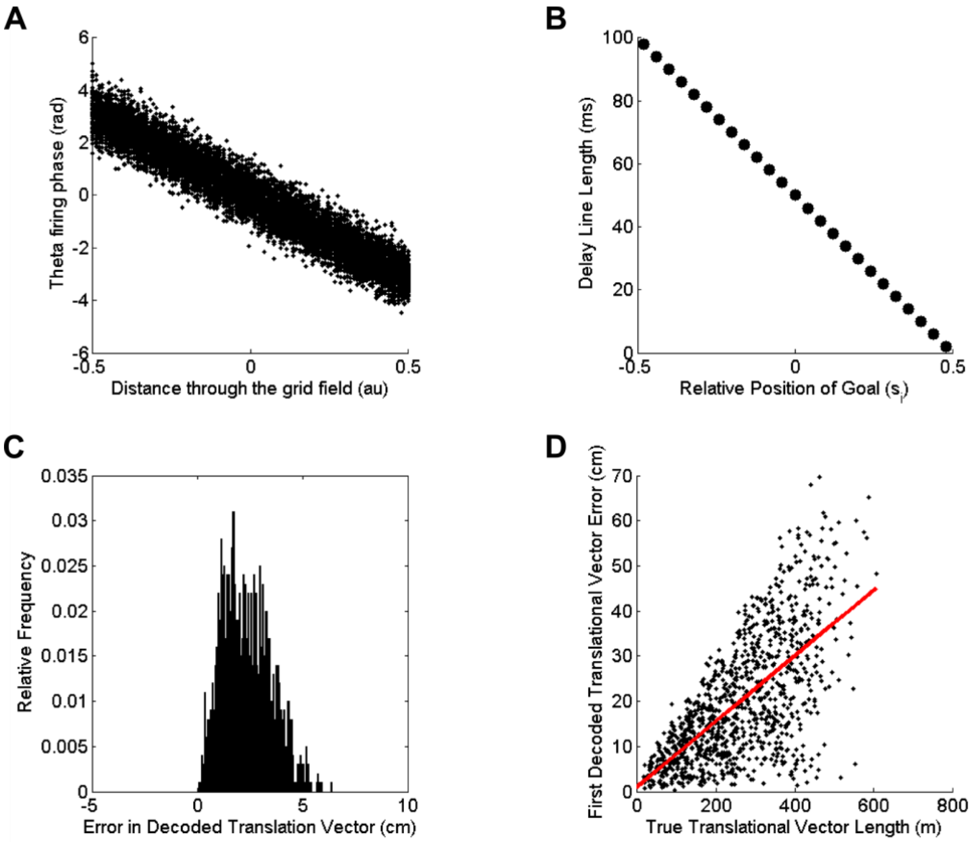

Figure S4: Simulations of the Phase-coded Vector Cell Model (see also Figure 7, Supplemental Experimental Procedures). We postulate two populations of grid cells that exhibit phase precession aligned with specific non-collinear axes (Figure 7A, B); and that only the grid cells within each module which exhibit the maximum rate at the goal location are reactivated, firing one spike within a single t_{theta}=100ms at a theta phase consistent with the current location. **(A)** Theta firing phases are drawn from a circular normal distribution with a mean that is linearly correlated with the distance (modulo grid scale) between the peak of that grid cell's firing field and the current location along the axis of phase precession (Equation S7) and a circular standard deviation of $\mu = {}^{\pi}/_{6}$ rad (compare to Figure 7A). **(B)** Grid cells project to vector cells through delay lines with transmission delays which ensure that spikes from grid cells encoding the goal location across modules arrive simultaneously at the corresponding vector cell (Equation S8). The activity of vector cells is determined by the temporal proximity of incoming spikes, i.e. vector cells perform temporal coincidence detection on the inputs that arrive from grid cells through delay lines. Vector cells in each array are subject to winner-take-all dynamics, and translation vectors can be directly decoded from the weighted mean of vector cell activity. Translation vectors are calculated iteratively, with the start location updated by 80% of the total decoded vector length along each axis prior to each new calculation, until the start location is within 1m of the true goal. **(C)** The distribution of translation vector errors decoded in the final iterative step from the activity of vector cells across *N*=1000 simulations with randomly assigned start and goal locations in a 500m sided 2D arena (see Experimental Procedures). **(D)** Total decoded 2D vector lengths plotted against decoding error in the first iterative step. This demonstrates that there is a significant correlation (r=0.66, p<0.001) between the length of translation vectors and initial decoding error, as the spatial resolution of vector cells decreases with increasing encoded displacement (Figure S3A).

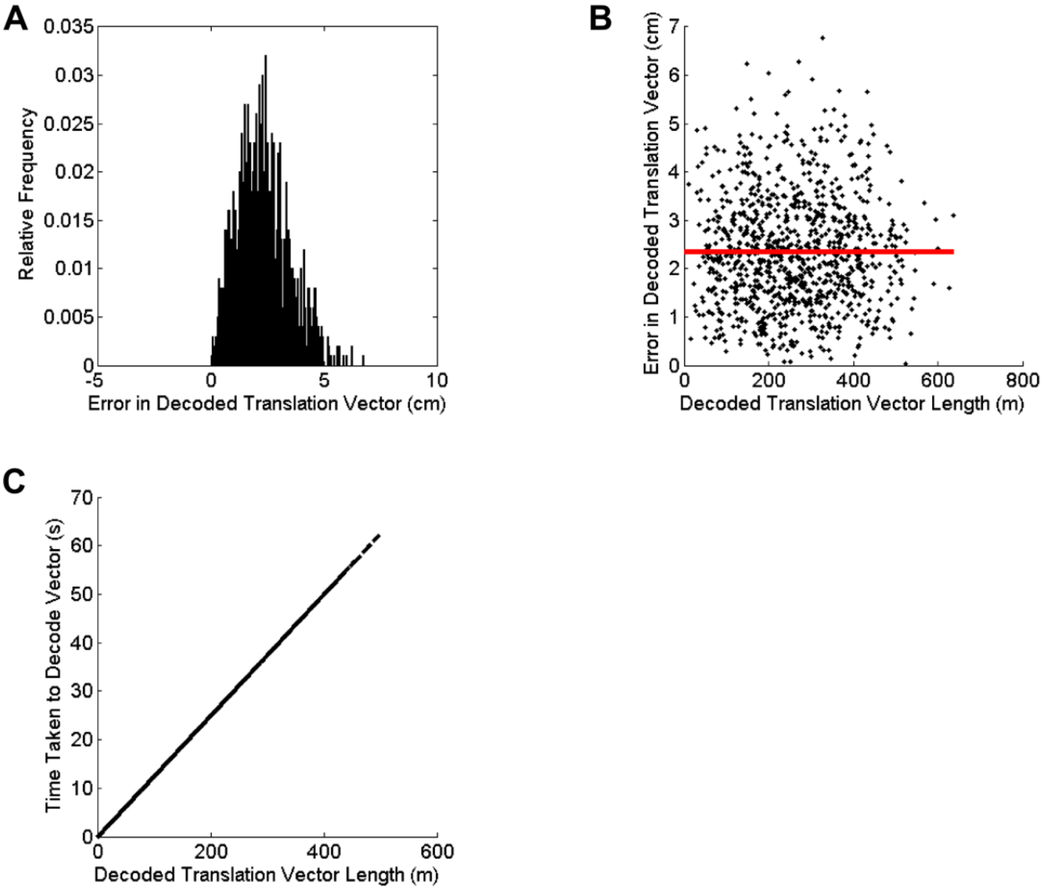

Figure S5: Simulations of the Linear Look-ahead Model (see also Figure 8, Supplemental Experimental Procedures). Linear look ahead activity is initiated by setting grid cell firing rates in each module to match those at the start location and then updating those firing rates in each subsequent 5ms time step according to simulated movement away from the current location at a constant speed of $8ms^{-1}$ along a specific directional axis. Grid cells in each module project to an array of place cells that evenly cover the 2D arena with synaptic weights that are proportional to their mean firing rate at the centre of the place field, and place cells are subject to winner-take-all dynamics. The time taken for grid cells encoding the goal location across modules to become simultaneously active, or the firing rate of a neuron that integrates total activity in the grid cell network during linear look ahead, subsequently encodes the displacement between start and goal locations along that axis (Figure 8A). This is indicated by activity in the corresponding place cell. The number of spikes fired by N_{GC}=400 grid cells, representing m_i=20 equally distributed spatial phases p_i along each axis, is given by a Poisson process with a rate function that is cosine tuned for location along that axis (Equation S6). Overall translation vectors in 2D are constructed from linear look ahead in each direction along two non-collinear axes. **(A)** The distribution of decoding errors across N=1000 simulations with randomly assigned start and goal locations in a 500m sided 2D arena. **(B)** Total decoded 2D translation vector lengths plotted against decoding error. This demonstrates that there is no correlation (r=0.017, p=0.60) between the length of translation vectors and the decoding error, as place cell firing fields evenly cover the entire 2D arena. **(C)** The time taken to decode translation vectors along each axis (i.e. the length of each linear look ahead event) is determined by the displacement between start and goal locations along that axis.

Supplemental Experimental Procedures

Capacity of the Grid Cell System

We can calculate the capacity of the grid cell system within our formalism (following Fiete et al., 2008). If we consider multiple distinct solutions $d(k)$ that satisfy Equations 1 and 2 for different sets of integer values $n_i(k)$, where $i = 1$ to M, then from Equation 1 (or Figure 4A) we have:

$$d(k) = s_i(p_i/2\pi + n_i(k)) \qquad \text{for all } i = 1 \text{ to } M$$

[Equation S1]

The distance between adjacent solutions is:

$$d(k+1) - d(k) = s_i(n_i(k+1) - n_i(k)) \qquad \text{for all } i = 1 \text{ to } M$$

[Equation S2]

If we express s_i in integer multiples $\{q_i\}$ of the distance resolution of coding within each grid module (i.e. the minimum distinguishable distance, Δs, which we assume to be constant across modules, but see below), so that $s_i = q_i \Delta s$, then:

$$\frac{(d(k+1) - d(k))}{\Delta s} = (n_i(k+1) - n_i(k))q_i \quad \text{for all } i = 1 \text{ to } M$$

[Equation S3]

If there are no common factors in $\{q_i\}$, where $i = 1$ to M, then Equation S3 requires that:

$$\big(n_i(k+1) - n_i(k)\big) = \prod_j q_j \qquad \text{for } j = 1 \text{ to } M, j \neq i$$

[Equation S4]

so that:

$$d(k+1) - d(k) = \Delta s \prod_j q_j \qquad \text{for } j = 1 \text{ to } M$$

[Equation S5]

Thus the separation between consecutive ambiguous solutions is the product of the grid scales in units of the minimum distinguishable distance in a grid module (unless there are common factors in $\{q_i\}$, in which case the separation is equal to the least common multiple of grid scales; see Fiete et al., 2008), and displacements within this range are encoded unambiguously. As a consequence, grid scales should avoid common factors, or the separation between ambiguous solutions will be correspondingly reduced (for further discussion, see Mathis et al., 2012, 2013; Towse et al., 2014). However, a fixed distance resolution across modules seems unlikely, assuming that neurons in different modules have common biophysical properties and given the apparently greater number of grid cells with small rather than large scales (Stensola et al., 2012). A more realistic assumption would be a fixed phase resolution across modules (i.e. distance resolution is proportional to grid scale, $\Delta s_i \propto s_i$), in which case Fiete et al. (2008) show that capacity has a similar exponential dependence on the number of modules *M*.

Neural Network Simulations

In all simulations, locations are encoded by M=10 grid cell modules whose spatial scales are arranged in a geometric progression (i.e. $s_i = s_M\alpha^{M-i}$) with a minimum grid scale of s_{10}=25cm and a common factor of α=1.4 (giving a maximum grid scale of ~5m). Each module consists of N_{GC}=400 grid cells distributed evenly among m_i=20 equally distributed spatial phases p_i. We note that the exact distribution of grid scales has no effect on the simulations presented here, aside from determining the capacity of the grid cell system to encode unique locations (Fiete et al., 2008; Mathis et al., 2012; Wei et al., 2013; Towse et al., 2014; see 'Capacity of the Grid Cell System' above) and the ability of the grid cell system to deal with neural noise (Sreenivasan and Fiete, 2011; Mathis et al., 2013).

For each model, translation vectors are decoded for N=1000 randomly assigned current and goal locations in a 2D arena. These locations are constructed from randomly assigned displacements along two 500m principal axes of that arena. For simplicity, these axes are oriented at $\vec{x} = 0°$ and $\vec{y} = 60°$ to match the principal axes of the grid pattern. We note that the size of this arena is significantly less than the capacity of this grid cell network to encode locations with a unique set of grid cell phases across modules. Using the formalism above and a distance resolution across modules of $\Delta s = 0.4m$, which represents the maximum accuracy with which distances can be resolved from noisy grid cell firing in the largest grid module alone, we estimate a capacity of ~3.3km for the grid cell system examined here.

In all rate-coded simulations, the firing of grid cells is modelled as a Poisson process with a rate function $r_{j,i}$ that exhibits cosine tuning for location a in module i along each principal axis u_j and a maximum rate r_{max}=30Hz (Equation S6):

$$r_{j,i}(a) = r_{max}\frac{1 + \cos\left(\left[\frac{a - s_i\left(\frac{p_{j,i}}{2\pi}\right)}{s_i}\right] \times 2\pi\right)}{2}$$

[Equation S6]

All Matlab code used to perform these simulations is available on ModelDB (http://modeldb.yale.edu/182685).

The Distance Cell Model

In distance cell simulations, distance cells encode current or goal locations in allocentric space along the principal axes $\vec{x}$ and $\vec{y}$ (Fiete et al., 2008; Huhn et al., 2009). Two distance cell arrays (one for each of the principal axes $\vec{x}$ and $\vec{y}$) receive input from a population of grid cells whose firing rates encode the current location, and two separate distance cell arrays (one for each of the principal axes $\vec{x}$ and $\vec{y}$) receive input from a population of grid cells whose firing rates encode the goal location. It has been suggested that distance cells may correspond to place cells in the dentate gyrus (DG; Huhn et al., 2009), which significantly outnumber those in the CA3 and CA1 subfields (~1×10^6 granule cells in rat DG and ~2.5×10^5 pyramidal cells in rat CA3 or CA1; Amaral and Lavenex, 2007) and whose properties have not been well characterised (Jung and McNaughton, 1993). However, distance cells in this model exhibit a band-like firing pattern, as they encode a series of allocentric locations along a specific 1D axis with a spatial periodicity equal to the capacity of the grid cell system.

Grid cells project to distance cells in each array with synaptic weights that are proportional to their mean firing rate at the allocentric location *a* encoded by that distance cell on the directional axis $\vec{x}$ or $\vec{y}$, i.e. $w_{DC,GC} \propto r_{j,i}(a)$ (Figure 5A; Equation S6), analogous to models of the grid cell to place cell transformation (Rolls et al., 2006; Solstad et al., 2006). The number of spikes fired by each grid cell is dictated by a Poisson process with a cosine tuned rate function (Equation S6) and a time window of 100ms.

Distance cells have a spatial resolution of 0.04m to give N_{DC}=12500 distance cells in each of the four arrays (corresponding to current and goal locations on the two axes $\vec{x}$ and $\vec{y}$), or a total of 50000 distance cells overall. Distance cells within each array are subject to winner-take-all dynamics implemented with an E%-max algorithm, such that cells only fire if their input is within *k*=1% of the maximum feed-forward excitation (de Almeida et al., 2009). Activity within each distance cell array is then normalised, such that the integrated activity across all distance cells within each 100ms time window is consistent across simulations.

Synaptic weights between each pair of (current and goal location) distance cell arrays and two read-out neurons for each principal axis $\vec{x}$ or $\vec{y}$ increase linearly in opposing directions along topographically ordered distance cells (Figure 5B). Hence, the relative firing rate of these two read-out neurons encodes the displacement between current and goal locations on that axis. The magnitude of translation vectors on each principal axis is decoded by fitting a line of regression to a plot of the difference in the firing rate of read-out cells against the true displacement vector along that axis across all *N*=1000 iterations (Fig S2A). We note that these putative read-out cells require both a very large and highly precise range of firing rates to represent planned trajectories, and this function might more easily be supported by populations of read-out cells.

Simulations demonstrate that the distance cell model can decode translation vectors between arbitrary current and goal locations in large-scale 2D space within a 100ms time window with a mean accuracy of <4cm (Fig S2B). Importantly, there is no correlation between the magnitude of the vector and the error with which it is decoded (r=0.017, p=0.60, Fig S2C), as distance cells evenly cover the arena.

The Vector Cell Models

In vector cell simulations, separate populations of vector cells decode the displacement between arbitrary start and goal locations in each direction along the principal axes $\vec{x}$ and $\vec{y}$. The spatial resolution of vector cells is pseudo-exponentially distributed in the 0 : 500m range (Fig S3A) to give a total of N_{VC}=1250 vector cells in each of four arrays (corresponding to each direction of movement along the two principal axes $\vec{x}$ and $\vec{y}$), or a total of 5000 vector cells overall. This is an order of magnitude smaller than the additional number or cells required by the distance cell model (see above), as the need for an intermediate representation of absolute location is eliminated. In turn, this allows the spatial resolution of vector cells to be reduced for greater encoded displacements, as translation vectors can be dynamically recalculated with increasing accuracy as the goal is approached (see also Erdem and Hasselmo, 2014). We note that this does not reduce the capacity of the grid cell system, it merely allows that capacity to be under-sampled at greater distances from the current location.

In these simulations, for each pair of *N*=1000 arbitrary start and goal locations, the current location is updated by 80% of the decoded vector length along each axis in each step, and then translation vectors are iteratively re-calculated, until the current location is within 1m of the true goal. In each step, vector cells within each pair of arrays encoding displacements along a single axis are subject to winner-take-all dynamics implemented with an E%-max algorithm, such that cells only fire if their input is within *k*=1% of the maximum feed-forward excitation (de Almeida et al., 2009). The overall translation vector is decoded by combining the average of all active vector cells along each axis $\vec{x}$ or $\vec{y}$, weighted by their firing rate.

In rate-coded vector cell simulations, each pair of vector cell arrays that encode displacement in either direction along the principal axis $\vec{x}$ or $\vec{y}$ receive input from a population of grid cells that encodes start and goal locations on that axis. Vector cells receive input from grid cell pairs within each module whose unwrapped phase difference corresponds to that absolute vector on that directional axis through multiplicative synapses, such that activity only reaches the vector cell if both grid cells in a start-goal pair are active (Figure 6A). The number of spikes fired by each grid cell in each step is dictated by a Poisson process with a cosine tuned rate function (Equation S6) and a time window of 100ms.

Simulations demonstrate that the rate-coded vector cell model can decode translation vectors between arbitrary current and goal locations in large-scale 2D space with a mean accuracy of <4cm (Fig S3B). These translation vectors are decoded rapidly, in the minimum possible number of iterative 100ms steps in all simulations (mean = 3.83 steps, range of 2-4 steps). The magnitude of error in the translation vector decoded in the first step correlates with the length of that vector (r=0.61, p<0.001; Fig S3C), due to the decrease in vector cell precision for greater vector lengths, while no such relationship is observed for the distance cell model (Fig S2C).

In phase-coded vector cell simulations, pairs of vector cell arrays that encode displacement in either direction along the principal axis $\vec{x}$ or $\vec{y}$ receive input from separate populations of grid cells that exhibit phase precession aligned with each axis. Only the grid cells within each module GC_b that exhibit the maximum firing rate at the goal location *b* fire within a single t_{theta}=100ms theta cycle. Their theta firing phase is drawn from a circular normal distribution with a mean $\bar{\emptyset}_\iota$ that is linearly correlated with the circular distance between the peak of that grid cell's firing field *b* and the current location *a* along that axis (Equation S7) and a circular standard deviation of $\mu = {}^{\pi}/_{6}$ rad (Figure S4A).

$$\bar{\emptyset}_\iota(GC_b) = 2\pi \frac{mod(b-a, s_i)}{s_i}$$

[Equation S7]

In these simulations, vector cells must be sensitive to specific spike timing input patterns, analogous to models of polychronous computation (Jeffress, 1948; Hopfield, 1995; Izhikevich, 2006). For simplicity, this is achieved by grid cells in each module projecting to vector cells through delay lines with transmission delays t_i that are proportional to the difference between the grid scale s_i and the encoded vector *d* according to Equation S8 (Figure S4B). The activity of each vector cell is then taken as the resultant vector length of input spike phases from grid cells encoding the goal location in all modules.

$$t_i(d) = \frac{mod(\frac{s_i}{2} - d, s_i)}{s_i} t_{theta}$$

[Equation S8]

Simulations demonstrate that the phase-coded vector cell model can decode translation vectors between arbitrary current and goal locations in large-scale 2D space with an accuracy of <4cm (Fig S4C). These translation vectors are decoded rapidly, in the minimum number of iterative 100ms steps in all simulations (mean = 3.82 steps, range of 2-4 steps). Again, the magnitude of error correlates with the size of decoded vectors (r=0.66, p<0.001; Fig S4D), due to the decrease in vector cell precision for greater vector lengths, while no such relationship is observed for the distance cell model (Fig S2C).

We note that there is little empirical evidence for the existence of delay line connectivity of the kind employed here in cortex (but see McKenzie et al., 2014). However, the same functionality could be achieved by dendritic integration that allows the sequence and relative timing of inputs to be discriminated (Branco et al., 2010; Vaidya and Johnston, 2013). Interestingly, differences in theta phase and ionic conductances along the dendrite could affect the integration of inputs at the soma to make hippocampal pyramidal neurons sensitive to the relative timing and distribution of their inputs (Bullock et al., 1990; Vaidya and Johnston, 2013). It is important to note that this model is insensitive to the actual underlying oscillatory frequency against which phase precession occurs, so that a more realistic time constant of dendritic integration (i.e. <10ms) could be used if phase precession transiently occurred against a higher frequency oscillation. Moreover, the model does not rely on linear phase precession, or on phase precession covering the full range of 2π radians - any constant, monotonic relationship between position and theta firing phase is sufficient to support the decoding of translation vectors.

One experimental prediction of the phase-coded vector cell model is the existence of separate populations of grid cells that exhibit phase precession along specific 1D axes. Phase precession has been best characterised for non-directional grid cells and corresponds to distance travelled through the firing field irrespective of direction (Hafting et al., 2008; Reifenstein et al., 2012; Climer et al., 2013; Jeewajee et al., 2014). However, a subpopulation of conjunctive head-direction modulated grid cells in the deeper layers of mEC do show reliable theta phase precession (Hafting et al., 2008; Newman and Hasselmo, 2014; Reifenstein et al., 2014) which might serve this purpose (but see Climer et al., 2013).

The Linear Look Ahead Model

In the linear look ahead model, four linear look ahead events are required to compute translation vectors between arbitrary start and goal locations in 2D space, corresponding to the sequential exploration of each direction along the two principal axes $\vec{x}$ and $\vec{y}$. During each linear look ahead event, activity is initiated by setting grid cell firing rates in each module to match those at the start location and then updating grid cell firing in each subsequent time step according to simulated movement away from the current location at a constant speed along that directional axis.

The distance between start and goal locations in either direction along each principal axis is decoded from the time elapsed between the initiation of linear look ahead activity and firing activity in the

place cell encoding the goal location. This could be encoded by the firing rate of a cell that integrates total activity in a single grid cell module, or the grid cell system as a whole, over the course of that linear look ahead event (for a similar proposal during active navigation, see Kubie and Fenton, 2009). Some layer II mEC cells are known to exhibit bistable persistent spiking, such that their firing rate reflects a stable integral of the depolarisation reaching the cell (Klink and Alonso, 1997; Egorov et al., 2002; Fransen et al., 2006).

Overall translation vectors in 2D space are constructed by combining the decoded distances and direction along each of the principal axes. We note that the principal axes along which linear look ahead is performed need not be consistent between events, nor must the same axes be explored in either direction – any set of non-collinear, directional axes that are sufficient to triangulate an arbitrary location in 2D space will suffice. Moreover, as linear look ahead events are carried out sequentially, these could correspond to successive head directions of the animal during scanning behaviour.

In these simulations, we set a time step *dt*=5ms that corresponds to the integration time constant of a post-synaptic neuron – that is, grid cells across modules that fire within a 5ms time window encode a single location that can be decoded by an output place cell. We then set a constant, virtual speed for linear look ahead events of v_{sweep}=8ms^{-1} (Davidson et al., 2009), which subsequently determines the distance increment between each integration time step and therefore the spatial resolution of output place cells (0.04m in this case). Synaptic weights between grid cells and place cells are proportional to the grid cell firing rate at the peak of the place cell firing field along the axis $\vec{x}$ or $\vec{y}$, i.e. $w_{DC,GC} \propto r_{j,i}(a)$ (Equation S6). The number of spikes fired by each grid cell in each time step is dictated by a Poisson process with a cosine tuned rate function (Equation S6) and the time window of *dt*=5ms. Activity within each place cell array is subject to winner-take-all dynamics implemented with an E%-max algorithm such that cells only fire if their input is within *k*=1% of the maximum feed-forward excitation (de Almeida et al., 2009).

Simulations demonstrate that the linear look ahead model can decode translation vectors between arbitrary current and goal locations in 2D space with an accuracy of <4cm (Fig S5A). Like the distance cell model, there is no correlation between the magnitude of the vector and the error with which it is decoded (r=0.017, p=0.60, Fig S5B), as the firing fields of output place cells evenly cover the arena. However, the time taken to decode translation vectors is correlated with their magnitude, and can be an order of magnitude larger than the direct decoding models (Fig S5C). For example, linear look ahead at a constant speed of v_{sweep}=8ms^{-1} takes approximately one minute to decode a single 500m vector, and four of these linear look ahead events, corresponding to independent searches in each direction along two non-collinear axes, are required to compute a 2D translation vector. It is possible to increase the speed of linear look ahead activity, but at the cost of precision, as a broader population of grid cells fire within each integration time window. Moreover, the capacity of the grid cell system will be reduced if smaller scales can no longer contribute to encoding distinct locations, as will occur if all grid cells in those modules fire within the time window of post-synaptic integration. Similarly, if the distance to the goal is not known a priori, then the speed of linear look ahead activity cannot be tailored to match the scale of the decoded vector (but see Erdem and Hasselmo, 2014).

We note that linear look ahead activity in the grid cell network is phenomenologically similar to hippocampal replay and preplay events, during which trajectories often originate close to the animal and travel coherently through space (Foster and Wilson, 2006; Davidson et al,. 2009; Pfeiffer and Foster, 2013). However, preplay trajectories in place cells also appear to move directly towards future goals (Pfeiffer and Foster, 2013; Olafsdottir et al., 2015), which requires a priori knowledge of the direction towards those locations. Hence, it is possible that the planning of a complete navigational trajectory using linear look ahead might take place in two stages. First, linear look ahead in grid cells could be used to compute a direct vector to the goal location. During this period, place cell read-out activity would be driven by grid cell firing and used to ascertain the point at which grid cells encoding the goal location across modules became simultaneously active. Next, preplay activity in place cells could be used to sequentially check the sensory attributes of each location along that direct vector, in order to identify any potential hazards or impediments to navigation. During this period, place cell preplay activity would resemble a trajectory moving directly away from the current location towards, and terminating at, the goal (Pfeiffer and Foster, 2013; Olafsdottir et al., 2015). We note that this latter stage could also be used to simulate the sensory content of planned trajectories after a translation vector was computed from grid cell representations using any of the direct decoding models, and would be consistent with experimental indications that sharp wave/ripple (SWR) dynamics originate in the recurrent collaterals of CA3 (Chrobak and Buzsaki, 1996; Sullivan et al., 2011).

Supplemental References

Amaral D, Lavenex P (2007) Hippocampal Neuroanatomy. The Hippocampus Book, Oxford University Press, Oxford, UK

Branco T, Clark BA, Häusser M (2010) Dendritic discrimination of temporal input sequences in cortical neurons. Science 329: 1671-1675

Bullock TH, Buzsáki G, McClune MC (1990) Coherence of compound field potentials reveals discontinuities in the CA1-subiculum of the hippocampus in freely-moving rats. Neuroscience 38: 609-619

Chrobak JJ, Buzsáki G (1996) High-frequency oscillations in the output networks of the hippocampal-entorhinal axis of the freely behaving rat. Journal of Neuroscience 16: 3056-3066

de Almeida L, Idiart M, Lisman JE (2009) A second function of gamma frequency oscillations: an E%-max winner-take-all mechanism selects which cells fire. Journal of Neuroscience 29: 7497-7503

Egorov AV, Hamam BN, Fransén E, Hasselmo ME, Alonso AA (2002) Graded persistent activity in entorhinal cortex neurons. Nature 420: 173-178

Fransen E, Tahvildari B, Egorov AV, Hasselmo ME, Alonso AA (2006) Mechanism of graded persistent cellular activity of entorhinal cortex layer V pyramidal neurons. Neuron 49: 735-746

Hopfield JJ (1995) Pattern recognition computation using action potential timing for stimulus representation. Nature 376: 33-36

Izhikevich EM (2006) Polychronisation: Computation With Spikes. Neural Computation 18: 245-282

Jeffress LA (1948) A Place Theory of Sound Localisation. Journal of Comparative and Physiological Psychology 41: 35-39

Jung MW, McNaughton BL (1993) Spatial selectivity of unit activity in the hippocampal granular layer. Hippocampus 3: 165-182

Klink R, Alonso A (1997) Muscarinic modulation of the oscillatory and repetitive firing properties of entorhinal cortex layer II neurons. Journal of Neurophysiology 77: 1813-1828

Kubie JL, Fenton AA (2009) Heading-vector navigation based on head-direction cells and path integration. Hippocampus 19: 456-479

McKenzie IA, Ohayon D, Li H, de Faria JP, Emery B, Tohyama K, Richardson WD (2014) Motor skill learning requires active central myelination. Science 346: 318-322

Newman EL, Hasselmo ME (2014) Grid cell firing properties vary as a function of theta phase locking preferences in the rat medial entorhinal cortex. Frontiers in Systems Neuroscience 8: 193

Sullivan D, Csicsvari J, Mizuseki K, Montgomery S, Diba K, Buzsáki G (2011) Relationships between hippocampal sharp waves, ripples, and fast gamma oscillation: influence of dentate and entorhinal cortical activity. Journal of Neuroscience 31: 8605-8616

Towse BW, Barry C, Bush D, Burgess N (2014) Optimal configurations of spatial scale for grid cell firing under noise and uncertainty. Philosophical Transactions of the Royal Society B: Biological Sciences 369: 20130290

Vaidya SP, Johnston D (2013) Temporal synchrony and gamma-to-theta power conversion in the dendrites of CA1 pyramidal neurons. Nature Neuroscience 16: 1812-1820

Wei XX, Prentice J, Balasubramanian V (2013) The sense of place: grid cells in the brain and the transcendental number e. arXiv: 1304.0031
